# KlebPhaCol: a community-driven resource for *Klebsiella* research identified a novel phage family

**DOI:** 10.1093/nar/gkaf1122

**Published:** 2025-11-20

**Authors:** Daniela Rothschild-Rodriguez, Kai S Lambon, Simran Krishnakant Kushwaha, Sofya K Garushyants, Moritz Ertelt, Agnieszka Latka, Ana Rita Costa, Anna Mantzouratou, Claire King, Dimitri Boeckaerts, Elizabeth Sheridan, Eugene V Koonin, Francesca Merrick, Francis Drobniewski, Ilaria De Angelis, Kordo Saeed, Macy Martin, J Mark Sutton, Matthew E Wand, Michael Andrew, Morgen Hedges, Stan J J Brouns, Pieter-Jan Haas, Sophie T Lawson, Stephen M E Fordham, Yan-Jiun Lee, Yi Wu, Yves Briers, Peter Braun, Peter R Weigele, Franklin L Nobrega

**Affiliations:** School of Biological Sciences, University of Southampton, Southampton SO17 1BJ, Hampshire, United Kingdom; School of Biological Sciences, University of Southampton, Southampton SO17 1BJ, Hampshire, United Kingdom; School of Biological Sciences, University of Southampton, Southampton SO17 1BJ, Hampshire, United Kingdom; Computational Biology Branch, Division of Intramural Research, National Library of Medicine, National Institutes of Health, Bethesda, MD 20894, United States; Fraunhofer Institute for Translational Medicine and Pharmacology ITMP, Immunology, Infection and Pandemic Research IIP, Munich 60596, Germany; Institute of Infectious Diseases and Tropical Medicine, LMU University Hospital, LMU Munich 80802, Germany; Department of Biotechnology, Ghent University, Ghent 9000, Belgium; Department of Pathogen Biology and Immunology, University of Wroclaw, Wroclaw 51-148, Poland; Department of Bionanoscience, Delft University of Technology, Delft 2629 HZ, The Netherlands; Life and Environmental Sciences, Bournemouth University, Bournemouth BH12 5BB, United Kingdom; School of Biological Sciences, University of Southampton, Southampton SO17 1BJ, Hampshire, United Kingdom; Department of Biotechnology, Ghent University, Ghent 9000, Belgium; KERMIT, Department of Data Analysis and Mathematical Modelling, Ghent University, Ghent 9000, Belgium; Life and Environmental Sciences, Bournemouth University, Bournemouth BH12 5BB, United Kingdom; Department of Infectious Diseases, Imperial College, London W12 0BZ, United Kingdom; Computational Biology Branch, Division of Intramural Research, National Library of Medicine, National Institutes of Health, Bethesda, MD 20894, United States; School of Biological Sciences, University of Southampton, Southampton SO17 1BJ, Hampshire, United Kingdom; Life and Environmental Sciences, Bournemouth University, Bournemouth BH12 5BB, United Kingdom; Department of Infectious Diseases, Imperial College, London W12 0BZ, United Kingdom; Department of Biotechnology, Ghent University, Ghent 9000, Belgium; Department of Infection, Microbiology, University Hospital Southampton, Southampton SO16 6YD, United Kingdom; School of Biological Sciences, University of Southampton, Southampton SO17 1BJ, Hampshire, United Kingdom; United Kingdom Health Security Agency (UKHSA), Porton Down, Salisbury, Wiltshire SP4 0JG, United Kingdom; Institute of Pharmaceutical Sciences, King’s College London, London WC2R 2LS,United Kingdom; United Kingdom Health Security Agency (UKHSA), Porton Down, Salisbury, Wiltshire SP4 0JG, United Kingdom; School of Biological Sciences, University of Southampton, Southampton SO17 1BJ, Hampshire, United Kingdom; School of Biological Sciences, University of Southampton, Southampton SO17 1BJ, Hampshire, United Kingdom; Department of Bionanoscience, Delft University of Technology, Delft 2629 HZ, The Netherlands; Medical Microbiology, University Medical Center Utrecht, Utrecht University, Utrecht 3584 CX, Netherlands; United Kingdom Health Security Agency (UKHSA), Porton Down, Salisbury, Wiltshire SP4 0JG, United Kingdom; Institute of Pharmaceutical Sciences, King’s College London, London WC2R 2LS,United Kingdom; Life and Environmental Sciences, Bournemouth University, Bournemouth BH12 5BB, United Kingdom; Research Department, New England Biolabs, Inc., Ipswich, Massachusets 01938, United States; School of Biological Sciences, University of Southampton, Southampton SO17 1BJ, Hampshire, United Kingdom; Department of Biotechnology, Ghent University, Ghent 9000, Belgium; Fraunhofer Institute for Translational Medicine and Pharmacology ITMP, Immunology, Infection and Pandemic Research IIP, Munich 60596, Germany; Institute of Infectious Diseases and Tropical Medicine, LMU University Hospital, LMU Munich 80802, Germany; Research Department, New England Biolabs, Inc., Ipswich, Massachusets 01938, United States; School of Biological Sciences, University of Southampton, Southampton SO17 1BJ, Hampshire, United Kingdom

## Abstract

The growing threat of multidrug-resistant *Klebsiella pneumoniae*, coupled with its role in gut colonisation, has intensified the search for new treatments, including bacteriophage therapy. Despite increasing documentation of *Klebsiella-*targeting phages, clinical applications remain limited, with key phage–bacteria interactions still poorly understood. A major obstacle is fragmented access to well-characterised phage–bacteria pairings, restricting the collective advancement of therapeutic and mechanistic insights. To address this gap, we created the Klebsiella Phage Collection (KlebPhaCol), an open resource comprising 52 phages and 74 *Klebsiella* isolates, characterised at phenotypic and genomic levels. These phages span six families—including a novel family, *Felixviridae*, associated with the human gut—and target 20 sequence types (including ST258, ST11, and ST14) and 19 capsular-locus types (including KL1 and KL2), across 6 *Klebsiella* species. Freely accessible at www.klebphacol.org, KlebPhaCol invites the scientific community to both use and contribute to this resource, fostering collaborative research and a deeper understanding of *Klebsiella-*phage interactions beyond therapeutic use.

## Graphical Abstract

**Graphical Abstract:**
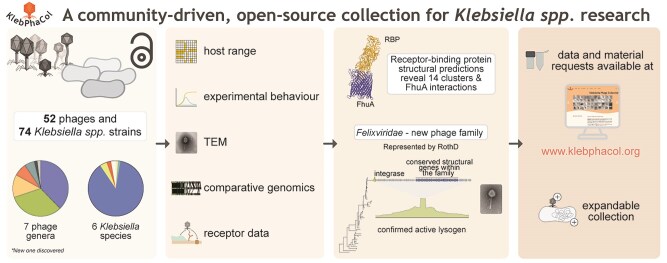

## Introduction

The global rise of antimicrobial resistance (AMR) has prompted urgent action to develop new, effective therapies [1–6], with bacteriophage (phage) therapy emerging as a promising option [7, 8]. Phages, as natural predators of bacteria, can precisely target bacterial pathogens, but a reliable pipeline from phage isolation to clinical application remains elusive [9]. Key challenges include limited regulatory frameworks and gaps in understanding phage-bacteria and phage–host interactions, which are essential for developing safe and reliable therapies [10].

*Klebsiella pneumoniae*, a multidrug-resistant pathogen and one of the six “ESKAPE” organisms (*Enterococcus faecium, Staphylococcus aureus, Klebsiella pneumoniae, Acinetobacter baumannii, Pseudomonas aeruginosa*, and *Enterobacter* spp.), exemplifies these challenges. Known for causing severe infections, *K. pneumoniae* has developed resistance to last-resort antimicrobials [11], including carbapenems [12], making it a high priority target for new antimicrobials [13]. *K. pneumoniae* infections, including pneumonia, sepsis, and liver abscess, are often acquired in hospital settings but are also found in community-acquired cases, especially involving hypervirulent strains [14–20]. The role of this pathogen in chronic gut colonization has further implicated it in gut conditions like inflammatory bowel disease (IBD) [21, 22] and primary sclerosing cholangitis [23], establishing *K. pneumoniae* as a significant gut-associated pathobiont.

A major challenge in phage therapy against *K. pneumoniae* is its highly variable capsule polysaccharide (K-types), with over 180 distinct types now genomically identified [24–27] and associated with different species [28] and virulence traits [29]. The diversity complicates treatment because capsule-specific phages, which depend on capsule polysaccharides to bind and infect cells, often have limited host ranges [30–32]. While some phages can bind alternative receptors like the O-antigen [33], capsule diversity remains a critical barrier. Beyond receptor diversity, bacterial defence systems and mobile genetic elements can further restrict phage efficacy [34–37]. These multifaceted interactions highlight the need for well-characterised phage collections, which can enable researchers to systematically study and address obstacles to successful therapy. Several collections of *Klebsiella* phages have been reported in the literature [33, 34, 38–42]. While these mark milestones in the field, there remains a fundamental need for centralising and standardising resources to make them easily accessible for the academic and clinical communities. Standardised, referenced collections, such as the BASEL collection for *Escherichia coli* [43, 44] phages or the CEPEST collection for *Pseudomonas putida* phages [45], demonstrate how accessible resources can foster shared advancements. Addressing this need, we present the Klebsiella Phage Collection (KlebPhaCol), an open-source collection that contains 52 phages and 74 *Klebsiella* strains, each extensively characterised. The open-source nature of KlebPhaCol (available at klebphacol.org) invites the scientific and medical community to contribute additional isolates and data, fostering and evolving this community-driven platform. In addition to informing phage therapy, this collection can be utilised to study fundamental aspects of phage–bacteria interactions. By centralising and sharing these resources, KlebPhaCol aims to bridge current gaps, empowering the scientific community to collectively advance research on *Klebsiella* and its phages for both therapeutic and broader biological insights.

Materials and methods

 

### Phage isolation and purification

Numbered phages (e.g. Roth01) were sourced from hospital wastewater effluent collected at the University Medical Centre Utrecht in the Netherlands in 2020 as previously described [46], while lettered phages (e.g. RothD) were sourced from effluent collected at Portswood in Southampton, United Kingdom in 2021. Thirty-two isolates with clinically relevant sequence types (ST) were used as isolation hosts (Supplementary Table S1). Based on ST grouping, seven enrichment cultures were produced: (i) ST11 (*n* = 5), (ii) ST101 (*n* = 5), (iii) ST15 (*n* = 4), (iv) ST258 (*n* = 5), (v) ST14 (*n* = 6), (vi) ST323 (*n* = 2), and (vii) the remaining ST-types [ST489 (*n* = 1), ST86 (*n* = 1), ST38 (*n* = 1), and ST23 (*n* = 2)]. Fifty microlitres of each overnight culture grown in Lysogeny Broth (LB; Formedium LB-Broth Lennox) were added to each respective enrichment containing 50 ml of LB and 50 µl of the phage source filtrate. Enrichments were incubated overnight at 37°C and shaking at 180 rpm, and then centrifuged (8000 × *g*, 20 min, 4°C) and filter-sterilized (0.45/0.22 µm PES). Five microlitres of the resulting supernatants were spot-tested for the detection of phage against all 32 isolates using a double-layer agar technique (top agar 0.6%) [47]. Susceptible isolates were subsequently plated with serially diluted phages to identify distinct plaque morphologies, which were then single picked with sterile toothpicks, dotted, and spread with sterile paper onto fresh bacterial lawns to purify the phages. This latter step was repeated twice to obtain a consistent plaque morphology. Individually purified phages were then propagated in LB with their respective host, centrifuged, filter-sterilized, and stored at 4°C.

 

Phage host-range

Five microlitres of undiluted phage lysates were first spotted onto double-layer agar plates for each of the 74 *Klebsiella spp*. strains tested. Phages that showed some form of lysis were then re-tested using 10-fold serial dilutions of stocks normalized at 10^8^ PFU/ml. These were then spotted onto double-layer agar plates with respective bacterial lawns. The plates were incubated overnight at 37°C, and phage plaques were observed to distinguish between productive infection (lysis with individual plaques), no infection (lack of plaques), and undetermined lysis (opaque lysis without individual plaques). Assays were conducted in both LB and Tryptic Soy Broth (TSB; Hach Bacto^™^ Tryptic Soy Broth) media. Unless otherwise stated, all other phage assays were done in LB.

 

### Plate reader liquid assays

Overnight bacterial cultures of strains susceptible to phages infecting strains of the ST323 sequence type (on solid agar) were diluted 1:100 in LB and incubated at 37°C at 180 rpm up to an OD_600_ of ∼0.3. Cultures were normalized to an OD_600_ of 0.1 and dispensed into a 96-well plate. Experimental wells had phage added at the desired high ($ \ge $1) or low ($ \le $1) multiplicity of infection (MOI). Growth was monitored every 10 min for 900 min in a Spectrostar Nano (BMG Labtech, UK) plate reader at 37°C, non-shaking, in either aerobic or anaerobic conditions. To ensure the latter, all holes in the plate reader were plugged as specified by the manufacturer, and N_2_ gas was consistently pumped at a low rate to eliminate any oxygen for the entirety of the experiment. Growth curves were converted to area under the curve (AUC) using GraphPad Prism.

 

### Bacteriophage insensitive mutants

Bacteriophage insensitive mutants (BIMs) of capsule-deficient strain 51 851 were obtained after spot tests with different phages. A random selection of 17 BIMs from 51851 strain were cultured for sequencing and for phage re-testing. Strain DNA was extracted and sequenced as described below, and reads were mapped against the wild-type (WT) 51851 strain to confirm mutations. Phage susceptibility re-testing was performed in a 96-well plate by growth of the BIMs in LB broth at 37°C with the addition of phage. Growth of the bacterial isolate in the absence of phage was used as a positive control. OD_600_ readings were taken every hour up to 20 h using a CLARIOstar Plus plate reader (BMG Labtech, UK). Growth curves were analysed and, where there was no observable difference in the presence and absence of phage, the BIM was classified as resistant.

 

### Phage sequencing, assembly, and annotation

Phage DNA was extracted using phenol–chloroform as previously described [48]. DNA from 32 phages were sequenced by BMKGene (Germany). For this, sequencing libraries were prepared using the Reseq-M DNA kit and paired-end reads (2 × 150 bp) were generated in the Illumina Novaseq 6000 platform (Illumina, USA). Approximately 3–4 Gb of clean sequencing data were produced for each sample, with sequencing depth >5000×. The remaining DNA was sequenced by the UKHSA-GSDU (UK health security agency Genomic Services and Development Unit) (see Supplementary Table S2). Libraries were prepared using the Nextera DNA flex library prep kit (Illumina, USA) according to manufacturer’s instructions and reads (2 × 150 bp) were generated in the Illumina HiSeq 2500 platform (Illumina, USA). A minimum of 150 Mb of Q30 quality data were obtained for each sample.

Unless otherwise stated, CLC Genomics Workbench v23.0.1 (Qiagen, Germany) was used for quality checks, sequence trimming (quality limit = 0.05) and genome assembly. Reads were subsampled then assembled with the *de novo* assembler tool (default parameters) on CLC. Sequencing reads for 13 phages (see Supplementary Table S2) were checked for quality using FastP [49] v.0.12.4 and Soapnuke [50] v2.1.7 with default parameters. For these specified phages, reads were sampled and trimmed using Seqtk v1.3.0 and then assembled using SPAdes [51] v3.13.0. All produced assemblies were manually inspected on Bandage v0.8.1 and Geneious Prime v11.0.18 + 10 (https://www.geneious.com/).

The phages’ closest relative was determined as the top hit according to the maximum score provided by BLASTn (March–June 2023 and February 2024, https://blast.ncbi.nlm.nih.gov/Blast.cgi [52]). Assemblies were mapped to fastq reads to check for irregularities using Qualimap2 v2.3 [53].

The start of the phage genome was adjusted to allow phage comparisons with canonical phages, by choosing a conserved feature to serve as gp1 or “start-site” for each of the families represented in the collection. These genome start-sites were chosen based on historical precedent and/or biology of infection and/or DNA packaging. For the *Straboviridae*, which includes the well-known *Escherichia* phage T4 (genus *Tequatrovirus*), and the genera *Jiadodavirus* and *Slopekvirus*, the *rIIA* gene was chosen, in accordance with NCBI record NC_000866.4 [54]. In cases where a landmark feature overlapped another gene, the nearest non-CDS region 5′ or 3′ to *rIIA* was chosen to avoid software artefacts. The *Demercviridae* contains a landmark member, *Escherichia* phage T5 (genus *Tequintavirus*, NC_005859.1) where the first-step-transfer region encoding *dmp*, a 5′-deoxyribonucleotidase, is first to enter the cell upon infection [55, 56]. The *Drexlerviridae* includes phage T1 (genus *Tunavirus*, NC_005833.1), which is known to have terminal repeats at the genome ends [57]. For Roth32, infection by coliphage T7 (NC_001604.1), a member of the genus *Teseptimavirus* of this family, an ∼850 bp segment of the virion DNA enters the cell first [58]. For phages from the *Drulisvirus* genus, the small terminase subunit was defined as the start of the genome, a convention built into some automated annotation pipelines [59]. For the novel *Felixviridae* family, the core region [genes gp1 (hypothetical protein) to gp24 (structural protein)] was defined as the start. Manual assignment of nucleotide start-site was accomplished using Geneious Prime v11.0.18 + 10 (https://www.geneious.com).

Final phage genome length and GC content were determined by EMBOSS v6.6.0.0 [60]. Phage sequences were then inputted to PhageTerm [61] via the Center for Phage Technology galaxy portal (https://phage.usegalaxy.eu/) to identify phage termini and packaging.

Phage coding sequences (CDS) were predicted with PHANOTATE v2019.08.09 [62] using translation table 11, then annotated using multiPHATE v2.0.2 [63] against the NCBI database selecting annotations with an Evalue threshold of 0.001. Transfer RNA (tRNA) genes were identified using tRNAscan-SE v2.0.12 [64] via multiPHATE and confirmed using ARAGORN v1.2.41 [65], although tRNAscan-SE findings were kept. Phages were also annotated with the Pharokka [59], Phold [66], and Domainator v0.7 [67] to highlight additional domain and gene functions (Domainator annotations are available on Figshare DOI 10.6084/m9.figshare.27794211). Default Pharokka annotations were manually curated using Geneious Prime v11.0.18 + 10 (https://www.geneious.com). In cases where Pharokka and Phold produced conflicting results, annotations were curated conservatively, either retaining the most likely hit or replacing the entry with “hypothetical protein.” Manual curation focused primarily on the conserved region of RothD (gp1-gp24) and on correctly clearly erroneous annotations, as previously recommended [68]. Anti-defence proteins were predicted using AntiDefenseFinder [69]. Potential AMR and virulence genes in the phages were predicted using the Comprehensive Antibiotic Resistance Database (CARD) [70] and the Virulence Factor Database (VFDB) [71], respectively.

The lifestyle of the phages was predicted using Bacphlip [72]. Phage receptor-binding proteins (RBPs) and depolymerases were identified using RBPdetect v3.0.0 [73] and DepoScope v1.0.0 [74], respectively. Structures of the proteins from RothD’s core genome were predicted using Seq2symm [75] and ColabFold [76], and subsequently compared to existing structures using Foldseek search [77].

 

### Phage receptor-binding proteins

The symmetry of all RBPs identified by RBPdetect (*n* = 207) was predicted using Seq2Symm and then used to setup structure prediction with ColabFold. All resulting structures were clustered with Foldseek easy-cluster using default parameters. For each cluster, a representative was compared to the PDB [78] and AlphaFold2 [79] databases using Foldseek search. Only clusters with multiple members were retained for further analysis, except cluster 8 (a singleton with Roth44 gp50), which was included due to its high structural similarity to Roth47 gp52 from cluster 9, aside from its distinct “tip” - a feature that may account for their differing host ranges. Additionally, RBPs assigned with predicted depolymerase function by DepoScope were manually curated for depolymerase activity based on conserved structural elements according to the method described previously [80] (Supplementary Text). We used several structural prediction tools including (Phyre2 [81], HHpred [82], and AlphaFold) to increase confidence in RBP structures and interactions. When a RBP is not identified as a depolymerase, the RBP is presumed to be a tail fibre that binds to the bacterial receptor.

 

### Phage comparative genomics

To assign phage taxonomy, genomes were run on PhageGCN [83] web server and confirmed by clustering on vContact2 v0.11.3 [84], using the default database and visualised using Cytoscape v3.10.2 [85]. Intergenomic similarity was calculated using VIRIDIC [86] on the web server and similarity matrices were re-plotted using Pheatmap v1.0.12 [87]. Phylogenetic analyses were produced by the VICTOR web server with default settings, which employs the Genome-BLAST Distance Phylogeny method adapted to bacteriophages [88]. Tree images were rendered and rooted at the midpoint using iTOL v6.1.1. (https://itol.embl.de/) [89] Synteny plots were produced by Clinker [90] on their web server.

 

### Bacterial DNA extraction and genome assembly

Seventy-four clinical isolates of *Klebsiella spp*. were used in this study. Sixty-five are *K. pneumoniae*, two *K. oxytoca*, two *K. variicola*, one *K. aerogenes*, one *K. pneumoniae subsp. ozaenae*, and three *K. quasipneumoniae*, see Supplementary Table S1 for isolate characteristics. Thirty-two strains were used for phage isolation enrichment cultures, but only seventeen continued as isolation hosts (Supplementary Table S1). Genomic DNA for the *Klebsiella* strains were extracted using the GeneJet Genomic DNA Kit (Thermo Scientific, UK) or the Wizard DNA Extraction Kit (Promega, UK) according to the manufacturer’s instructions. DNA was quantified by a Qubit fluorometer using the high sensitivity dsDNA Kit (Invitrogen, UK) and Nanodrop (Thermo Scientific, UK). DNA was prepped and sequenced by UKHSA-GSDU as described above. Fastq reads were quality trimmed using Trimmomatic v0.39 [91] and draft chromosome contigs were assembled using SPAdes v3.15.3 filtering out contigs <1 kb.

 

### Bacterial genome analyses

Genomes were annotated using Prokka v1.14.6 [92]. Strains were classified by their sequence (ST) and capsular locus (KL) types using the multilocus sequence typing (MLST) database (Center for Genomic Epidemiology, https://cge.food.dtu.dk/services/MLST/) and Kaptive v3.1.0 [26, 93, 94] using the K locus primary reference database, respectively. Strains from the *K. pneumoniae* species complex (KpSC) [19] were also classified by the cgMLST-based Life Identification Numbers (cgLIN codes) available via Pathogenwatch (https://pathogen.watch/) [95] to provide a better phylogenetic resolution and precision at a nomenclature-based level [96] (Supplementary Table S1). Strains were run through the Kleborate [25] pipeline to obtain virulence and resistance scores, and outputs were visualized using the Kleborate-Viz platform online (https://usegalaxy.eu/root?tool_id=kleborate) (no markers were found for strain 163575R). The phylogeny of the strains was calculated via PopPUNK v2.5.0 [97] using the default fitted model for *K. pneumoniae*. The tree was rendered in iTOL v6.1.1 (https://itol.embl.de/) [89].

The bacterial virulence factors, antibiotic resistance, and stress resistance genes were identified using Abricate v1.0.1 against the CARD [70], NCBI AMRFinderPlus [98] and VFDB [71] databases. Prophage regions were identified using Phigaro v2.2.6 [99] on default mode. The defence systems in the genomes were identified using PADLOC v1.1.0 [100] and DefenseFinder v1.0.9. [101]. Incomplete defence systems, VSPR and PDC, were removed from quantification analyses but are included in Supplementary Table S1. Correlation analyses between encoding defence systems and host range outcomes were conducted with Spearman’s correlation and plotted in RStudio v2024.04.2 using the ggplot2 [102] package.

Bacterial capsule loci (defined as the genetic region from *galF* to *uge*) [103] were manually assembled for isolation hosts (17 strains). Assembly was conducted by first looking for the more conserved regions of *galF* and *uge* genes and then individually checking and annotating other genes in Seq Builder v14.0.0 (DNAstar Lasergene). In some cases, due to transposon insertions within the CPS locus, it was not possible to generate one contig containing the complete locus; for such cases a string of n’s was artificially added to represent a break in the contigs.

 

### Antibiotic susceptibility

For clinical isolates obtained at the University Medical Centre Utrecht (see Supplementary Table S1), antibiotic susceptibility was determined as previously described [46]. For the remaining clinical isolates, the minimal inhibitory concentrations (MIC) for antibiotics and biocides were determined by UKHSA using a standard broth microdilution method at a starting inoculum of 5 × 10^5^ CFU/ml, Phoenix M50 system (BD Biosciences, USA) and EUCAST breakpoints, with the exception that 96-well polypropylene plates (Griener Bio-One, Ltd., Austria) were used instead of polystyrene plates to test colistin. Plates were scored by eye, looking for no visual growth and confirmed by OD_600_ measurement after 16–20 h with a 0.1 OD_600_ threshold using a CLARIOstar Plus plate reader (BMG Labtech, UK).

 

### Bacterial capsule characterisation

Isolation host strains (*n* = 17) were inoculated in LB or TSB broth and incubated overnight at 37°C, 180 rpm. Cultures were then spun at 3200 × *g* for 10 min and their pellets resuspended in 2 ml of 1× phosphate-buffered saline (PBS). The wash step was repeated once. Density gradients were prepared using Percoll^®^ (P4937, Sigma–Aldrich) at 30%, 60%, and 80% v/v (diluted in 1× PBS) [104]. One millilitre of each gradient was gently dispensed into fresh 15 ml falcon tubes using a 1 ml syringe and 1.5-inch needle. The 30% layer was pipetted first, followed by the 60% layer beneath it, and finally the 80% layer at the bottom. Six hundred microlitres of the prepped cells were then gently layered at the top of the gradient and samples were spun at 3000 × *g* for 30 min. The tubes were then imaged against a black background to visualise the capsule deposition.

 

### Genomic comparisons of *Felixviridae* phages

RothD was taken as the representative phage of the *Felixviridae* family. To determine the prevalence of *Felixviridae* phages within bacterial genomes given their temperate lifestyle, the BV-BRC (https://www.bv-brc.org/) bacterial strain database (*n* = 64 364; 25 384 complete high-quality *Klebsiella spp*. and 38 980 complete high-quality non-*Klebsiella* bacterial genomes), and associated metadata were retrieved (accessed July 2024). The core region and the full genome of RothD were independently searched against the downloaded bacterial genomes using command line BLASTn v2.15.0 with an *E*-value threshold of 0.005 with a -max_target_seqs parameter of 100 000. All hits were extracted and searched for prophage regions using Phigaro v2.4.0 [99] with default settings. The ten upstream and ten downstream genes from the hit region were extracted for analysis.

To investigate the predominance of *Felixviridae* in the average human gut, we first looked at the GPD hits used in the taxonomic characterisation of RothD (see above) and matched them with the GPD’s available metadata. The produced dataset was then analysed. Taxonomic characterisation of RothD revealed several relatives assembled from a singular study by Tisza *et al.* [105]. Thus, we gathered all returned hits from the online BLASTn server and matched accession queries to those coming from the mentioned study. This resulted in a total of 229/406 total hits matching their chronic disease dataset, of which 205/229 (90%) were high-confidence hits with an *E*-value ≤1e-08. We then matched these to the study’s metadata and analysed the resulting dataset.

The relative abundance of *Felixviridae* phages was calculated as follows. The quality filtered reads from a subset of 117 “healthy” human stool metagenomes from the Human Microbiome Project [106, 107] were retrieved and aligned to a set of 21 *Felixviridae* phage genomes using the end-to-end alignment mode of Bowtie2 v2.5.4 [108]. Bacterial reads were identified using Kraken2 v2.1.3 [109]. A count table of reads aligned to contigs and total number of reads per metagenome was generated with Samtools v1.20 [110] and imported into Rstudio v2024.04.2 + 764 for analysis. Packages ggplot2 [102] and ggbreak [111] were used for plots.

To further assess the prevalence and genomic signatures of *Felixviridae* phages, the metagenomic and metavirome datasets from four previous studies [112–115] were retrieved (*n* = 826 gut metagenome samples). A set of 54 *Felixviridae* genomes were curated, quality-checked with CheckV v1.0.3 [116], and dereplicated using dRep v3.4.2 [117]. Metagenomic reads were quality-filtered using fastp v1.0.1 [49], and host contamination was removed using BBMap v35.85 [118] against the hg38 reference genome. Reads were mapped to the dereplicated *Felixviridae* genomes using minimap2 v2.30 [119], and coverage/breadth was assessed using samtools v1.22.1 [110]. Genome-level ANI and coverage were calculated using fastANI v1.34 [120] to avoid overinterpretation from short gene-level hits. Strain-level diversity and population variation were profiled using inStrain v1.3.1 [121]. To distinguish lytic versus lysogenic presence, we used paired metagenome–metavirome datasets and confirmed lysogeny markers with PHASTER v4.0.0 [122]. Abundance was analysed as specified above.

 

### PCR detection

Primers were designed to target gp7, a hypothetical protein (or putative virion structural protein by Phold) that maintains a high conservation across the *Felixviridae*: Forward 5′-ATGTTCCGTCAGGGGAAGTTC-3′, Reverse 5′-AAGCCTGGTTGTTAAAACTGG-3′. Primers were synthesized by IDT. Reactions were done with OneTaq quick load (NEB M0486) according to the manufacturer’s instructions on a T100 Thermal Cycler (Bio-Rad, UK) and visualised on a 0.7% agarose gel. Positive control was RothD. Negative control was prepared using DNase-free water instead of template DNA. Specificity to *Felixviridae* phages was confirmed by also testing phages T4 (as a non-*Klebsiella* phage control) and Roth32 (as a *Klebsiella* phage control). The presence of *Felixviridae* phages in the environment was also tested by using filtered raw effluent from sewage plants in Southampton and Petersfield as well as ocean water from the Isle of Wight, UK (collected in the summer of 2024), filtered through a Vivaflow^®^ 200 cassette recirculation system (Sartorius, UK) and then through a 0.45 µm PES membrane. All controls (except for the negative control) were first heated at 95°C for 5 min to break virion capsules before adding as template DNA to the reactions. Polymerase Chain Reaction (PCR) products were cleaned and concentrated with the GeneJET PCR purification kit (Thermo Scientific, UK) and sent for sequencing at Plasmidsaurus (UK). Reads were trimmed and quality filtered using fastp v0.12.4 on the fastplong parameter and then mapped to RothD_gp7 using minimap2 v2.28-r1209. Coverage depth was obtained with Samtools v1.20 and Bedtools v2.30 [123] and results were imported in table format to RStudio v2024.04.2 + 764 and plotted using the ggplot2 [102] package.

 

### Lysogeny assays

Isolation strain 80 528 was grown in LB at 37°C, 180 rpm to an OD_600_ of 0.2. RothD was then added to an MOI of 1 and left to incubate overnight at 37°C. The following day, the cultures (80 528 + RothD, and 80 528 control) were spun down at 4000 × *g* for 10 min and washed with LB twice. The washed pellets were resuspended in 1 ml of LB and 10-fold dilutions were spotted onto LB agar plates and the plates incubated at 37°C. The remaining pellets were re-inoculated and re-infected with the same phage (except for the 80 528 control) and incubated overnight at 37°C, 180 rpm. This was repeated daily for a total of 5 days.

Five colonies of each sample per day were inoculated in 100 µl of sterile diH_2_O for PCR detection of TerL (Forward primer: 5′-GGCCGACATTTACCTACCCAC-3′, Reverse primer: 5′-TAGAGTGCGTCGCCGCTAC-3′) as described above. Colonies c2, c3, and c4 of each sample from day 1 were then inoculated overnight in LB, and bacterial DNA was extracted with the GeneJet Genomic DNA kit. DNA was sent for Illumina microbial sequencing at BMKGene (Germany). The produced raw reads were pre-processed with fastp v0.23.4 with default parameters to remove adapters and low-quality bases. Reads were aligned with bwa-mem2 v. 0.7.17-r1188 [124] to the combined *K. pneumoniae* 80 528 genome and RothD genome. To find the integration site in the bacterial genome and phage attachment site, discordant reads were extracted using samtools. Integration sites were identified where genomic regions presented high coverage of discordant reads (when mapped to the original 80 528 genome) in experimental samples (80 528 + RothD) compared to control samples (80 528). Similarly, the phage attachment site was determined as a peak of discordant reads coverage (when mapped to the RothD genome). The proportion of integrated phages was determined by dividing the number of discordant reads over the total coverage at the attachment site. Coverage plots were made using either the sorted bam files (samtools), or by obtaining coverage depth (bedtools); files were imported into Rstudio and plotted using Gviz [125] or ggplot2 [102], respectively.

To further confirm lysogeny, all five colonies per sample of day 1 and at least 2 colonies per sample of all other days, were grown in LB. RothD was 10-fold serially diluted, and spot tested on lawns made from each of the cultures following the double-layer agar assay described above. No plaques were expected in samples where the phage has integrated into the host genome.

## Results

### Overview of KlebPhaCol

KlebPhaCol is an open-source *Klebsiella* phage and strain collection comprising both biological materials (phages and strains) and associated data. The collection was designed to provide easy, cost-effective access to *Klebsiella* phages and strains to support collaborative research on phage–bacteria–host interactions and to facilitate the development of phage therapy. All data and access requests are managed through a dedicated platform, www.klebphacol.org, which allows users to explore the collection, download metadata tables (e.g. host range, capsule type, taxonomy, and isolation source), and request material via a simple online form.

The physical collection is hosted at the University of Southampton, where a curatorial team oversees sample storage, data curation and updates, and compliance with material sharing regulations. New phages and strains can be contributed by external researchers via the website; all submissions are manually reviewed to ensure metadata consistency and quality. The platform also includes a “board of discussions” feature (currently run via a mailing list), which facilitates community input on metadata standards, future features, and the integration of new tools or datasets.

**Figure 1.**
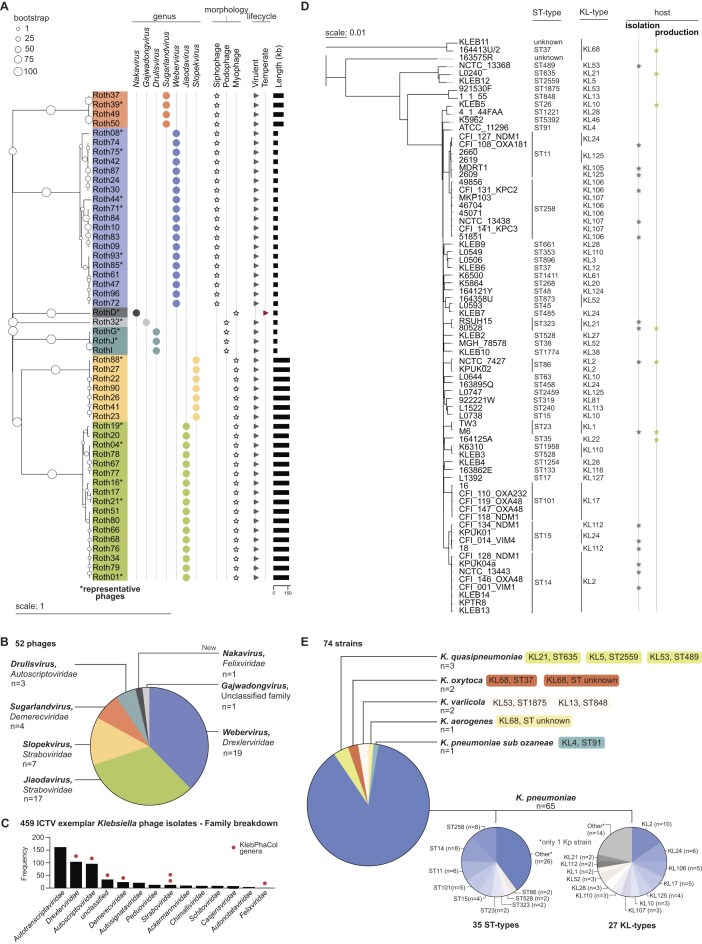
Overview of the Klebsiella Phage Collection (KlebPhaCol). (**A**) Phylogeny of the 52 phages of the collection and associated data. The phylogenetic tree was calculated using a Genome BLAST Distance Phylogeny method and midpoint rooted. (**B**) Quantification of the phage taxa covered by the phages in KlebPhaCol. (**C**) Distribution of *Klebsiella* phages families in the ICTV taxonomy (as of March 2025). Bars show the number of exemplar *Klebsiella* phages reported for each family in ICTV. Red circles indicate the families represented in KlebPhaCol. (**D**) Phylogeny of the 74 strains of the collection and associated data. Phylogenetic tree was produced by PopPUNK and midpoint rooted. (**E**) Quantification of the species of *Klebsiella*, their sequence type (ST), and capsule locus type (KL), included in KlebPhaCol. All trees were rendered in iTOL.

KlebPhaCol includes 52 phages isolated using 32 clinically relevant *Klebsiella spp*. strains (Fig. 1A, and Supplementary Tables S1 and S2). These phages were characterised at genomic (phylogeny, synteny, and gene content), phenotypic (plaque morphology and TEM imaging), and behavioural (one-step growth curves and host range) level. Detailed descriptions of the characteristics of these phages, organised by genera, can be found in the Supplementary Text. The KlebPhaCol phages span 7 genera across five of the 13 reported *Klebsiella* phage families, and the newly proposed *Nakavirus* genus and *Felixviridae* family [126] (Fig. 1B and C). To facilitate reproducibility and shareability, we selected seven strains as production hosts for the entire collection (Fig. 1D and Supplementary Table S1).

**Figure 2.**
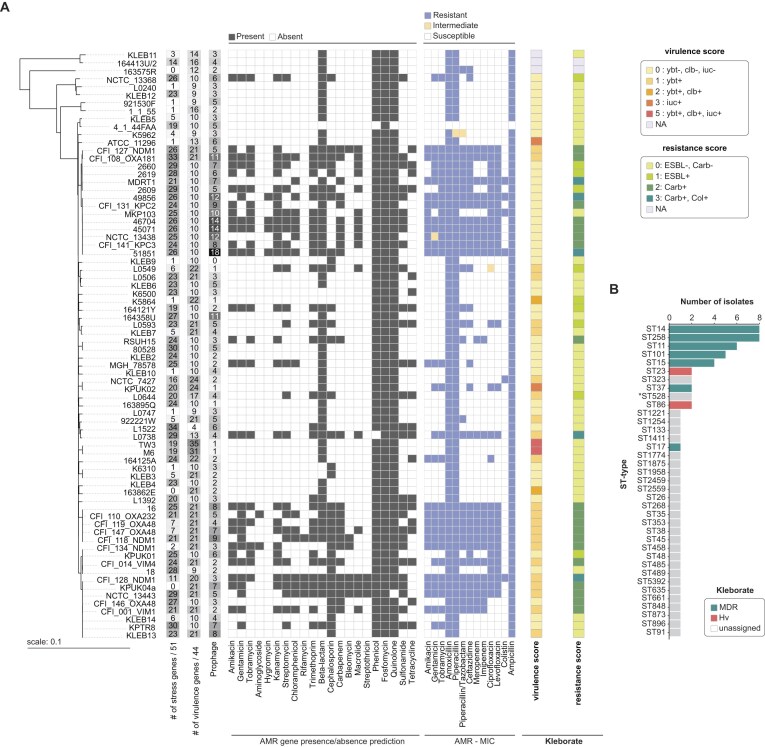
Characteristics of the 74 strains in KlebPhaCol. (**A**) Phylogeny of the 74 strains matched with the number of stress resistance genes (out of a total of 51), virulence genes (out of a total of 44), prophage predictions, and presence/absence of AMR genes. The MIC of 13 antibiotics was also tested for each strain and resistance patterns are illustrated in the coloured heatmap. Virulence and resistance scores were calculated with Kleborate. (**B**) Kleborate predictions of strains with multidrug resistance (MDR) genes and genes conferring hypervirulence are organized by ST-type. *ST528 strain KLEB3 typed as ST716 by MLST CGE typer.

Currently, the collection includes 74 *Klebsiella* strains, of which 69 are clinical isolates from different countries (Supplementary Table S1), while the remaining five are ATCC/NCTC-type strains. These 74 strains represent six *Klebsiella* species, 41 known sequence types (STs), 32 capsule locus (KL) types, and 11 O-antigen (O) types (Fig. 1E and Supplementary Table S1). The most prevalent ST-types in KlebPhaCol include clinically relevant types associated with AMR, ST258 (*n* = 8 strains), ST14 (*n* = 8), ST11 (*n* = 6), ST101 (*n* = 5), and ST15 (*n* = 4) (Fig. 1E). Regarding KL-type, KL2 is the most prevalent in the collection (*n* = 10 strains) and is highly clinically relevant due to its strong association with virulence traits [127, 128]. The other notoriously pathogenic KL-type, KL1, is covered by two of our strains [128–130]. Other common KL-types include KL24 (*n* = 6), KL106 (*n* = 5), and KL17 (*n* = 5) (Fig. 1E). Lastly, KlebPhaCol strains represent 10 of the 13 known O-antigens for *Klebsiella* [131] (Supplementary Table S1). One strain also has OL103, a currently unclassified O-antigen. The most represented O-antigen is O1ab (*n* = 23), followed by O2afg, O2a, and O3b (*n* = 10 for each). OL101 recently classified as a 13th class of O-antigen (O13) is found in four strains [131]. The strains were also characterized in terms of prophage, virulent factors, stress resistance, AMR, anti-phage defence systems, and capsular locus integrity. Regarding virulence, we identified 44 virulence genes, with an average of 14 ± 6 per strain (Fig. 2A). The most common virulence genes were *entB, ompA, fepC, ykgk*, and genes from the *yag* cluster (Supplementary Table S1), which contribute to enterobactin siderophore production [132], host immune evasion [133], and biofilm formation [134]. Stress resistance genes were prevalent in the KlebPhaCol collection, with strains encoding an average of 17 ± 6 genes (Fig. 2A). The most frequently found gene was *fieF*, present in 70 out of 74 strains, responsible for iron and zinc efflux [135]. Genomic analysis revealed the presence of genes potentially mediating resistance to 22 antibiotics, including 6 aminoglycosides, 2 amphenicols, and various others (Fig. 2A). On average, strains had resistance genes for 8 ± 4 antibiotics. High carriage of genes associated with resistance was observed for phenicols, quinolones, β-lactams (in general, including cephalosporins), and trimethoprim (Fig. 2A). These analyses do not necessarily predict phenotypic resistance, with the possibility of resistance being mediated by genes operating in a multifactorial manner and intrinsic resistance associated with poor cell penetration and/or efflux. Therefore, experimental validation of these predictions was carried out for a defined selection of clinically important antibiotics using MIC. Carbapenem resistance predictions were 100% accurate, but resistance to gentamicin and tobramycin was higher in laboratory conditions than predicted (28 versus 23 strains and 31 versus 22 strains, respectively), and amikacin resistance was slightly lower than anticipated (23 versus 25 strains, Fig. 2A and Supplementary Table S1). This demonstrates the difference between genotypic resistance predictions and phenotypic susceptibility determination. Finally, the strains in this collection encode a total of 93 distinct defence systems, with an average of 11 ± 4 systems per strain (Supplementary Fig. S1). Most systems were rare, with 54 out of 93 systems present in fewer than five strains. Only RM type IV and AbiE systems were found in ~85% of the strains. Other notable defence systems included Mok Hok Sok, RM types I and II, and SoFic, present in ≥50% of the strains (Supplementary Fig. S1).

To facilitate access to the strains and their metadata and encourage comparisons to other *Klebsiella* strains, we have deposited the *K. pneumoniae* strains into a Pathogenwatch [95] collection (see Data availability).

### Roth phages infect up to 19 KL-types

**Figure 3.**
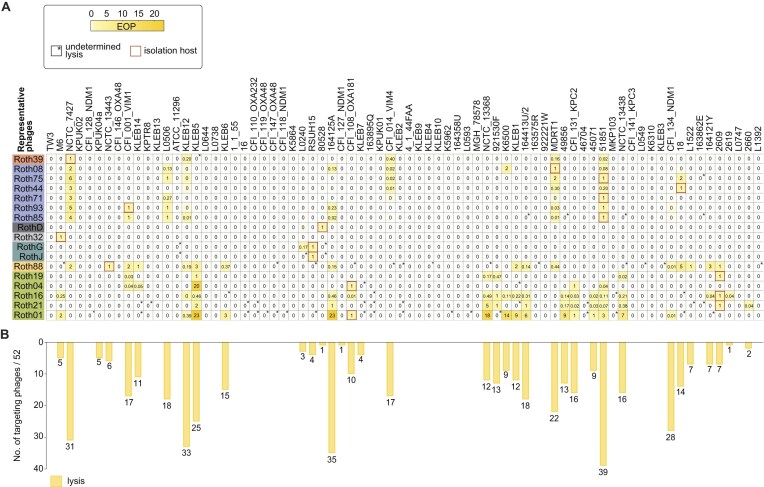
Klebsiella Phage Collection host range in LB broth. (**A**) The host range of representative phages (17/52 KlebPhaCol phages) against 74 strains in LB broth is shown as efficiency of plating (EOP) relative to the isolation strain (red boxes). An asterisk indicates undetermined lysis. (**B**) Quantification of each strain susceptibility to the complete collection of 52 KlebPhaCol phages.

The Roth phages demonstrated a broad ability to infect a wide range of *Klebsiella* strains, with notable success across multiple ST, KL, and O-antigen types (Fig. 3A and Supplementary Fig. S2). Among the 74 strains tested, 36 (49%) were susceptible to the Roth phages, including 20/42 (48%) ST-types, 19/32 (59%) KL-types, and 7/11 (64%) O-antigen types. Among the representative phages shown in Fig. 3A, Roth16 from the *Jiaodavirus* genus was the most effective, infecting 18/74 strains (24%), including 12 ST-types, 12 KL-types, and 6 O-types (Fig. 3A and Supplementary Fig. S2). *Slopekvirus* Roth88 was the second most effective phage, infecting 16/74 (22%) strains (Fig. 3A), including 11 ST-types, 11 KL-types, and 7 O-types (Supplementary Fig. S2). *Slopekvirus* exhibited notable success against KL2 strains, infecting up to half of the strains tested (5/10). Interestingly, ST258 strains are resistant to *Slopekvirus*, but efficiently targeted by all *Jiaodavirus, Sugarlandvirus*, and *Webervirus*.

Roth phages were able to infect strains associated with seven of the ten known O-antigen types in the KlebPhaCol collection, except for O2ac, O12, O13, and OL13 (Supplementary Fig. S2). *Slopekvirus* were most successful against strains with O-antigen O1ab, while *Jiaodaviruses* excelled at targeting O2afg.

Whereas *K. pneumoniae* is the most pathogenic species among *Klebsiella spp*., other species are emerging with serious pathogenic concerns [136, 137]. KlebPhaCol phages demonstrated lytic activity beyond *K. pneumoniae*, including *K. oxytoca* (164413U/2, KLEB11), *K. quasipneumoniae* (NCTC_13 368), and *K. variicola* (921530F) (Fig. 3). Together, these findings indicate that the KlebPhaCol collection could be expanded to study interactions across a broader range of *Klebsiella* species.

### Capsule-independent phages dominate the KlebPhaCol collection

**Figure 4.**
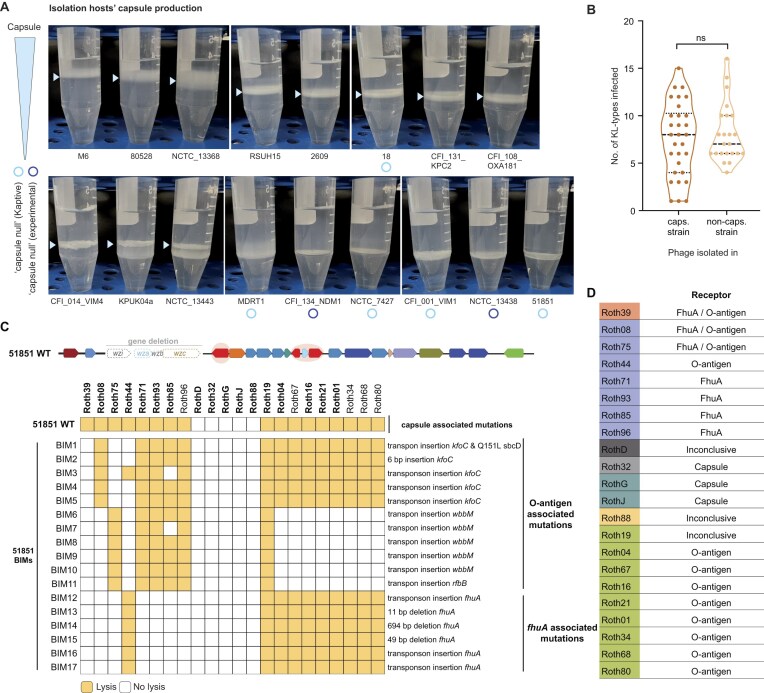
Capsule quantification and KlebPhaCol phage receptors. (**A**) Capsule quantification of 17 *K. pneumoniae* strains based on Percoll gradient centrifugation. Capsule layer(s) are highlighted with blue arrows. (**B**) Quantification of the number of targeted KL-types for phages isolated in capsulated strains (caps.) and non-capsulated strains (non-caps.). Statistical significance was assessed using an unpaired *t*-test (ns, non-significant, *P* = 0.2617). (**C**) Infectivity of the selected phages against the WT capsule-deficient strain 51851 and its BIM clones. (**D**) Interpretation of results from (C) indicating likely receptors for the representative phages. Roth32, RothG, and RothJ likely target capsule due to the presence of depolymerases in their genomes.

For most reported *Klebsiella* phages, the capsule is the primary surface receptor to which they attach [32, 34, 40, 46], although other surface receptors like O-antigen and lipopolysaccharide (LPS) have also been shown to serve as primary receptors for some *Klebsiella* phages [33, 34, 138]. Therefore, phage host range in *Klebsiella* is largely dictated by the presence or absence of the capsular polysaccharide [33, 34]. We examined the capsule composition of the 17 strains used for phage isolation, using a combination of genomic and experimental approaches. Specifically, we analysed the capsule locus architecture (Supplementary Fig. S3), performed capsule typing with Kaptive [93] (Supplementary Fig. S3 and  Supplementary Table S1), and assessed capsule-associated density phenotypes using Percoll gradient centrifugation [104] (Fig. 4A). Based on these analyses, five of the isolation strains are likely capsule-null (CFI_134_NDMI, NCTC_7427, CFI_001_VIM1, NCTC_13 438, and 51851), with an additional strain (MDRT1) showing a low-capsule phenotype (Fig. 4A). The remaining strains either had intact capsule loci, lacked evidence of disrupted mutations or showed high buoyancy in the Percoll assay consistent with capsule production.

The strains with disrupted capsule production were used for the isolation of 23 of the 53 KlebPhaCol phages (Supplementary Table S2). Interestingly, these phages are not capable of infecting a significantly broader range of capsular types compared to phages isolated from capsulated strains (Fig. 4B). This observation suggests that using capsule-deficient strains did not inherently select for phages with broader capsular tropism, and therefore the broader host range of the KlebPhaCol phages is not simply due to the use of capsule-deficient hosts for phage isolation.

Since most phages display a broad host range, we hypothesised that they do not rely on the capsule as their primary receptor. Exceptions are phages Roth32 (*Gajwadongvirus*), and RothG, RothI, and RothJ (*Drulisvirus*), which exhibited narrow host ranges (1–2 KL-types) and were isolated on strains with intact capsule loci (80528, M6, and RSUH15; Figs 3 and 4A). Together with evidence that these phages encode capsule depolymerases (Supplementary Text), this suggests they likely target the capsule as their primary receptor.

For the remaining phage families, we further analysed BIMs that emerged after phage exposure on the capsule-deficient strain 51851. Culturing and sequencing these BIMs, followed by re-testing phage infectivity, revealed that KlebPhaCol phages from the *Sugarlanvirus, Webervirus, Slopekvirus*, and *Jiaodavirus* families use either the LPS O-antigen, the outer membrane protein FhuA, or both as receptors (Fig. 4C and D), confirming they are capsule-independent. Interestingly, these phages can still infect capsulated strains, suggesting that the capsule does not substantially interfere with their access to surface receptors.

### KlebPhaCol phages encompass 14 different RBP structural clusters

**Figure 5.**
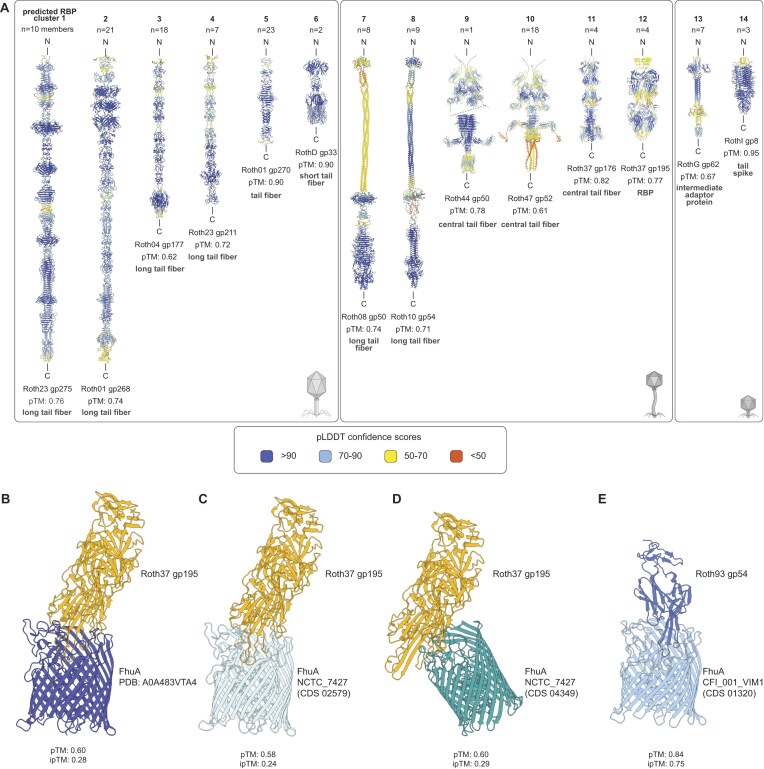
Structural predictions of KlebPhaCol RBPs. (**A**) Most (*n* = 135) RBPs predicted by RBPdetect, cluster into 14 groups following structural predictions and clustering with AlphaFold2 and Foldseek, respectively. Structures are coloured by pLDDT scores. (**B–D**) FhuA interactions with RBP gp195 from Roth37 (cluster 12; *Sugarlandvirus*). (**E**) FhuA interactions with RBP gp54 from Roth93 (*Webervirus*).

To further investigate receptor–phage interactions, we analysed the 207 predicted RBPs of the phages (Supplementary Table S3). Structural modelling using AlphaFold2 [139] followed by clustering with Foldseek [77] revealed that the predicted RBPs are highly diverse, forming 14 distinct clusters ranging from singletons to groups with up to 23 members (Fig. 5A). The myo- and siphophages each had six different clusters of RBPs, whereas podophages represented two of the clusters. The predicted structures included long (Roth23 gp275, Roth01 gp268, Roth08 gp50, Roth10 gp54, Roth04 gp177, and Roth23 gp11) and short tail fibres (RothD gp33), tail spikes (RothI gp8), central tail fibres (Roth44 gp50, Roth47 gp52, and Roth37 gp176), and other unclassified RBP structures (Roth37 gp195 and RothG gp62) (Fig. 5A). Interestingly, three clusters (2, 7, and 8) included members with domains at the tip of tail fibres that are structurally similar to intramolecular chaperones [140] that undergo auto-proteolytic cleavage after aiding in protein folding. Cluster 6 consists of a single tail fibre protein (RothD gp33) that has a rare polyglycine rich domain consisting of a conserved sandwich fold with hypervariable loops known to target both protein and LPS targets [141]. The proteins in cluster 12 showed high structural similarity to the central tail fibre protein pb5 of phage T5, which is known to bind to FhuA [142]. Therefore, we sought to predict the interaction complex of Roth37 gp195 with FhuA (PDB: A0A483VTA4; Fig. 5B), which had little deviation from the pb5–FhuA complex (RMSD = 0.8Å; PDB: 8B14). As Roth37 was isolated against non-capsulated strain NCTC_7427, we also predicted its RBP (gp195) interaction to both NCTC_7427-encoded FhuAs with similar interaction scores (Fig. 5C and D).

As our BIM data showed that several weberviruses (except Roth44) were sensitive to FhuA-associated mutations (Supplementary Fig. S3), we also searched for *Webervirus* protein(s) interacting with FhuA, in addition to those predicted by RBPdetect [73]. Comparisons of different *Webervirus* representatives revealed two proteins with interacting potential, gp53 and gp54 in Roth93, which are not present in the insensitive phage, Roth44. Structural prediction with FhuA from Roth93’s isolation strain showed that only gp54 was able to form a reliably predicted complex with this protein (Fig. 5E) (FhuA-Roth93_gp53 pTM = 0.83, iPTM = 0.45; FhuA-Roth93_gp54 pTM = 0.84, iPTM = 0.75).

### Phage infectivity is influenced by bacterial growth media

The availability of surface receptors on the bacterial surface is strongly influenced by media composition [143], and as a result can affect phage infectivity. To assess possible influences of media in phage host range, we performed additional host range assays in TSB, a medium that is commonly used in *Klebsiella* research and with different nutritional composition to LB [33, 144].

**Figure 6.**
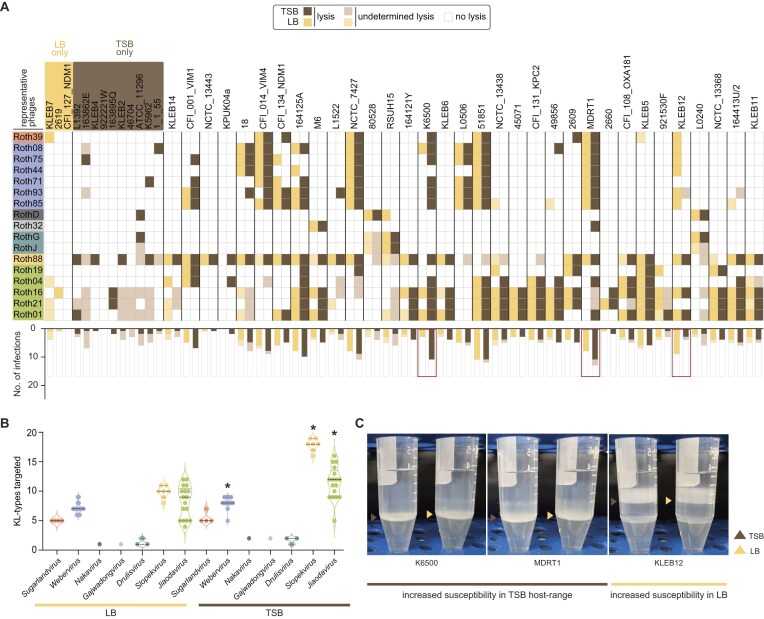
Host range of representative phages in TSB versus LB media. (**A**) The heatmap represents lysis, undetermined lysis, and no lysis in a binary mode for each media type (see Supplementary Table S4 for complete host range), which is tallied up at the bottom. Markedly differential strains between the two media types are boxed in red. Only strains susceptible to at least one phage of the collection are shown (*n* = 43/74). (**B**) KL-type infectivity per phage genus in TSB versus LB. *Statistical significances (*P* < 0.05) were calculated for all genera (except *Nakavirus* and *Gajwadongvirus*, n = 1) with a non-parametric paired *t*-test. (**C**) Capsule quantification of strains with marked differences in phage susceptibility in TSB versus LB. Capsule layers in TSB and LB are highlighted with brown and yellow arrows, respectively.

The impact of media composition on phage infectivity was evident in the differential success rates observed between TSB and LB (Fig. 6A, Supplementary Fig. S4, Supplementary Table S4, and Supplementary Text). In TSB, 638 total phage infections were recorded, compared to 486 in LB, indicating higher infectivity overall in TSB. The media-specific differences were particularly pronounced for certain phages. For instance, *Slopekvirus* phages Roth88, Roth26, and Roth27 infected up to 25 strains in TSB; but in LB, Roth88 and Roth26 infected only 16 strains while Roth27 infected only 12. Moreover, some strains were only infected in one of the two media. For example, CFI_127_NDM1, 2619, and KLEB7 were only infected in LB by 1–4 phages, while strains K5962, ATCC_11 296, 46 704, KLEB2, 163895Q, 922221W, KLEB4, 163862E, and L1392 were exclusively infected in TSB (Figs 3B and 6A, and Supplementary Fig. S4).

Interestingly, phage infectivity varied by genus in response to media composition (Fig. 6B). *Jiaodavirus* and *Slopekvirus* showed higher success rates in TSB, with ∼1.2× and 1.6× more infections recorded in this medium, respectively. For example, *Slopekvirus* Roth26 and Roth27 can infect up to 19 different KL-types in TSB, whereas in LB they infect 9 and 10 different KL-types, respectively (Fig. 6B and Supplementary Fig. S2). Furthermore, analysis revealed that capsule production in the three strains (K6500, MDRT11, and KLEB12) with the greatest medium-dependent differences in phage susceptibility was similar (Fig. 6C), suggesting that changes in phage host range between media are not attributable to capsule variation alone, but may also involve differences in the expression of other surface receptors and/or anti-phage defence systems.

### Abundance of defence systems does not correlate with phage susceptibility

Anti-phage defence systems pose a barrier to phages once inside the cell [145–147]. We observed that some of the least susceptible strains like L1522 and L0738 harboured a disproportionately high number of defence systems compared to the rest of the strains (22 and 31, respectively, Supplementary Fig. S1A). To investigate whether phage susceptibility was associated with the number of defence systems in each strain, we calculated and visualised Spearman rank correlations between the number of defence systems and various infection outcomes, including productive infections, no infections, and undetermined infections (i.e. “lysis from without”) (Supplementary Fig. S1B). This analysis revealed that in the panel of phage and clinical isolates tested, there was no significant correlation between the number of encoded defence systems and phage susceptibility, regardless of the media type. The recently reported PhageHostLearn model for *K. pneumoniae* phages suggests that RBP variability accounts for most of the host spectrum diversity [148], hinting at a lesser role of phage defence systems in shaping host range. However, conflicting findings have been reported for non-capsulated species [149], and further investigation is needed. Specifically, future analysis of phage adsorption to strains without productive infection may provide new insights and uncover correlations not evident with the current dataset.

Additionally, we predicted the putative anti-defence proteins harboured by the Roth phages to explore whether these might influence phage infectivity. We identified three putative anti-defence genes in the *Jiaodavirus* and *Slopekvirus* phages (Supplementary Table S2). In *Jiaodavirus*, these included two anti-CBASS and one anti-TA, whereas the *Slopekvirus* encoded two anti-CBASS and one anti-RM proteins. We also identified an anti-RM gene in a subset of *Webervirus* phages, specifically those branching from the second clade within the *Webervirus* group (Fig. 1A).

### Phage activity against gut-associated *K. pneumoniae* under aerobic and anaerobic conditions

**Figure 7.**
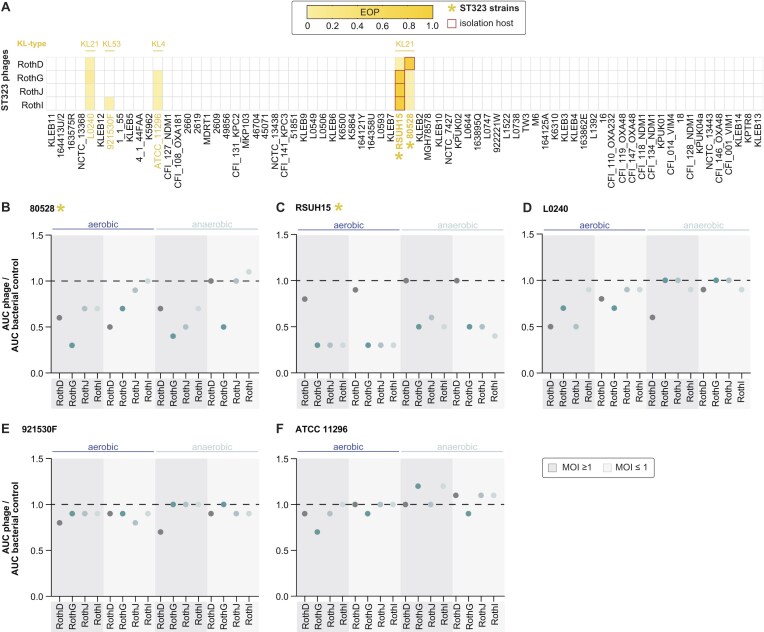
Infectivity of ST323-targeting Roth phages in aerobic and anaerobic conditions. (**A**) Heatmap of the infectivity patterns of the ST323-targeting Roth phages in LB medium represented as EOP compared to isolation strains (red box). The capsule locus (KL) type of susceptible strains is written above, and ST323 strains are marked with an asterisk. (**B–F**) Growth curves of the ST323-targeting phages against the susceptible strains in aerobic and anaerobic conditions, tested using two multiplicities of infection (MOI ≥ 1 and ≤ 1), and represented as AUC ratio compared to the uninfected bacterial control.

Certain *K. pneumoniae* sequence types are commonly associated with specific host or disease contexts. For example, ST323 has been linked to enrichment in the gut microbiota of patients with IBD and shown to exacerbate inflammation in a mouse model [21]. Although the ST323 strains used in our study were not isolated from IBD patients, we aimed to assess whether KlebPhaCol includes phages capable to targeting this gut-associated lineage. Four phages—RothD, RothG, RothJ, and RothI—were found to infect two ST323 strains (RSUH15 and 80528, both KL21) as well as three non-ST323 strains (ST91, ST635, and ST1875) (Fig. 7A).

Given the relevance of oxygen availability in the gut environment, we evaluated the ability of these phages to inhibit bacterial growth under aerobic and anaerobic conditions. Liquid infection assays were performed in both conditions, at two MOIs, and phage efficacy was quantified by comparing the area under the growth curve (AUC) relative to the no-phage control (Fig. 7B, and Supplementary Fig. S5 and Supplementary Table S5). As expected, bacterial growth was limited under anaerobic conditions. Nonetheless, several Roth phages remained active: RothG, RothJ, and RothI retained activity against RSUH15, albeit at reduced levels, while all four phages inhibited strain 80528 at high MOI, with RothG showing activity even at lower MOI (Fig. 7B and C). Among the non-ST323 strains (L0240, 921530F, and ATCC_11 296), RothD showed the highest efficacy in anaerobic conditions, particularly against L0240 and 921530F, although none of the phages were active against strain ATCC_11 296 in anaerobic conditions (Fig. 7D–F). Overall, these findings suggest that some KlebPhaCol phages retain functional activity in gut-relevant, low-oxygen conditions, an important consideration for future therapeutic applications.

 

*Felixviridae* are found in human gut metagenomes

 

#### Proposed new taxonomy for phage RothD

While most Roth phages could be classified into existing viral taxa, RothD could not be assigned to any existing viral family using standard classification tools like PhageGCN and vContact2. Therefore, we propose the establishment of a new family, *Felixviridae* (40–60 kb), and genus, *Nakavirus*, to accommodate these phages (see Supplementary Text).

Although RothD shares little overall similarity with its relatives, it encompasses a highly conserved region from gp1 to gp24 (1–20, 241 bp). Genomic synteny analysis of the proposed *Felixviridae* family revealed that the conserved region spanning the first 20,241 bp is shared across all members. Additional annotation by Phold, and comparison of the predicted structures with the PDB database, demonstrated that this core region is mostly composed of structural proteins (Supplementary Fig. S6). Most core proteins were associated with the phage capsule, neck, tube, or baseplate. The structural similarities to another myophage assembly [150] allowed for confident functional assignments of these proteins (i.e. major head protein, head-to-tail connector, neck collar protein, tail-sheath initiator, tail tube protein, and baseplate components). Interestingly, the two gene products gp9 and gp15 were annotated as hypothetical proteins by Phold and no similarities to functionally annotated proteins could be found with Foldseek, but homologs (80% and 67.3% sequence identity) were present in bacterial species associated with the human gut (i.e. *Citrobacter spp*. and *Serratia marcescens*, respectively). Outside this core region, gene conservation was minimal, further emphasising the uniqueness of these phages.

 

#### *Nakavirus* phages of the *Felixviridae* family are associated with *Enterobacteriaceae*

Most *Felixviridae* phages have a predicted temperate lifestyle, and thus we wanted to assess the prevalence of the corresponding prophages in bacterial genomes. We analysed 64,364 complete bacterial genomes from the Bacterial and Viral Bioinformatics Resource Center (BV-BRC) database for homologues of RothD and found that all matches (*n* = 7605, Supplementary Table S6) were exclusive to the *Enterobacteriaceae* family.

*Klebsiella* species were the most common hosts (566/708; 80%), spanning eight species and 111 ST-types, with ST231 appearing most frequently (127 hits). The second most represented species was *Salmonella enterica* (66 hits, 9%). These phage–host associations were found across 67 countries, indicating a widespread global presence of these prophages. Host metadata revealed that most isolates (521) are derived from humans, although samples from other hosts including chickens (10), sea lions (10), pigs (7), birds (4), sheep (3), cattle (2), hedgehogs (2), and even termites (4), suggest a broader superhost range (Supplementary Table S6). However, the location of the isolate within these other organisms is unknown.

We also examined predicted prophages within these genomes to determine whether our identified hits were located within the respective genomic regions. Of these hits, only two were entirely within prophage regions. The majority were either outside predicted prophages (*n* = 431/708, 61%) or partially within one (*n* = 266/708, 36%). To further investigate the gene neighbourhood of hits outside prophage regions, we extracted the ten upstream and downstream genes from each hit. In 259/431 (60%) of the cases, a lysozyme gene was found within 10 CDS upstream of the hit region. Additionally, in seven of these 259 cases, an integrase gene was also identified within 10 CDS downstream of the hit region. These observations suggests that some hits may indeed reside within prophage regions that are not detected by the prophage identifier tool, possibly, because many of the felixvirus prophages could be deteriorating, highlighting the importance of examining gene neighbourhoods for more comprehensive analysis.

 

#### *Felixviridae* phages are found in the gut

The taxonomic characterisation of the *Felixviridae* RothD, suggests that *Felixviridae* phages are present in the mammalian gut. Several groups of gut-related phages have been established, including the orders *Crassvirales* [151], as well as the familis *Flandersviridae* and *Quimbyviridae* [152], and the still unclassified Gubaphages [153]. The order *Crassvirales* includes the most abundant phages identified in the mammalian gut to date [151, 154]. Crassviruses have been shown to persist overtime, potentially via several specific adaptations to the gut environment [155, 156]. To determine the abundance of *Felixviridae* phages in the human gut, we examined their prevalence in the Gut Phage Database (GPD). We found 355 high-quality unique hits across 38 isolates and 317 metagenome-assembled genomes, corresponding to 0.86% of the high-quality phage genomes in the GPD (Supplementary Table S7). These phages appear globally widespread, with metagenomic samples collected from 15 different countries (Supplementary Table S7). Consistent with the analysis of the BV-BRC database above, analysis of GPD metadata confirmed that these phages are restricted to hosts of the *Enterobacteriaceae* family, with *Klebsiella spp*. being the most common (172/186 hits with available host-predicted data).

*Felixviridae*-related sequences were predominant in infant and adult cohorts, representing 82% of hits where data was available (Supplementary Table S8) and spanned both healthy and disease-associated microbiomes, highlighting their prevalence across ages and health statuses. In several (554/1345) metagenomes, multiple *Felixviridae*-related phages were present, suggesting that individuals could harbour diverse populations of these phages.

When examining disease associations, *Felixviridae* appeared in cohorts with chronic conditions like obesity, IBD, and rheumatoid arthritis (Supplementary Table S9) [105]. However, no significant associations were found, suggesting these phages persist in various gut environments without clear links to disease states.

 

#### *Felixviridae* reside in the gut in both free phage and prophage form

**Figure 8.**
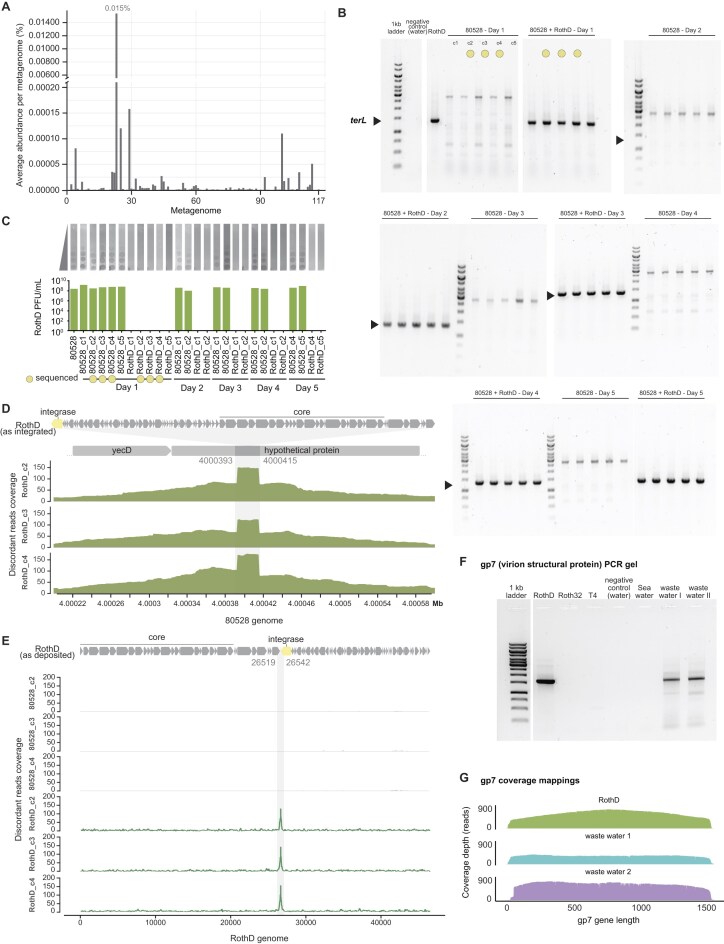
*Felixviridae* detection. (**A**) Abundance per metagenome of *Felixviridae-*like phages from 117 healthy human metagenomes. (**B**) PCR detection of *Felixviridae terL* gene in colonies from cultures of strain 80528 exposed to RothD over five consecutive days. Yellow circles indicate colonies sent for Illumina sequencing to confirm lysogen presence. (**C**) Plaque forming units (PFU)/ mL of RothD spotted against selected colonies from (B) to confirm lysogen presence by the lack of infection. (**D**) Discordant read mappings to 80528 wildtype genome shows the region of insertion (represented by increased coverage) was the same in all three sequenced colonies. (**E**) Discordant read mappings to the RothD genome shows the site of lysogen insertion was the same in all three sequenced colonies, a few base pairs before the integrase gene. (**F**) Primers to the gp7 (putative virion structural protein) of the core region of RothD can detect related phages in environmental samples by PCR. (**G**) Positive PCR products from (F) sequenced by long-read sequencing and displayed as coverage depth when mapped to the gp7.

To assess the abundance of *Felixviridae* phages in the human gut, we analysed 117 healthy stool samples from the Human Microbiome Project [157]. This analysis detected *Felixviridae* sequences in 89% of the samples (average $ \pm $ SD abundance: 0.00014% $ \pm $ 0.005%). These phages constituted a minor but consistent fraction of the gut microbiome (Fig. 8A). After removing bacterial reads, *Felixviridae* phages remained detectable in only 14% of samples, suggesting that they mainly reside as prophages in the gut.

To extend this analysis, we examined 826 additional human gut metagenomes from previous studies [112–115]. After quality control, host read removal and read mapping against a curated and dereplicated genome dataset, only 7 samples (∼1%) exhibited ≥ 50% phage genome coverage (Supplementary Table S10), representing an average $ \pm $ SD abundance per metagenome of 0.015% $ \pm $ 0.006%. All 54 *Felixviridae* phages were represented in this dataset except for Pantoea phage PdC23 (NC_071008.1) and the most abundant phage genome was GPD phage uvig_371 030 at 20.9% average relative abundance. Although lower identity or partial matches were found in additional samples, genome-wide ANI analysis showed these did not meet thresholds for true presence (≥90% ANI, ≥50% coverage). Nevertheless, retaining these partial matches maintained a similar average abundance per metagenome to that of the ‘true’ matches (average $ \pm \ $SD: 0.016% $ \pm $ 0.231%). Additionally, all of the detected phages encode lysogeny-associated genes (i.e. integrases or repressors), indicative of a temperate lifestyle and no high-confidence lytic *Felixviridae-*like signatures were observed. These phages were only detected in the metagenomic portion of paired metagenome-metavirome datasets, further supporting their temperate lifestyle (Supplementary Table S10). These findings suggest that *Felixviridae* are present in the human gut microbiome at low prevalence and exist primarily as integrated prophages.

Given the strong evidence of lysogeny for these phages, we sought to investigate if RothD could actively lysogenise the host. After five consecutive days of exposing the isolating host, 80528, to RothD at an MOI of 1, we analysed bacterial colonies recovered from each day for the presence of the *terL* gene via PCR. We were able to detect *terL* in all colonies from day 1 (Fig. 8B), suggesting RothD was able to integrate into 80528. To further confirm integration, we spotted RothD against these colonies, showing no infection by RothD, suggesting superinfection exclusion due to the integrated prophage (Fig. 8C). Additionally, we sent three colonies of each sample from day 1 for sequencing. Mapping of the sequencing reads to the host strain 80528, revealed that RothD always integrates at the same site within a 33 bp region in 80528. This site was located within the genomic positions 4,000,393–4,000,415 bp inside the coding open reading frame of a hypothetical protein (Fig. 8D; full reads coverage in Supplementary Fig. 7A). Additionally, mapping of reads to RothD revealed that the corresponding attachment site in the phage genome is located upstream of the integrase gene (gp34) at positions 26,519–26,542 bp (Fig. 8E; full reads coverage in Supplementary Fig. 7B). This integration structure is conserved in the two most closely related prophages (IMGVR_UViG_2571042619_000002 and IMGVR_UViG_2588254063_000001; Supplementary Text Fig. 7E), whereby the same integrase gene is inserted as the first gene of their lysogen versions in a *Klebsiella* host, and the last gene is a homolog of RothD_gp33 (Supplementary Fig. 7C). Additionally, read coverage analysis demonstrated that there is an integration rate of ∼40%, indicating that in these experiments ∼60% of virions remain as ‘free’, non-integrated phage (see methods).

 

#### *Felixviridae* are detectable in the environment

To assess the feasibility of PCR detection of *Felixviridae* in environmental samples, we designed primers against the conserved predicted virion structural protein gp7 of the *Felixviridae* family. We screened for these phages in environmental samples collected from a wastewater facility in Portswood and Petersfield, and from the sea of the Isle of Wight (all UK-based). *Felixviridae* were present in the two sampled wastewater facilities but not in the sea water sample (Fig. 8F), which we confirmed by long-read sequencing of the PCR products (Fig. 8G). These preliminary results are consistent with a link between *Felixviridae* and human-associated environments, and demonstrate that PCR-based detection of these phages in environmental samples is feasible.

Together, these data suggest *Felixviridae*, particularly *Nakavirus*, are widely distributed across diverse human and animal hosts, persisting throughout life stages and health conditions. This broad host range, coupled with environmental presence, positions *Felixviridae* as integral, even if relatively minor, yet understudied components of the gut virome, meriting further exploration for their ecological and potential clinical impacts.

## Discussion

Here we introduce KlebPhaCol, an open-source collection containing 52 phages targeting *Klebsiella spp*., covering seven genera, and 74 *Klebsiella* strains spanning 41 known ST-types and 32 K-types. By offering a centralised, no-cost, and well-documented collection, KlebPhaCol democratises access to essential resources for researchers worldwide.

KlebPhaCol includes strains from six different species of the *Klebsiella* genus including members of the *K. pneumoniae* species complex (KpSC), *K. variicola and K. quasipneumoniae*, that are emerging as public health concerns [136, 137]. Because *K. pneumoniae* is the most pathogenic and prevalent species of this genus [158], the collection includes 65 different isolates of this species. Although most isolates in the collection originate from Europe, they represent some of the most antimicrobial resistant clones circulating globally, including ST258, ST11, ST14 and ST15 [12, 19, 159]. Asymptomatic gut colonization of *K. pneumoniae* has been demonstrated to increase the susceptibility to subsequent infections in other tissues [160–162], and high abundance of *K. pneumoniae* has been linked with IBD exacerbation [21]. A particularly relevant ST type in this context is ST323, which is also present in the collection [21, 163]. With this in mind, KlebPhaCol phages were isolated using enrichment cultures of antimicrobial-resistant relevant clones and gut-relevant clone ST323. The resulting host range revealed high specificity of such clinically-relevant clones, positioning the collection – both phages and bacterial strains – for studying these interactions in classical tissue infections as well as in gut colonisation contexts that at present remain largely unexplored [164].

Although KlebPhaCol spans six of the 13 currently recognised *Klebsiella* phage families, several known families remain underrepresented or absent. This likely reflects methodological biases during phage isolation, including the use of standard laboratory media (e.g. LB), selective targeting of clinical ST-types, and the reliance on sewage as the main environmental source. Similar limitations have been noted in other phage studies [165, 166]. To improve phage diversity in future iterations of KlebPhaCol, we plan to broaden the range of host strains and vary isolation conditions, as well as continue to incorporate phages from external contributors. This amendment will help capture a more complete representation of the *Klebsiella* phageome and facilitate broader applications across ecological and clinical contexts.

Genomic analyses of the phages in KlebPhaCol revealed high intergenomic similarities among several phages. It has been shown that even small polymorphisms within phages can result in differences worth investigating, including differences in host-range. For instance, despite phages T2, T4 and T6 being highly similar, these phages have different DNA hypermodifications and different extents of genomic DNA modification [167], and can bind to different receptors [168]. Moreover, characterisation of other *Klebsiella* phages sharing high similarity (>97%) demonstrated small differences in behaviour that could be attributed to differences in their L-shaped tail fibres [169]. KlebPhaCol includes 17 representative phages, for most of which closely related phages are available to investigate nuances. The importance of studying a representative collection of phages is exemplified by the advances that followed the establishment of the T phages [170–173]. Although the remarkable diversity of phages is increasingly being explored, focusing on a representative set of phage-bacteria interactions could, in a therapeutic context, offer a more tractable path toward a deeper understanding of phage biology and practical application.

To support such efforts, we expanded our analysis of RBPs and identified 14 distinct structural clusters among the KlebPhaCol phages. These clusters include both predicted capsule depolymerases and alternative adhesin-like proteins, which could reflect differences in receptor usage. We also tested spontaneous *Klebsiella* mutants across all 17 representative phages, revealing patterns of receptor dependence consistent with RBP diversity. Together, these findings underscore the utility of the collection for dissecting structure-function relationships in phage infection.

Traditionally within the *Klebsiella* field, phage therapy has mainly been considered as an alternative treatment for tissue infections. However, given the relevance of *Klebsiella* as a gut colonising pathobiont and a driver of subsequent infections and disease, considering phage therapy applications for gut microbiome modulation is becoming more relevant [21, 174]. In this study we show how phage efficiency can differ when environmental conditions of testing are changed (e.g. media type, oxygen supply). Our results suggest that tailoring phage therapy to specific infection environments by taking into account nutrient availability and other site-relevant physiological conditions, could improve phage efficacy. Additionally, consistent with previous studies, we show that non-capsule targeting phages can have a broader host range [33, 34, 175]. This observation suggests that non-capsule targeting phages could offer versatile treatment options, especially when capsule types vary widely among infections. Indeed, *Klebsiella* phages encoding multiple depolymerases may also achieve a similar outcome [32], by being able to target multiple capsule types, albeit limited by the presence of these receptors in the bacterial strain. Multiple studies have shown that capsule expression and production, or lack thereof, result in fitness trade-offs for the bacterium [143, 164, 176]. Acapsular strains are substantially attenuated, due to the increased susceptibility to immune mechanisms [29, 177]. However, lack of capsule can facilitate biofilm formation [178, 179] and cell adhesion [180]. Different factors have been shown to drive capsule loss or maintenance including nutrient availability [143], oxygen availability [181] and insertion sequence repertoires [164]. Indeed, capsule loss in *Klebsiella* strains has been demonstrated upon gut colonisation [164] and in urinary tract infections [182]. Thus, both capsule and non-capsule targeting phages are relevant for therapeutic applications, and the preference for the former or the latter might depend on the targeted infection site, emphasising the need to better understand *Klebsiella* behaviour in different infection sites to improve phage selection for therapeutic purposes.

In this work, we identified a novel gut-associated phage family, *Felixviridae*, represented by KlebPhaCol phage RothD of the *Nakavirus* genus. *Felixviridae*-like phages are largely temperate phages with notable association to *Enterobacteriaceae* members, which include key human pathogens. With most of these being uncultured phages, we were only able to confirm the ability to lysogenise for RothD. *Felixviridae*-like phages are geographically widespread and present across human age groups, from pre-term infants to adults, and the presence of these phages in healthy gut microbiomes points to potential roles in the human gut virome. *Felixviridae* presence in the gut could simply reflect their host ecology particularly given their lysogenic nature. However, detection of homologs of RothD core proteins gp9 and gp15 (shared within *Felixviridae* phages) only in gut-associated bacterial genomes, suggests potential evolutionary gene acquisitions of this phage family to survive in this niche, requiring further investigation. Other *Klebsiella*-targeting phage groups, such as weberviruses, have been recently identified in the gut [183]. These findings underscore the importance of exploring the roles of various *Klebsiella* phage groups within the gut microbiome, now facilitated by the inclusion of *Nakavirus* RothD and several *Webervirus* in KlebPhaCol. PCR detection of gut-associated phages has been shown to be a cost-effective method with extensive applications, including evaluation of the prevalence of the most abundant human gut phage group, crAssphage, as proxy for human faecal contamination [184–186]. By establishing PCR detection protocols for *Felixviridae*, our findings lay the groundwork for future research on these phages, both in clinical and environmental contexts.

Altogether, KlebPhaCol provides an openly accessible resource for studying *Klebsiella* phage interactions. It offers a uniquely broad scope, spanning critical pathogenic strains, non-capsule-targeting phages with versatile applications, and a newly described gut phage family with potential implications for human health. In addition to these broader features, the collection offers granular experimental and bioinformatic insights, including defined RBP structural clusters, receptor usage patterns, and phenotypic differences among closely related phages, that can drive new mechanistic studies. Recognising the lack of standardised open biosharing regulations and pipelines, we actively participate in discussions to address this crucial need for research progress [187]. We expect that KlebPhaCol will not only facilitate new discoveries in microbiology and therapeutic research but also inspire contributions from the broader scientific community to further expand and improve this evolving resource.

## Supplementary Material

gkaf1122_Supplemental_Files

## Data Availability

Phage assemblies from this study are available at GenBank accessions PQ657785-PQ657835, PP934563 and PP934564. Phage raw reads are available at PRJNA1192413, and annotation files at Figshare (DOI: https://doi.org/10.6084/m9.figshare.27794211.v1). Bacteria assemblies can be found under BioProject accessions PRJNA1123654, PRJNA73191, PRJNA1121092, PRJNA1121093, PRJNA31, PRJNA745534, and PRJNA1187231. Bacteria raw reads and annotation files are available at Figshare (DOI: https://doi.org/10.6084/m9.figshare.27759627.v1). Raw reads of the BIMs from strain 51851 are available at Figshare (DOI: https://doi.org/10.6084/m9.figshare.29437796). The KlebPhaCol strain collection in Pathogenwatch can be accessed at https://pathogen.watch/collection/tdtgvdxs4xhw-klebphacol-kp-strains. All other supporting data of this study are available within the article or through the supplementary data files. Bacterial strains and phages from this study are freely available upon request through our website, www.klebphacol.org via a simple online form (subject to verification). Please note that shipping costs and any additional requirements will apply.

## References

[1] Hoffman SJ, Caleo GM, Daulaire N et al. Strategies for achieving global collective action on antimicrobial resistance. Bull World Health Organ. 2015;93:867. 10.2471/BLT.15.153171. 26668439 PMC4669731

[2] Landers T, Kavanagh KT. Is the Presidential Advisory Council on Combating Antibiotic Resistance missing opportunities?. American Journal of Infection Control. 2016;44:1356–9. 10.1016/j.ajic.2016.07.008. 27597393

[3] Lin DM, Koskella B, Lin HC. Phage therapy: an alternative to antibiotics in the age of multi-drug resistance. WJGPT. 2017;8:162. 10.4292/wjgpt.v8.i3.162. 28828194 PMC5547374

[4] Kortright KE, Chan BK, Koff JL et al. Phage Therapy: a Renewed Approach to Combat Antibiotic-Resistant Bacteria. Cell Host & Microbe. 2019;25:219–32. 10.1016/j.chom.2019.01.014. 30763536

[5] Luepke KH, Mohr JF. The antibiotic pipeline: reviving research and development and speeding drugs to market. Expert Review of Anti-infective Therapy. 2017;15:425–33. 10.1080/14787210.2017.1308251. 28306360

[6] Payne DJ, Miller LF, Findlay D et al. Time for a change: addressing R&D and commercialization challenges for antibacterials. Phil Trans R Soc B. 2015;370:1–12. 10.1098/rstb.2014.0086. PMC4424435 25918443

[7] Sulakvelidze A, Alavidze Z, Morris J. Bacteriophage Therapy. Antimicrob Agents Chemother. 2001;45:649. 10.1128/AAC.45.3.649-659.2001. 11181338 PMC90351

[8] Wittebole X, De Roock S, Opal SM. A historical overview of bacteriophage therapy as an alternative to antibiotics for the treatment of bacterial pathogens. Virulence. 2013;5:226–35. 10.4161/viru.25991. 23973944 PMC3916379

[9] Pires DP, Costa AR, Pinto G et al. Current challenges and future opportunities of phage therapy. FEMS Microbiol Rev. 2020;44:684–700. 10.1093/femsre/fuaa017. 32472938

[10] Pirnay JP, Djebara S, Steurs G et al. Personalized bacteriophage therapy outcomes for 100 consecutive cases: a multicentre, multinational, retrospective observational study. Nat Microbiol. 2024;9:1434–53. 10.1038/s41564-024-01705-x. 38834776 PMC11153159

[11] Pendleton JN, Gorman SP, Gilmore BF. Clinical relevance of the ESKAPE pathogens. Expert Review of Anti-infective Therapy. 2014;11:297–308. 10.1586/eri.13.12. 23458769

[12] Wyres KL, Holt KE. Klebsiella pneumoniae Population Genomics and Antimicrobial-Resistant Clones. Trends in Microbiology. 2016;24:944–56. 10.1016/j.tim.2016.09.007. 27742466

[13] Tacconelli E, Carrara E, Savoldi A et al. Discovery, research, and development of new antibiotics: the WHO priority list of antibiotic-resistant bacteria and tuberculosis. The Lancet Infectious Diseases. 2018;18:318–27. 10.1016/S1473-3099(17)30753-3. 29276051

[14] Ko WC, Paterson DL, Sagnimeni AJ et al. Community-Acquired Klebsiella pneumoniae Bacteremia: global Differences in Clinical Patterns. Emerg Infect Dis. 2002;8:160. 10.3201/eid0802.010025. 11897067 PMC2732457

[15] David S, Reuter S, Harris SR et al. Epidemic of carbapenem-resistant Klebsiella pneumoniae in Europe is driven by nosocomial spread. Nat Microbiol. 2019;4:1919–29. 10.1038/s41564-019-0492-8. 31358985 PMC7244338

[16] Shon AS, Bajwa RPS, Russo TA. Hypervirulent (hypermucoviscous) Klebsiella pneumoniae: a new and dangerous breed. Virulence. 2013;4:107. 10.4161/viru.22718. 23302790 PMC3654609

[17] Rupali P . Extremely high mortality rates in patients with carbapenem-resistant, hypermucoviscous Klebsiella pneumoniae blood stream infections. J Assoc Phys Ind. 2018;66:13. 31313543

[18] Choby JE, Howard-Anderson J, Weiss DS. Hypervirulent Klebsiella pneumoniae – clinical and molecular perspectives. J Intern Med. 2020;287:283–300. 10.1111/joim.13007. 31677303 PMC7057273

[19] Wyres KL, Lam MMC, Holt KE. Population genomics of Klebsiella pneumoniae. Nat Rev Microbiol. 2020;18:344–59. 10.1038/s41579-019-0315-1. 32055025

[20] Podschun R, Ullmann U. Klebsiella spp. as nosocomial pathogens: epidemiology, taxonomy, typing methods, and pathogenicity factors. Clin Microbiol Rev. 1998;11:589–603. 10.1128/CMR.11.4.589. 9767057 PMC88898

[21] Federici S, Kredo-Russo S, Valdés-Mas R et al. Targeted suppression of human IBD-associated gut microbiota commensals by phage consortia for treatment of intestinal inflammation. Cell. 2022;185:2879–98. 10.1016/j.cell.2022.07.003. 35931020

[22] Atarashi K, Suda W, Luo C et al. Ectopic colonization of oral bacteria in the intestine drives TH1 cell induction and inflammation. Science (1979). 2017;358:359–65. 10.1126/science.aan4526 PMC5682622 29051379

[23] Ichikawa M, Nakamoto N, Kredo-Russo S et al. Bacteriophage therapy against pathological Klebsiella pneumoniae ameliorates the course of primary sclerosing cholangitis. Nat Commun. 2023;14:1–13. 10.1038/s41467-023-39029-9. 37277351 PMC10241881

[24] Pan YJ, Lin TL, Chen CT et al. Genetic analysis of capsular polysaccharide synthesis gene clusters in 79 capsular types of Klebsiella spp. Sci Rep. 2015;5:1–10. 10.1038/srep15573. PMC4616057 26493302

[25] Lam MMC, Wick RR, Watts SC et al. A genomic surveillance framework and genotyping tool for Klebsiella pneumoniae and its related species complex. Nat Commun. 2021;12:1–16. 10.1038/s41467-021-24448-3. 34234121 PMC8263825

[26] Lam MMC, Wick RR, Judd LM et al. Kaptive 2.0: updated capsule and lipopolysaccharide locus typing for the Klebsiella pneumoniae species complex. Microb Genom. 2022;8:800. 10.1099/mgen.0.000800 PMC9176290 35311639

[27] Orskov I, Fife-Asbury MA. New Klebsiella capsular antigen, K82, and the deletion of five of those previously assigned. International Journal of Systematic Bacteriology. 1977;27:386–7. 10.1099/00207713-27-4-386.

[28] Lee IR, Molton JS, Wyres KL et al. Differential host susceptibility and bacterial virulence factors driving Klebsiella liver abscess in an ethnically diverse population. Sci Rep. 2016;6:1–12. 27406977 10.1038/srep29316 PMC4942785

[29] Huang X, Li X, An H et al. Capsule type defines the capability of Klebsiella pneumoniae in evading Kupffer cell capture in the liver. PLoS Pathog. 2022;18:e1010693. 10.1371/journal.ppat.1010693. 35914009 PMC9342791

[30] Rieger-Hug D, Stirm S. Comparative study of host capsule depolymerases associated with Klebsiella bacteriophages. Virology. 1981;113:363–78. 10.1016/0042-6822(81)90162-8. 7269247

[31] Niemann H, Kwiatkowski B, Westphal U et al. Klebsiella serotype 25 capsular polysaccharide: primary structure and depolymerization by a bacteriophage-borne glycanase. J Bacteriol. 1977;130:366–74. 853030 10.1128/jb.130.1.366-374.1977 PMC235214

[32] Pan YJ, Lin TL, Chen CC et al. Klebsiella Phage ΦK64-1 Encodes Multiple Depolymerases for Multiple Host Capsular Types. J Virol. 2017;91:16. 10.1128/JVI.02457-16. PMC5331798 28077636

[33] Lourenço M, Osbelt L, Passet V et al. Phages against noncapsulated Klebsiella pneumoniae: broader host range, slower resistance. Microbiol Spectr. 2023;11:18. 10.1128/spectrum.04812-22 PMC10433977 37338376

[34] Beamud B, García-González N, Gómez-Ortega M et al. Genetic determinants of host tropism in Klebsiella phages. Cell Reports. 2023;42:112048. 10.1016/j.celrep.2023.112048. 36753420 PMC9989827

[35] Tesson F, Hervé A, Mordret E et al. Systematic and quantitative view of the antiviral arsenal of prokaryotes. Nat Commun. 2022;13:1–10. 10.1038/s41467-022-30269-9. 35538097 PMC9090908

[36] Uskudar-Guclu A, Unlu S, Salih-Dogan H et al. Biological and genomic characteristics of three novel bacteriophages and a phage-plasmid of Klebsiella pneumoniae. Can J Microbiol. 2024;70:213–25. 10.1139/cjm-2023-0188. 38447122

[37] Alqurainy N, Miguel-Romero L, Moura de Sousa J et al. A widespread family of phage-inducible chromosomal islands only steals bacteriophage tails to spread in nature. Cell Host & Microbe. 2023;31:69–82. 10.1016/j.chom.2022.12.001. 36596306

[38] Zhao M, Li H, Gan D et al. Antibacterial effect of phage cocktails and phage-antibiotic synergy against pathogenic Klebsiella pneumoniae. Msystems. 2024;9:e00607–24. 10.1128/msystems.00607-24. 39166877 PMC11406915

[39] Ferriol-González C, Concha-Eloko R, Bernabéu-Gimeno M et al. Targeted phage hunting to specific Klebsiella pneumoniae clinical isolates is an efficient antibiotic resistance and infection control strategy. Microbiol Spectrum. 2024;12:e0025424. 10.1128/spectrum.00254-24. PMC11448410 39194291

[40] Townsend EM, Kelly L, Gannon L et al. Isolation and Characterization of Klebsiella Phages for Phage Therapy. PHAGE. 2021;2:26. 10.1089/phage.2020.0046. 33796863 PMC8006926

[41] Li M, Guo M, Chen L et al. Isolation and Characterization of Novel Lytic Bacteriophages Infecting Epidemic Carbapenem-Resistant Klebsiella pneumoniae Strains. Front Microbiol. 2020;11:522488. 10.3389/fmicb.2020.01554 PMC7385232 32793133

[42] Rotman E, McClure S, Glazier J et al. Rapid design of bacteriophage cocktails to suppress the burden and virulence of gut-resident carbapenem-resistant Klebsiella pneumoniae. Cell Host & Microbe. 2024;32:1988–2003. 10.1016/j.chom.2024.09.004. 39368473 PMC11563920

[43] Maffei E, Shaidullina A, Burkolter M et al. Systematic exploration of Escherichia coli phage–host interactions with the BASEL phage collection. PLoS Biol. 2021;19:e3001424. 10.1371/journal.pbio.3001424. 34784345 PMC8594841

[44] Humolli D, Piel D, Maffei E et al. Completing the BASEL phage collection to unlock hidden diversity for systematic exploration of phage–host interactions. PLoS Biol. 2025;23:e3003063. 10.1371/journal.pbio.3003063. 40193529 PMC11990801

[45] Brauer A, Rosendahl S, Kängsep A et al. Isolation and characterization of a phage collection against Pseudomonas putida. Environmental Microbiology. 2024;26:e16671. 10.1111/1462-2920.16671. 38863081 PMC7616413

[46] Bonilla E, Costa AR, Van Den Berg DF et al. Genomic characterization of four novel bacteriophages infecting the clinical pathogen Klebsiella pneumoniae. DNA Res. 2021;28:1–11. 10.1093/dnares/dsab013 PMC8386662 34390569

[47] Kropinski AM, Mazzocco A, Waddell TE et al. 2009; Enumeration of Bacteriophages by Double Agar Overlay Plaque Assay BT - Bacteriophages: methods and Protocols, Volume 1: isolation. Characterization, and Interactions. In Clokie M. R. J., Kropinski A. M. (eds). Humana Press, Totowa, NJ, pp.69–76. 10.1007/978-1-60327-164-6_7 19066811

[48] Ferreira R, Sousa C, Gonçalves RFS et al. Characterization and Genomic Analysis of a New Phage Infecting Helicobacter pylori. IJMS. 2022;23:7885. 10.3390/ijms23147885. 35887231 PMC9319048

[49] Chen S, Zhou Y, Chen Y et al. fastp: an ultra-fast all-in-one FASTQ preprocessor. Bioinformatics. 2018;34:i884–90. 10.1093/bioinformatics/bty560. 30423086 PMC6129281

[50] Chen Y, Chen Y, Shi C et al. SOAPnuke: a MapReduce acceleration-supported software for integrated quality control and preprocessing of high-throughput sequencing data. Gigascience. 2018;7:1–6. 10.1093/gigascience/gix120. PMC5788068 29220494

[51] Bankevich A, Nurk S, Antipov D et al. SPAdes: a New Genome Assembly Algorithm and Its Applications to Single-Cell Sequencing. J Comput Biol. 2012;19:455. 10.1089/cmb.2012.0021. 22506599 PMC3342519

[52] Altschul SF, Madden TL, Schäffer AA et al. Gapped BLAST and PSI-BLAST: a new generation of protein database search programs. Nucleic Acids Res. 1997;25:3389–402. 10.1093/nar/25.17.3389. 9254694 PMC146917

[53] Okonechnikov K, Conesa A, García-Alcalde F. Qualimap 2: advanced multi-sample quality control for high-throughput sequencing data. Bioinformatics. 2016;32:292–4. 10.1093/bioinformatics/btv566. 26428292 PMC4708105

[54] Miller ES, Kutter E, Mosig G et al. Bacteriophage T4 Genome. Microbiol Mol Biol Rev. 2003;67:86–156. 10.1128/MMBR.67.1.86-156.2003. 12626685 PMC150520

[55] Lanni YT . First-step-transfer deoxyribonucleic acid of bacteriophage T5. Bacteriol Rev. 1968;32:227. 10.1128/br.32.3.227-242.1968. 4879238 PMC408296

[56] Mccorquodale DJ, Shaw AR, Shaw PK et al. Pre-early polypeptides of bacteriophages T5 and BF23. J Virol. 1977;22:480. 10.1128/jvi.22.2.480-488.1977. 325230 PMC515738

[57] Roberts MD, Martin NL, Kropinski AM. The genome and proteome of coliphage T1. Virology. 2004;318:245–66. 10.1016/j.virol.2003.09.020. 14972552

[58] Garcia LR, Molineux IJ. Rate of translocation of bacteriophage T7 DNA across the membranes of Escherichia coli. J Bacteriol. 1995;177:4066–76. 10.1128/jb.177.14.4066-4076.1995. 7608081 PMC177138

[59] Bouras G, Nepal R, Houtak G et al. Pharokka: a fast scalable bacteriophage annotation tool. Bioinformatics. 2023;39:btac776. 10.1093/bioinformatics/btac776. 36453861 PMC9805569

[60] Rice P, Longden L, Bleasby A. EMBOSS: the European Molecular Biology Open Software Suite. Trends Genet. 2000;16:276–7. 10.1016/S0168-9525(00)02024-2. 10827456

[61] Garneau JR, Depardieu F, Fortier LC et al. PhageTerm: a tool for fast and accurate determination of phage termini and packaging mechanism using next-generation sequencing data. Sci Rep. 2017;7:1–10. 10.1038/s41598-017-07910-5. 28811656 PMC5557969

[62] Mcnair K, Zhou C, Dinsdale EA et al. PHANOTATE: a novel approach to gene identification in phage genomes. Bioinformatics. 2019;35:4537–42. 10.1093/bioinformatics/btz265. 31329826 PMC6853651

[63] Ecale Zhou CL, Malfatti S, Kimbrel J et al. multiPhATE: bioinformatics pipeline for functional annotation of phage isolates. Bioinformatics. 2019;35:4402–4. 10.1093/bioinformatics/btz258. 31086982 PMC6821344

[64] Chan PP, Lowe TM. tRNAscan-SE: searching for tRNA genes in genomic sequences. Methods Mol Biol. 2019;1962:1–14. 31020551 10.1007/978-1-4939-9173-0_1 PMC6768409

[65] Laslett D, Canback B. ARAGORN, a program to detect tRNA genes and tmRNA genes in nucleotide sequences. Nucleic Acids Research. 2004;32:11–6. 10.1093/nar/gkh152. 14704338 PMC373265

[66] Bouras G, Grigson SR, Mirdita M et al. Protein Structure Informed Bacteriophage Genome Annotation with Phold. Biorxiv, 10.1101/2025.08.05.668817, August 06, 2025, preprint: not peer reviewed.. PMC12774648 41495893

[67] Johnson SR, Weigele P, Fomenkov A et al. Domainator, a flexible software suite for domain-based annotation and neighborhood analysis, identifies proteins involved in antiviral systems. Nucleic Acids Res. 2025;53:gkae1175. 10.1093/nar/gkae1175. 39657740 PMC11754643

[68] Turner D, Adriaenssens EM, Tolstoy I et al. Phage annotation guide: guidelines for assembly and high-quality annotation. Phage. 2021;2:170. 10.1089/phage.2021.0013. 35083439 PMC8785237

[69] Tesson F, Huiting E, Wei L et al. Exploring the diversity of anti-defense systems across prokaryotes, phages, and mobile genetic elements. Nucleic Acids Res. 2025; 53:gkae1171. 10.1093/nar/gkae1171. PMC11724313 39657785

[70] Jia B, Raphenya AR, Alcock B et al. CARD 2017: expansion and model-centric curation of the comprehensive antibiotic resistance database. Nucleic Acids Res. 2017;45:D566–73. 10.1093/nar/gkw1004. 27789705 PMC5210516

[71] Chen L, Zheng D, Liu B et al. VFDB 2016: hierarchical and refined dataset for big data analysis-10 years on. Nucleic Acids Res. 2016;44:D694–7. 10.1093/nar/gkv1239. 26578559 PMC4702877

[72] Hockenberry AJ, Wilke CO. BACPHLIP: predicting bacteriophage lifestyle from conserved protein domains. PeerJ. 2021;9:e11396. 10.7717/peerj.11396. 33996289 PMC8106911

[73] Boeckaerts D, Stock M, De Baets B et al. Identification of phage receptor-binding protein sequences with Hidden Markov Models and an extreme gradient boosting classifier. Viruses. 2022;14:1329. 10.3390/v14061329. 35746800 PMC9230537

[74] Concha-Eloko R, Stock M, De Baets Id B et al. DepoScope: accurate phage depolymerase annotation and domain delineation using large language models. PLoS Comput Biol. 2024;20:e1011831. 10.1371/journal.pcbi.1011831. 39102416 PMC11326577

[75] Kshirsagar M, Meller A, Humphreys IR et al. Rapid and accurate prediction of protein homo-oligomer symmetry using Seq2Symm. Nat Commun. 2025;16:1–11. 10.1038/s41467-025-57148-3. 40016259 PMC11868566

[76] Kim G, Lee S, Levy Karin E et al. Easy and accurate protein structure prediction using ColabFold. Nat Protoc. 2025;20:620–42. 10.1038/s41596-024-01060-5. 39402428

[77] van Kempen M, Kim SS, Tumescheit C et al. Fast and accurate protein structure search with Foldseek. Nat Biotechnol. 2024;42:243–6. 10.1038/s41587-023-01773-0. 37156916 PMC10869269

[78] Berman HM, Westbrook J, Feng Z et al. The Protein Data Bank. Nucleic Acids Res. 2000;28:235–42. 10.1093/nar/28.1.235. 10592235 PMC102472

[79] Varadi M, Bertoni D, Magana P et al. AlphaFold Protein Structure Database in 2024: providing structure coverage for over 214 million protein sequences. Nucleic Acids Res. 2024;52:D368–75. 10.1093/nar/gkad1011. 37933859 PMC10767828

[80] Latka A, Leiman PG, Drulis-Kawa Z et al. Modelling the architecture of depolymerase-containing receptor binding proteins in Klebsiella phages. Front Microbiol. 2019;10:2649. 10.3389/fmicb.2019.02649. 31803168 PMC6872550

[81] Kelley LA, Mezulis S, Yates CM et al. The Phyre2 web portal for protein modeling, prediction and analysis. Nat Protoc. 2015;10:845–58. 10.1038/nprot.2015.053. 25950237 PMC5298202

[82] Söding J, Biegert A, Lupas AN. The HHpred interactive server for protein homology detection and structure prediction. Nucleic Acids Res. 2005;33:W244. 15980461 10.1093/nar/gki408 PMC1160169

[83] Shang J, Jiang J, Sun Y. Bacteriophage classification for assembled contigs using graph convolutional network. Bioinformatics. 2021;37:i25–i33. 10.1093/bioinformatics/btab293. 34252923 PMC8275337

[84] Bin Jang H, Bolduc B, Zablocki O et al. Taxonomic assignment of uncultivated prokaryotic virus genomes is enabled by gene-sharing networks. Nat Biotechnol. 2019;37:632–9. 10.1038/s41587-019-0100-8. 31061483

[85] Shannon P, Markiel A, Ozier O et al. Cytoscape: a software Environment for integrated models of biomolecular interaction networks. Genome Res. 2003;13:2498–504. 10.1101/gr.1239303. 14597658 PMC403769

[86] Moraru C, Varsani A, Kropinski AM. VIRIDIC—A Novel Tool to Calculate the Intergenomic Similarities of Prokaryote-Infecting Viruses. Viruses. 2020;12:1268. 10.3390/v12111268. 33172115 PMC7694805

[87] Kolde R Pheatmap: Pretty Heatmaps. 2022. https://github.com/raivokolde/pheatmap (July 2025, date last accessed).

[88] Meier-Kolthoff JP, Göker M. VICTOR: genome-based phylogeny and classification of prokaryotic viruses. Bioinformatics. 2017;33:3396–404. 10.1093/bioinformatics/btx440. 29036289 PMC5860169

[89] Letunic I, Bork P. Interactive Tree Of Life (iTOL) v5: an online tool for phylogenetic tree display and annotation. Nucleic Acids Res. 2021;49:W293–6. 10.1093/nar/gkab301. 33885785 PMC8265157

[90] Gilchrist CLM, Chooi YH. clinker & clustermap.js: automatic generation of gene cluster comparison figures. Bioinformatics. 2021;37:2473–5. 10.1093/bioinformatics/btab007. 33459763

[91] Bolger AM, Lohse M, Usadel B. Trimmomatic: a flexible trimmer for Illumina sequence data. Bioinformatics. 2014;30:2114–20. 10.1093/bioinformatics/btu170. 24695404 PMC4103590

[92] Seemann T . Prokka: rapid prokaryotic genome annotation. Bioinformatics. 2014;30:2068–9. 10.1093/bioinformatics/btu153. 24642063

[93] Stanton TD, Hetland MAK, Löhr IH et al. Fast and accurate in silico antigen typing with Kaptive 3. Microb Genom. 2025;11:1–12. 10.1099/mgen.0.001428 PMC12188004 40553506

[94] Wyres KL, Wick RR, Gorrie C et al. Identification of Klebsiella capsule synthesis loci from whole genome data. Microb Genom. 2016;2:e000102. 28348840 10.1099/mgen.0.000102 PMC5359410

[95] https://pathogen.watch/, January 2024.

[96] Hennart M, Guglielmini J, Bridel S et al. A dual barcoding approach to bacterial strain nomenclature: genomic taxonomy of Klebsiella pneumoniae strains. Mol Biol Evol. 2022;39:1–16. 10.1093/molbev/msac135. PMC9254007 35700230

[97] Lees JA, Harris SR, Tonkin-Hill G et al. Fast and flexible bacterial genomic epidemiology with PopPUNK. Genome Res. 2019;29:304–16. 10.1101/gr.241455.118. 30679308 PMC6360808

[98] Feldgarden M, Brover V, Haft DH et al. Validating the AMRFinder Tool and Resistance Gene Database by Using Antimicrobial Resistance Genotype-Phenotype Correlations in a Collection of Isolates. Antimicrob Agents Chemother. 2019;63:1–19. 10.1128/AAC.00483-19. PMC6811410 31427293

[99] Starikova EV, Tikhonova PO, Prianichnikov NA et al. Phigaro: high-throughput prophage sequence annotation. Bioinformatics. 2020;36:3882–4. 10.1093/bioinformatics/btaa250. 32311023

[100] Payne LJ, Todeschini TC, Wu Y et al. Identification and classification of antiviral defence systems in bacteria and archaea with PADLOC reveals new system types. Nucleic Acids Res. 2021;49:10868–78. 10.1093/nar/gkab883. 34606606 PMC8565338

[101] Tesson F, Hervé A, Mordret E et al. Systematic and quantitative view of the antiviral arsenal of prokaryotes. Nat Commun. 2022;13:1–10. 10.1038/s41467-022-30269-9. 35538097 PMC9090908

[102] Hadley Wickham ggplot2: elegant Graphics for Data Analysis. J R Stat Soc Ser A Stat Soc 2009;. 174:245–6.

[103] Wyres KL, Wick RR, Gorrie C et al. Identification of Klebsiella capsule synthesis loci from whole genome data. Microb Genom. 2016;2:e000102. 28348840 10.1099/mgen.0.000102 PMC5359410

[104] Feltwell T, Dorman MJ, Goulding DA et al. Separating Bacteria by Capsule Amount Using a Discontinuous Density Gradient. J Vis Exp. 2019;2019:e58679. 10.3791/58679 30663644

[105] Tisza MJ, Buck CB. A catalog of tens of thousands of viruses from human metagenomes reveals hidden associations with chronic diseases. Proc Natl Acad Sci USA. 2021;118:1–11. 10.1073/pnas.2023202118. PMC8201803 34083435

[106] Huttenhower C, Gevers D, Knight R et al. Structure, function and diversity of the healthy human microbiome. Nature. 2012;486:207–14. 22699609 10.1038/nature11234 PMC3564958

[107] Methé BA, Nelson KE, Pop M et al. A framework for human microbiome research. Nature. 2012;486:215–21. 22699610 10.1038/nature11209 PMC3377744

[108] Langmead B, Salzberg SL. Fast gapped-read alignment with Bowtie 2. Nat Methods. 2012;9:357–9. 10.1038/nmeth.1923. 22388286 PMC3322381

[109] Wood DE, Lu J, Langmead B. Improved metagenomic analysis with Kraken 2. Genome Biol. 2019;20:1–13. 10.1186/s13059-019-1891-0. 31779668 PMC6883579

[110] Li H, Handsaker B, Wysoker A et al. The Sequence Alignment/Map format and SAMtools. Bioinformatics. 2009;25:2078–9. 10.1093/bioinformatics/btp352. 19505943 PMC2723002

[111] Xu S, Chen M, Feng T et al. Use ggbreak to Effectively Utilize Plotting Space to Deal With Large Datasets and Outliers. Front Genet. 2021;12:1–7. 10.3389/fgene.2021.774846. PMC8593043 34795698

[112] Bikel S, López-Leal G, Cornejo-Granados F et al. Gut dsDNA virome shows diversity and richness alterations associated with childhood obesity and metabolic syndrome. iScience. 2021;24:1–23. 10.1016/j.isci.2021.102900. PMC8361208 34409269

[113] Liang G, Zhao C, Zhang H et al. The stepwise assembly of the neonatal virome is modulated by breastfeeding. Nature. 2020;581:470–4. 10.1038/s41586-020-2192-1. 32461640 PMC7263352

[114] Shkoporov AN, Clooney AG, Sutton TDS et al. The human gut virome is highly diverse, stable, and individual specific. Cell Host & Microbe. 2019;26:527–41. 10.1016/j.chom.2019.09.009. 31600503

[115] Yachida S, Mizutani S, Shiroma H et al. Metagenomic and metabolomic analyses reveal distinct stage-specific phenotypes of the gut microbiota in colorectal cancer. Nat Med. 2019;25:968–76. 10.1038/s41591-019-0458-7. 31171880

[116] Nayfach S, Camargo AP, Schulz F et al. CheckV assesses the quality and completeness of metagenome-assembled viral genomes. Nat Biotechnol. 2021;39:578–85. 10.1038/s41587-020-00774-7. 33349699 PMC8116208

[117] Olm MR, Brown CT, Brooks B et al. DRep: a tool for fast and accurate genomic comparisons that enables improved genome recovery from metagenomes through de-replication. ISME Journal. 2017;11:2864–8. 10.1038/ismej.2017.126. 28742071 PMC5702732

[118] Bushnell B . BBMap. 2015.

[119] Li H . Minimap2: pairwise alignment for nucleotide sequences. Bioinformatics. 2018;34:3094–100. 10.1093/bioinformatics/bty191. 29750242 PMC6137996

[120] Jain C, Rodriguez-R LM, Phillippy AM et al. High throughput ANI analysis of 90K prokaryotic genomes reveals clear species boundaries. Nat Commun. 2018;9:1–8. 10.1038/s41467-018-07641-9. 30504855 PMC6269478

[121] Olm MR, Crits-Christoph A, Bouma-Gregson K et al. InStrain enables population genomic analysis from metagenomic data and sensitive detection of shared microbial strains. Nat Biotechnol. 2021;39:727–36. 10.1038/s41587-020-00797-0. 33462508 PMC9223867

[122] Arndt D, Grant JR, Marcu A et al. PHASTER: a better, faster version of the PHAST phage search tool. Nucleic Acids Res. 2016;44:W16–21. 10.1093/nar/gkw387. 27141966 PMC4987931

[123] Quinlan AR, Hall IM. BEDTools: a flexible suite of utilities for comparing genomic features. Bioinformatics. 2010;26:841–2. 10.1093/bioinformatics/btq033. 20110278 PMC2832824

[124] Vasimuddin MD, Misra S, Li H et al. 2019; Efficient architecture-aware acceleration of BWA-MEM for multicore systems. IEEE International Parallel and Distributed Processing Symposium, 10.1109/IPDPS.2019.00041.

[125] Hahne F, Ivanek R. Visualizing genomic data using Gviz and bioconductor. Methods Mol Biol. 2016;1418:335–51. 27008022 10.1007/978-1-4939-3578-9_16

[126] Turner D, Adriaenssens EM, Amann RI et al. Summary of taxonomy changes ratified by the International Committee on Taxonomy of Viruses (ICTV) from the Bacterial Viruses Subcommittee, 2025. J Gen Virol. 2025;106:1–44. 10.1099/jgv.0.002111. PMC12451628 40711892

[127] Zhou Y, Wu C, Wang B et al. Characterization difference of typical KL1, KL2 and ST11-KL64 hypervirulent and carbapenem-resistant Klebsiella pneumoniae. Drug Resist Updat. 2023;67:100918. 10.1016/j.drup.2023.100918. 36610180

[128] Yeh KM, Kurup A, Siu LK et al. Capsular serotype K1 or K2, rather than magA and rmpA, is a major virulence determinant for Klebsiella pneumoniae liver abscess in Singapore and Taiwan. J Clin Microbiol. 2007;45:466–71. 10.1128/JCM.01150-06. 17151209 PMC1829066

[129] Remya P, Shanthi M, Sekar U. Occurrence and characterization of hyperviscous K1 and K2 serotype in Klebsiella pneumoniae. J Lab Phys. 2018;10:283–8. 10.4103/JLP.JLP_48_18 PMC6052812 30078963

[130] Tan YH, Chen Y, Chu WHW et al. Cell envelope defects of different capsule-null mutants in K1 hypervirulent Klebsiella pneumoniae can affect bacterial pathogenesis. Molecular Microbiology. 2020;113:889–905. 10.1111/mmi.14447. 31912541 PMC7317392

[131] Artyszuk D, Jachymek W, Izdebski R et al. The OL101 O antigen locus specifies a novel Klebsiella pneumoniae serotype O13 structure. Carbohydrate Polymers. 2024;326:1–9. 10.1016/j.carbpol.2023.121581. 38142087

[132] Crosa JH, Walsh CT. Genetics and Assembly Line Enzymology of Siderophore Biosynthesis in Bacteria. Microbiol Mol Biol Rev. 2002;66:223–49. 10.1128/MMBR.66.2.223-249.2002. 12040125 PMC120789

[133] March C, Moranta D, Regueiro V et al. Klebsiella pneumoniae Outer Membrane Protein A Is Required to Prevent the Activation of Airway Epithelial Cells. J Biol Chem. 2011;286:9956–67. 10.1074/jbc.M110.181008. 21278256 PMC3060550

[134] Shen L, Zhang J, Xue J et al. Regulation of ECP fimbriae-related genes by the transcriptional regulator RcsAB in Klebsiella pneumoniae NTUH-K2044. J Basic Microbiol. 2022;62:593–603. 10.1002/jobm.202100595. 35132658

[135] Grass G, Otto M, Fricke B et al. FieF (YiiP) from Escherichia coli mediates decreased cellular accumulation of iron and relieves iron stress. Arch Microbiol. 2005;183:9–18. 10.1007/s00203-004-0739-4. 15549269

[136] Yang J, Long H, Hu Y et al. Klebsiella oxytoca Complex: update on Taxonomy, Antimicrobial Resistance, and Virulence. Clin Microbiol Rev. 2022;35:1–39. 10.1128/CMR.00006-21. PMC8635272 34851134

[137] Rodríguez-Medina N, Barrios-Camacho H, Duran-Bedolla J et al. Klebsiella variicola: an emerging pathogen in humans. Emerging Microbes & Infections. 2019;8:973–88. 10.1080/22221751.2019.1634981. 31259664 PMC6609320

[138] Majkowska-Skrobek G, Markwitz P, Sosnowska E et al. The evolutionary trade-offs in phage-resistant Klebsiella pneumoniae entail cross-phage sensitization and loss of multidrug resistance. Environmental Microbiology. 2021;23:7723–40. 10.1111/1462-2920.15476. 33754440

[139] Jumper J, Evans R, Pritzel A et al. Highly accurate protein structure prediction with AlphaFold. Nature. 2021;596:583–9. 10.1038/s41586-021-03819-2. 34265844 PMC8371605

[140] García-Doval C, Castón JR, Luque D et al. Structure of the Receptor-Binding Carboxy-Terminal Domain of the Bacteriophage T5 L-Shaped Tail Fibre with and without Its Intra-Molecular Chaperone. Viruses. 2015;7:6424–40. 10.3390/v7122946. 26670244 PMC4690869

[141] Dunne M, Denyes JM, Arndt H et al. Salmonella Phage S16 Tail Fiber Adhesin Features a Rare Polyglycine Rich Domain for Host Recognition. Structure. 2018;26:1573–82. 10.1016/j.str.2018.07.017. 30244968

[142] van den Berg B, Silale A, Baslé A et al. Structural basis for host recognition and superinfection exclusion by bacteriophage T5. Proc Natl Acad Sci USA. 2022;119:1–9. 10.1073/pnas.2211672119. PMC9586334 36215462

[143] Buffet A, Rocha EPC, Rendueles O. Nutrient conditions are primary drivers of bacterial capsule maintenance in Klebsiella. Proceedings of the Royal Society B. 2021;288:1–10. 10.1098/rspb.2020.2876 PMC7935059 33653142

[144] Blundell-Hunter G, Enright MC, Negus D et al. Characterisation of Bacteriophage-Encoded Depolymerases Selective for Key Klebsiella pneumoniae Capsular Exopolysaccharides. Front Cell Infect Microbiol. 2021;11:1–16. 10.3389/fcimb.2021.686090. PMC8253255 34222050

[145] Samson JE, Magadán AH, Sabri M et al. Revenge of the phages: defeating bacterial defences. Nat Rev Micro. 2013;11:675–87. 10.1038/nrmicro3096. 23979432

[146] Bikard D, Marraffini LA. Innate and adaptive immunity in bacteria: mechanisms of programmed genetic variation to fight bacteriophages. Current Opinion in Immunology. 2012;24:15–20. 10.1016/j.coi.2011.10.005. 22079134

[147] Hasan M, Ahn J. Evolutionary Dynamics between Phages and Bacteria as a Possible Approach for Designing Effective Phage Therapies against Antibiotic-Resistant Bacteria. Antibiotics. 2022;11:915. 10.3390/antibiotics11070915. 35884169 PMC9311878

[148] Boeckaerts D, Stock M, Ferriol-González C et al. Prediction of Klebsiella phage-host specificity at the strain level. Nat Commun. 2024;15:1–10. 10.1038/s41467-024-48675-6. 38778023 PMC11111740

[149] Gaborieau B, Vaysset H, Tesson F et al. Prediction of strain level phage–host interactions across the Escherichia genus using only genomic information. Nat Microbiol. 2024;9:2847–61. 10.1038/s41564-024-01832-5. 39482383

[150] Yang F, Jiang YL, Zhang JT et al. Fine structure and assembly pattern of a minimal myophage Pam3. Proc Natl Acad Sci USA. 2023;120:e2213727120. 10.1073/pnas.2213727120. 36656854 PMC9942802

[151] Guerin E, Shkoporov A, Stockdale SR et al. Biology and taxonomy of crAss-like bacteriophages, the most abundant virus in the human gut. Cell Host & Microbe. 2018;24:653–64. 10.1016/j.chom.2018.10.002. 30449316

[152] Benler S, Yutin N, Antipov D et al. Thousands of previously unknown phages discovered in whole-community human gut metagenomes. Microbiome. 2021;9:1–17. 10.1186/s40168-021-01017-w. 33781338 PMC8008677

[153] Camarillo-Guerrero LF, Almeida A, Rangel-Pineros G et al. Massive expansion of human gut bacteriophage diversity. Cell. 2021;184:1098–1109. 10.1016/j.cell.2021.01.029. 33606979 PMC7895897

[154] Edwards RA, Vega AA, Norman HM et al. Global phylogeography and ancient evolution of the widespread human gut virus crAssphage. Nat Microbiol. 2019;4:1727–36. 10.1038/s41564-019-0494-6. 31285584 PMC7440971

[155] Shkoporov AN, Khokhlova EV, Stephens N et al. Long-term persistence of crAss-like phage crAss001 is associated with phase variation in Bacteroides intestinalis. BMC Biol. 2021;19:1–16. 10.1186/s12915-021-01084-3. 34407825 PMC8375218

[156] Schmidtke DT, Hickey AS, Wirbel J et al. The prototypic crAssphage is a linear phage-plasmid. Cell Host & Microbe. 2025;33:1347–62. 10.1016/j.chom.2025.07.004. 40730160 PMC12358190

[157] Turnbaugh PJ, Ley RE, Hamady M et al. The Human Microbiome Project. Nature. 2007;449:804–10. 10.1038/nature06244. 17943116 PMC3709439

[158] Dong N, Yang X, Chan EWC et al. Klebsiella species: taxonomy, hypervirulence and multidrug resistance. EBioMedicine. 2022;79:103998. 10.1016/j.ebiom.2022.103998. 35405387 PMC9010751

[159] Clegg S, Murphy CN. Epidemiology and Virulence of Klebsiella pneumonia. Microbiol Spectr. 2016;4:1–17. 10.1128/microbiolspec.UTI-0005-2012. 26999397

[160] Vornhagen J, Rao K, Bachman MA. Gut community structure as a risk factor for infection in Klebsiella pneumoniae-colonized patients. Msystems. 2024;9:1–18. 10.1128/msystems.00786-24. PMC11334466 38975759

[161] Le Guern R, Grandjean T, Stabler S et al. Gut colonisation with multidrug-resistant Klebsiella pneumoniae worsens Pseudomonas aeruginosa lung infection. Nat Commun. 2023;14:1–12. 10.1038/s41467-022-35767-4. 36604442 PMC9816093

[162] Jiménez-Rojas V, Villanueva-García D, Miranda-Vega AL et al. Gut colonization and subsequent infection of neonates caused by extended-spectrum beta-lactamase-producing Escherichia coli and Klebsiella pneumoniae. Front Cell Infect Microbiol. 2023;13:1322874. 10.3389/fcimb.2023.1322874. 38314094 PMC10834783

[163] De Maio F, Bianco DM, Santarelli G et al. Profiling the gut microbiota to assess infection risk in Klebsiella pneumoniae-colonized patients. Gut Microbes. 2025;17:2468358. 10.1080/19490976.2025.2468358. 39964311 PMC11845061

[164] Unverdorben LV, Pirani A, Gontjes K et al. Klebsiella pneumoniae evolution in the gut leads to spontaneous capsule loss and decreased virulence potential. mBio. 2025;16:e02362–24. 10.1128/mbio.02362-24. 40162782 PMC12077207

[165] Elek CKA, Brown TL, Viet T et al. A hybrid and poly-polish workflow for the complete and accurate assembly of phage genomes: a case study of ten przondoviruses. Microb Genom. 2023;9:1–14. 10.1099/mgen.0.001065 PMC10438801 37463032

[166] Ferriol-González C, Concha-Eloko R, Bernabéu-Gimeno M et al. Targeted phage hunting to specific Klebsiella pneumoniae clinical isolates is an efficient antibiotic resistance and infection control strategy. Microbiol Spectr. 2024;12:e00254–24. 39194291 10.1128/spectrum.00254-24 PMC11448410

[167] Thomas JA, Orwenyo J, Wang LX et al. The Odd “RB” Phage—Identification of Arabinosylation as a New Epigenetic Modification of DNA in T4-Like Phage RB69. Viruses. 2018;10:313. 10.3390/v10060313. 29890699 PMC6024577

[168] Kortright KE, Chan BK, Turner PE. High-throughput discovery of phage receptors using transposon insertion sequencing of bacteria. Proc Natl Acad Sci USA. 2020;117:18670–9. 10.1073/pnas.2001888117. 32675236 PMC7414163

[169] Pacios O, Fernández-García L, Bleriot I et al. Phenotypic and Genomic Comparison of Klebsiella pneumoniae Lytic Phages: vB_KpnM-VAC66 and vB_KpnM-VAC13. Viruses. 2021;14:6. 10.3390/v14010006. 35062209 PMC8778798

[170] Ellis EL, Delbrück M. The growth of bacteriophage. J Gen Physiol. 1939;22:365–84. 10.1085/jgp.22.3.365. 19873108 PMC2141994

[171] Luria SE, Delbrück M. Mutations of bacteria from virus sensitivity to virus resistance. Genetics. 1943;28:491–511. 10.1093/genetics/28.6.491. 17247100 PMC1209226

[172] Hershey AD, Chase M. Independent functions of viral protein and nucleic acid in growth of bacteriophage. J Gen Physiol. 1952;36:39–56. 10.1085/jgp.36.1.39. 12981234 PMC2147348

[173] Rohwer F, Segall AM. In Retrospect: A Century of Phage Lessons. Nature. 2015;528:46–8. 26632584 10.1038/528046a

[174] Gan L, Feng Y, Du B et al. Bacteriophage targeting microbiota alleviates non-alcoholic fatty liver disease induced by high alcohol-producing Klebsiella pneumoniae. Nat Commun. 2023;14:1–15. 10.1038/s41467-023-39028-w. 37270557 PMC10239455

[175] Tan YH, Chen Y, Chu WHW et al. Cell envelope defects of different capsule-null mutants in K1 hypervirulent Klebsiella pneumoniae can affect bacterial pathogenesis. Molecular Microbiology. 2020;113:889–905. 10.1111/mmi.14447. 31912541 PMC7317392

[176] Haudiquet M, Buffet A, Rendueles O et al. Interplay between the cell envelope and mobile genetic elements shapes gene flow in populations of the nosocomial pathogen Klebsiella pneumoniae. PLoS Biol. 2021;19:e3001276. 10.1371/journal.pbio.3001276. 34228700 PMC8259999

[177] Short FL, Di Sario G, Reichmann NT et al. Genomic profiling reveals distinct routes to complement resistance in klebsiella pneumoniae. Infect Immun. 2020;88:e00043–20. 10.1128/IAI.00043-20. 32513855 PMC7375759

[178] Huang TW, Lam I, Chang HY et al. Capsule deletion via a λ-Red knockout system perturbs biofilm formation and fimbriae expression in Klebsiella pneumoniae MGH 78578. BMC Res Notes. 2014;7:13. 10.1186/1756-0500-7-13. 24398052 PMC3892127

[179] Pal S, Verma J, Mallick S et al. Absence of the glycosyltransferase wcaj in klebsiella pneumoniae atcc13883 affects biofilm formation, increases polymyxin resistance and reduces murine macrophage activation. Microbiology. 2019;165:891–904. 10.1099/mic.0.000827. 31246167

[180] Sahly H, Podschun R, Oelschlaeger TA et al. Capsule impedes adhesion to and invasion of epithelial cells by Klebsiella pneumoniae. Infect Immun. 2000;68:6744–9. 10.1128/IAI.68.12.6744-6749.2000. 11083790 PMC97775

[181] Lin TH, Wu CC, Kuo JT et al. Fnr-dependent rmpa and rmpa2 regulation of capsule polysaccharide biosynthesis in Klebsiella pneumoniae. Front Microbiol. 2019;10:1–12. 10.3389/fmicb.2019.02436. 31736888 PMC6828653

[182] Ernst CM, Braxton JR, Rodriguez-Osorio CA et al. Adaptive evolution of virulence and persistence in carbapenem-resistant Klebsiella pneumoniae. Nat Med. 2020;26:705–11. 10.1038/s41591-020-0825-4. 32284589 PMC9194776

[183] Dawson SJT, Shibu P, Garnett S et al. Weberviruses are gut-associated phages that infect Klebsiella spp. FEMS Microbiol Ecol. 2025;101:43. 10.1093/femsec/fiaf043. PMC12023860 40251011

[184] Lee SY, Yang J, Lee JH. Improvement of crAssphage detection/quantification method and its extensive application for food safety. Front Microbiol. 2023;14:1185788. 10.3389/fmicb.2023.1185788. 37256047 PMC10225732

[185] Stachler E, Kelty C, Sivaganesan M et al. Quantitative CrAssphage PCR Assays for Human Fecal Pollution Measurement. Environ Sci Technol. 2017;51:9146. 10.1021/acs.est.7b02703. 28700235 PMC7350147

[186] Li E, Saleem F, Edge TA et al. Assessment of crAssphage as a human fecal source tracking marker in the lower Great Lakes. Sci Total Environ. 2024;912:168840. 10.1016/j.scitotenv.2023.168840. 38036144

[187] Zolnier C, Molloy J 2024; Open Biosharing Workshops - Report.

