# Supplementary material for: KlebPhaCol: a community-driven resource for Klebsiella research identified a novel phage family
Source: Nucleic Acids Res. 2025 Nov 20;53(21):gkaf1122. doi: 10.1093/nar/gkaf1122 (PMC12629751; doi:10.1093/nar/gkaf1122)
Supplement: gkaf1122_Supplemental_Files [file gkaf1122_supplemental_files.zip › Supplementary Data-revised.docx]

**Supplementary data**

**KlebPhaCol: A community-driven resource for *Klebsiella* research identified a novel phage family**

Daniela Rothschild-Rodriguez^1^, Kai S. Lambon^1^, Simran Krishnakant Kushwaha^1^, Sofya K. Garushyants^2^, Moritz Ertelt^3,4^, Agnieszka Latka^5,6^, Ana Rita Costa^7^, Anna Mantzouratou^8^, Claire King^1^, Dimitri Boeckaerts^5,9^, Elizabeth Sheridan^8,10,^, Eugene V. Koonin^2^, Francesca Merrick^1^, Francis Drobniewski^8,10^, Ilaria De Angelis^5^, Kordo Saeed^11^, Macy Martin^1^, J. Mark Sutton^12,13^, Matthew E. Wand^12^, Michael Andrew^1^, Morgen Hedges^1^, Stan J. J. Brouns^7^, Pieter-Jan Haas^14^, Sophie T. Lawson^12,13^, Stephen M.E. Fordham^8^, Yan-Jiun Lee^15^, Yi Wu^1^, Yves Briers^5^, Peter Braun^3,4^, Peter R. Weigele^15^, and Franklin L. Nobrega^1^

**Table of Contents**

**Supplementary Figures**

**Supplementary Figure S1.** Bacteria-encoded defence systems and correlation analyses with infectivity patterns.

**Supplementary Figure S2.** Roth phage host range stratified by sequence (ST), capsule locus (KL), and O-antigen types.

**Supplementary Figure S3.** Capsule loci assemblies of the 17 isolation hosts of the KlebPhaCol phages.

**Supplementary Figure S4.** Klebsiella Phage Collection host range of 52 phages vs 74 strains in TSB broth.

**Supplementary Figure** **S5.** Growth curves of ST323-targeting phages in susceptible strains

**Supplementary Figure S6.** Expanded core genome annotations of RothD.

**Supplementary Figure S7.** Full read coverage of RothD lysogens.

**Supplementary Text and figures**

**Supplementary Text Figure 1.** Transmission electron microscope images of all KlebPhaCol phages.

**Supplementary Text Figure 2.** Genome synteny plots of representative Roth phages with relatives.

**Supplementary Text Figure 3.** One-step growth curves and burst size of all representative phages.

**Supplementary Text Figure 4.** Predicted protein structures of computationally curated Receptor Binding Proteins (RBPs) of KlebPhaCol phages.

**Supplementary Text Figure 5.** Plaque morphologies of all KlebPhaCol phages.

**Supplementary Text Figure 6.** DNA-modifying enzymes of Roth phages.

**Supplementary Text Figure 7.** Taxonomic classification for RothD – *Felixviridae* family.

**Supplementary Text Figure 8.** *Felixviridae* and related phages undergo frequent recombination.

**Supplementary Figures**


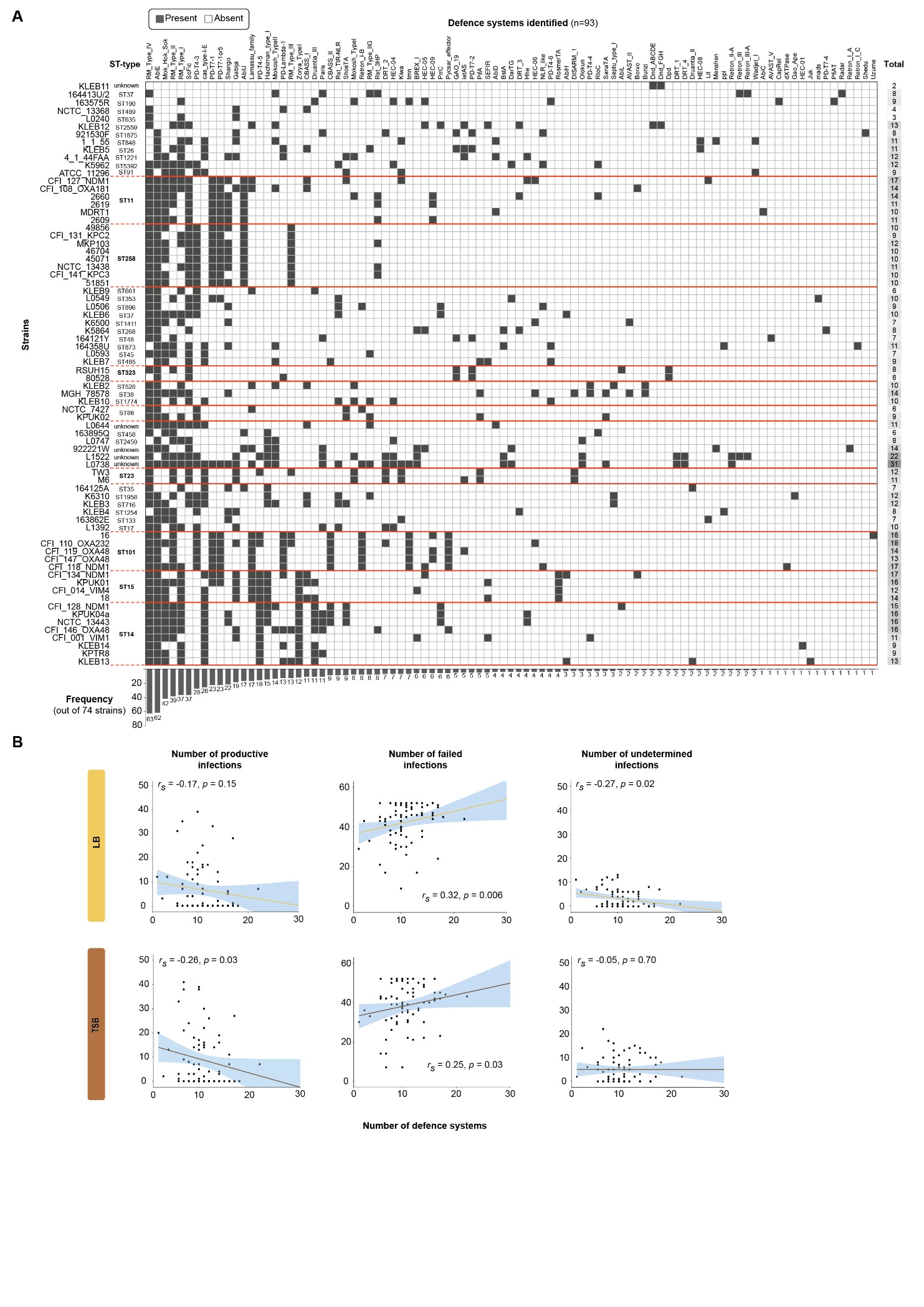


**Supplementary Figure S1.** Bacteria-encoded defence systems and correlation analyses with infectivity patterns. **(A)** Defence systems predicted by PADLOC and DefenseFinder, showing only those experimentally verified as defence systems (heatmap is not showing PDC and VSPR systems, full table can be found in Supplementary Table S1). Total count of defence systems encoded by each strain is shown on the far right. Frequency of the defence system in all 74 strains is indicated in the bottom of the heatmap. Strains are phylogenetically ordered, ST-type is indicated and grouped by the red lines. Defence systems are ordered by decreasing frequency. **(B)** Spearman rank correlation analysis was conducted and plotted using Rstudio ggplot2 to assess the relationship between the infectivity patterns of each strain and the number of encoded defence systems, considering only those systems that have been experimentally verified as showed in (A).


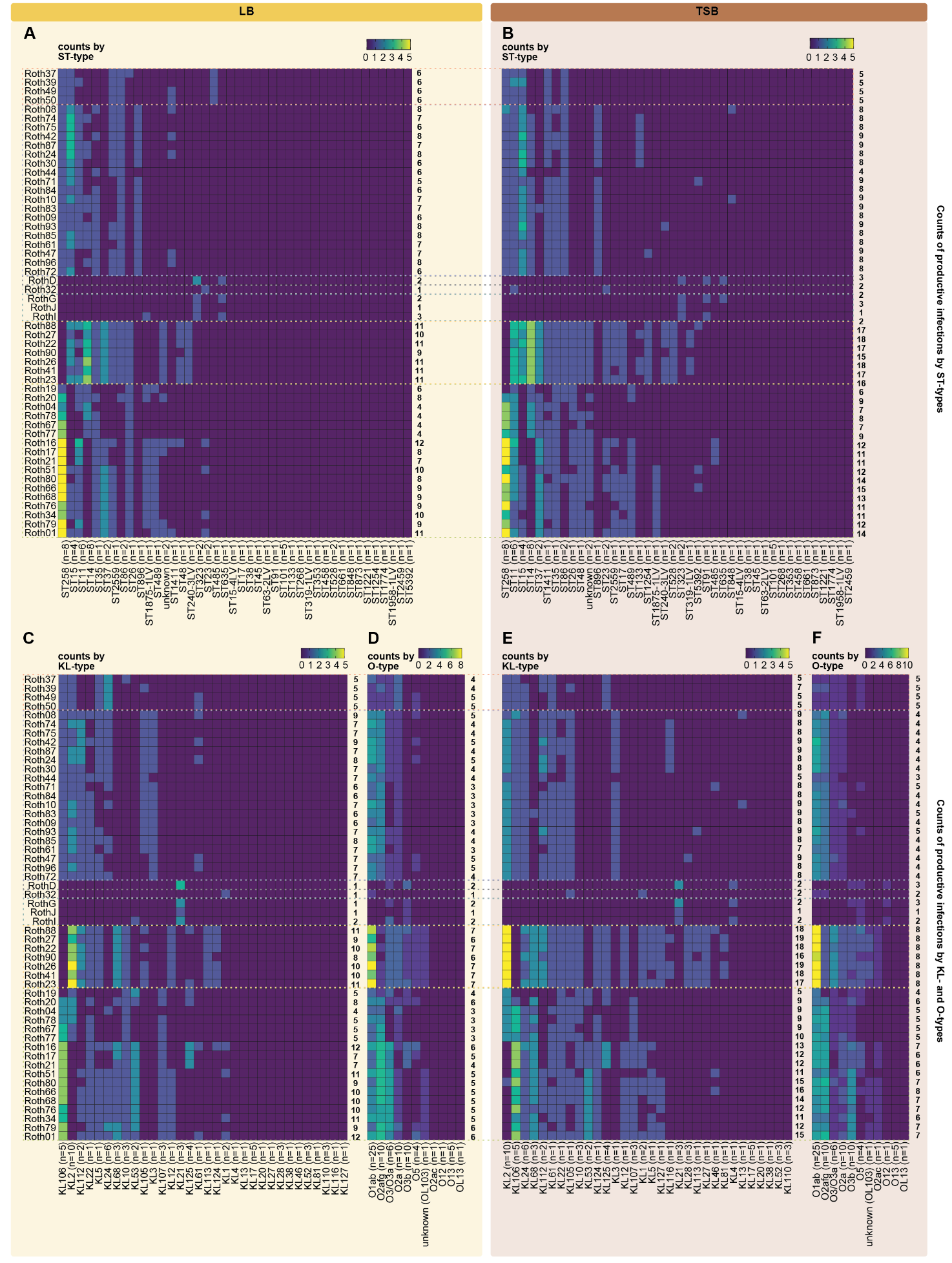
**Supplementary Figure S2.** Roth phage host range stratified by sequence (ST), capsule locus (KL), and O-antigen types. **(A)** Counts of ST-types infected per phage in LB medium. Total counts per phage are given at the right of the heatmap. Phages are ordered phylogenetically while ST-types are ordered in decreasing order of susceptibility. ST-types are followed by the total number of strains in the collection with that ST-type. **(B)** Same as (A) but for TSB medium. **(C)** Counts of KL-types infected per phage, and **(D)** O-antigen types infected per phage in LB. Phage and KL-type distribution follow the same logic as in (A). **(E) (F)** Same as (C) and (D) but in TSB medium.


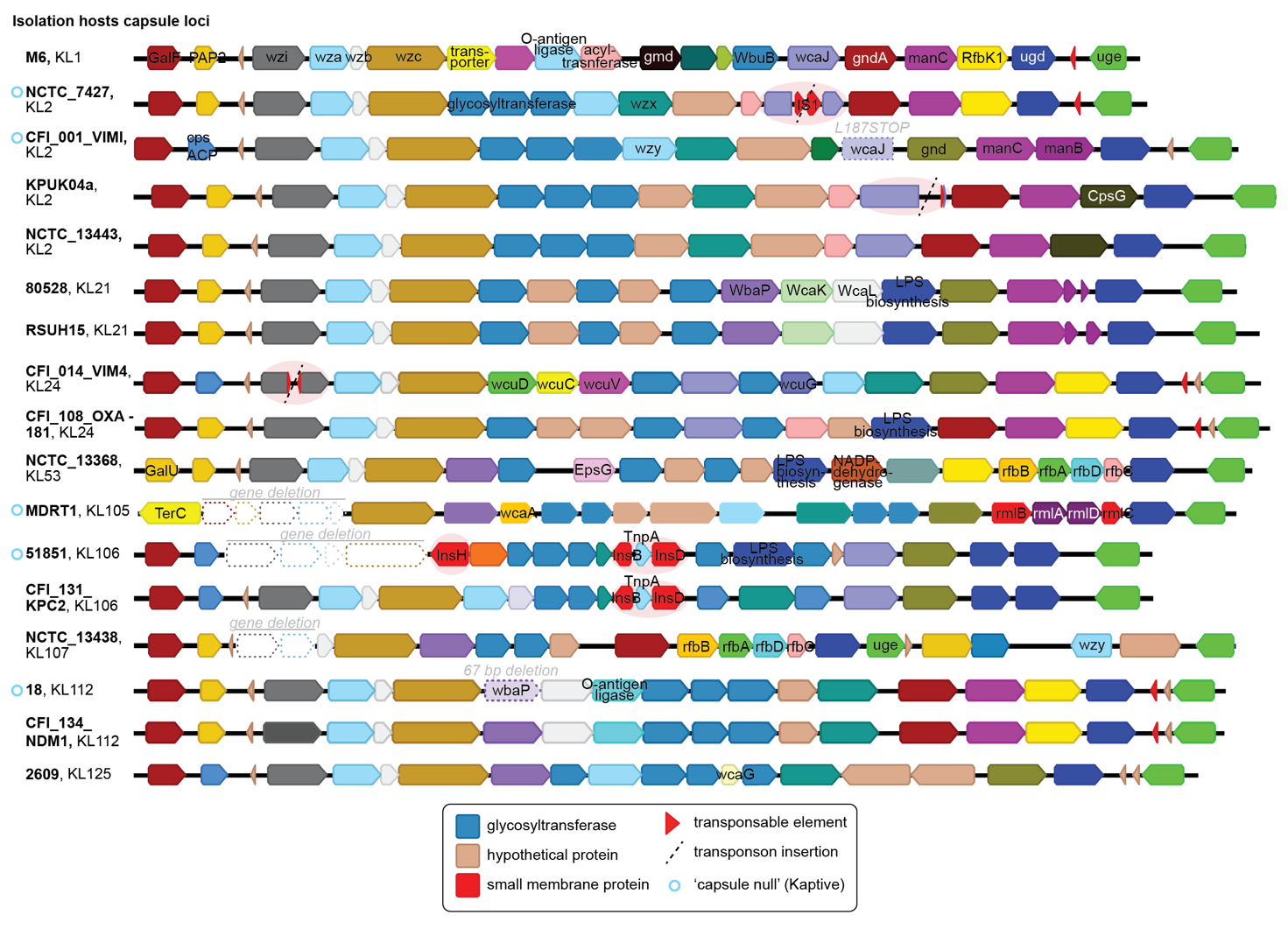


**Supplementary Figure S3.** Capsule loci assemblies of the 17 isolation hosts of the KlebPhaCol phages. Capsule disruptions are highlighted.

**
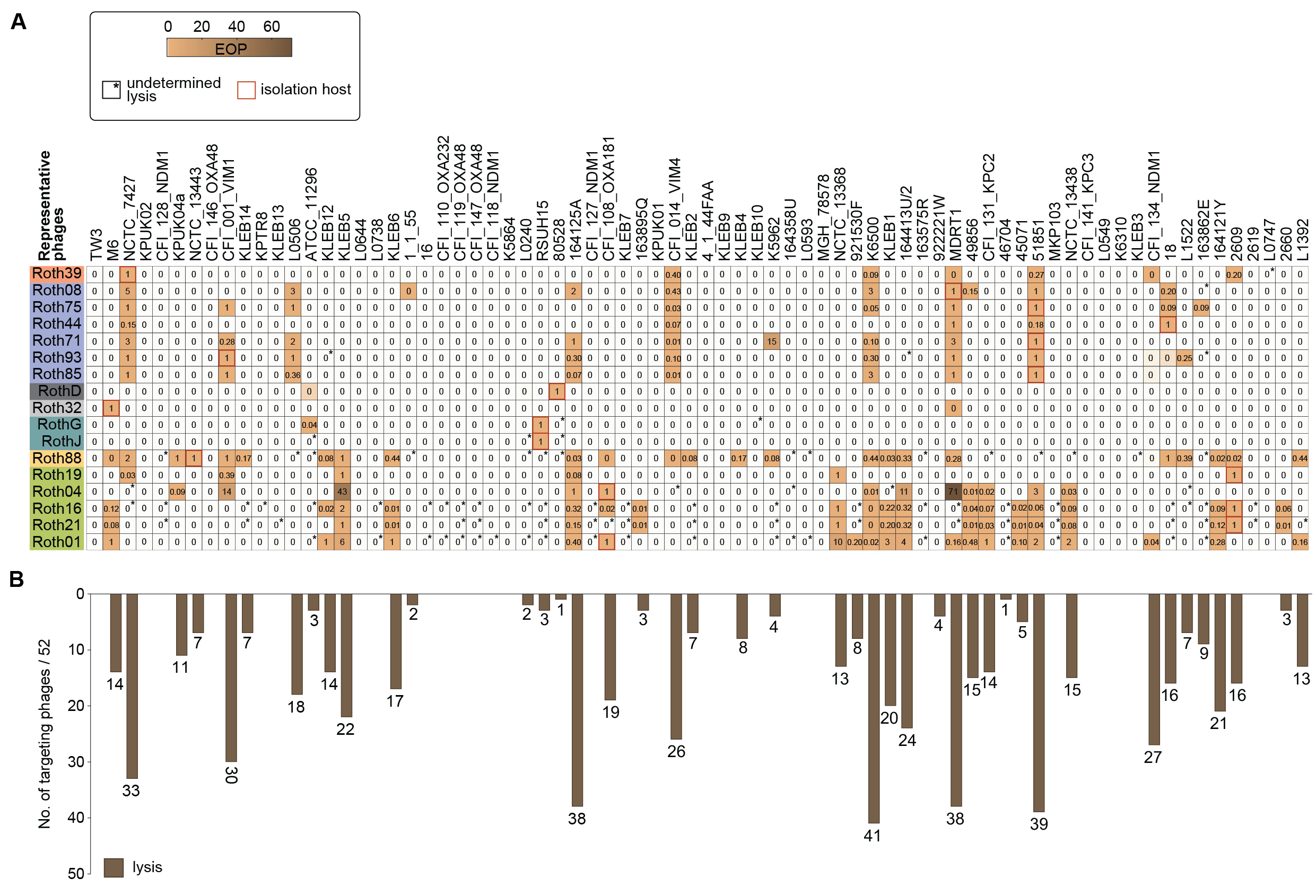
**

**Supplementary Figure S4.** Klebsiella Phage Collection host range of 52 phages vs 74 strains in TSB broth. **(A)** Host-range of representative phages (17/52 KlebPhaCol phages) in TSB broth shown as efficiency of plating (EOP) where the phage titre (PFU/mL) of the tested strain was divided by the titre on its respective isolation strain (red boxes). An asterisk indicates spots with undetermined lysis behaviour against the corresponding strain. **(B)** Quantification of each strain susceptibility (i.e. lysis) to all 52 KlebPhaCol phages.


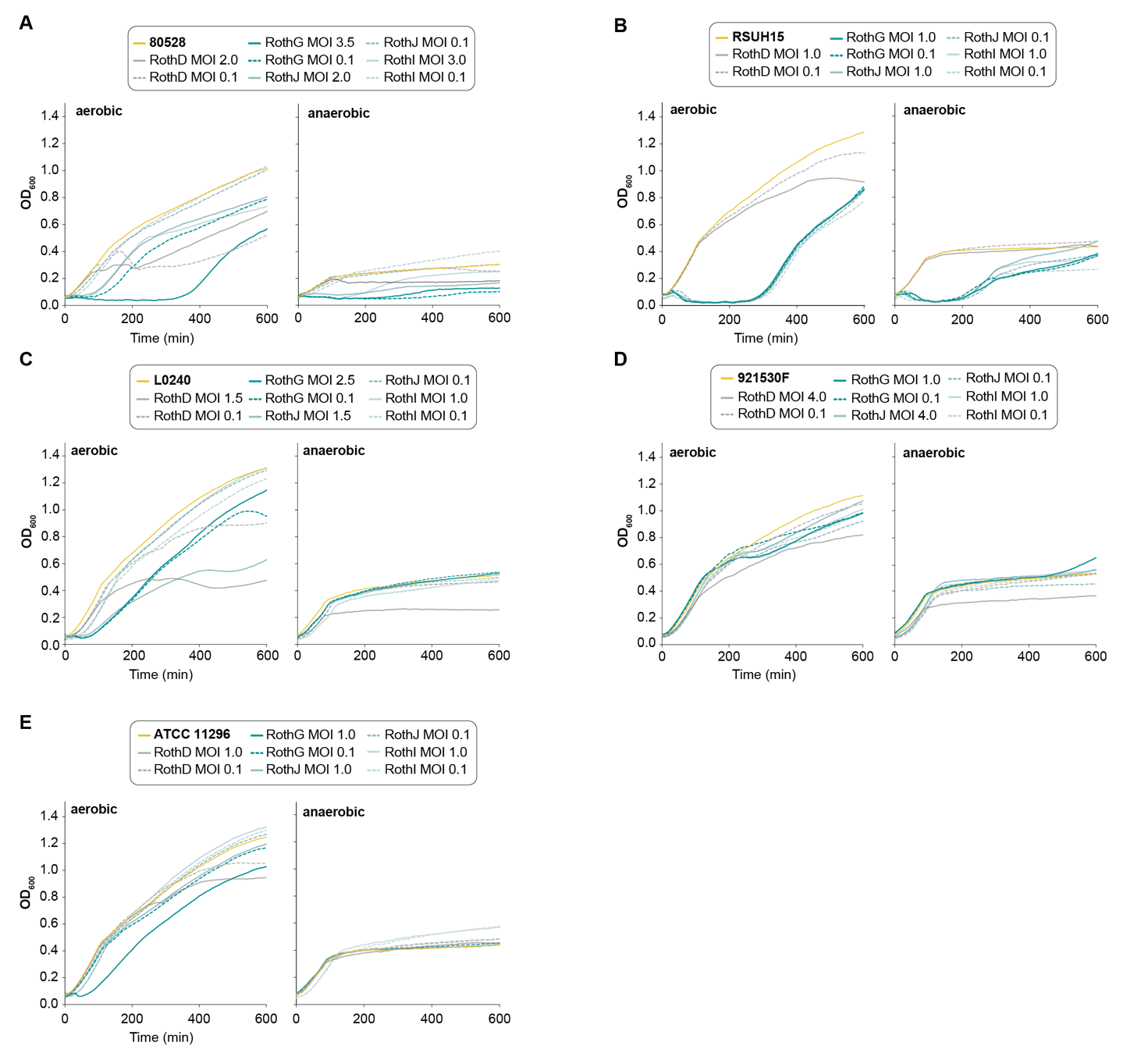
**Supplementary Figure S5.** Growth curves of ST323-targeting phages in susceptible strains. **(A-E)** Curves are the mean of three biological repeats. Bacterial control is shown in yellow, lower multiplicity of infection (MOI) curves are indicated by dashed lines.


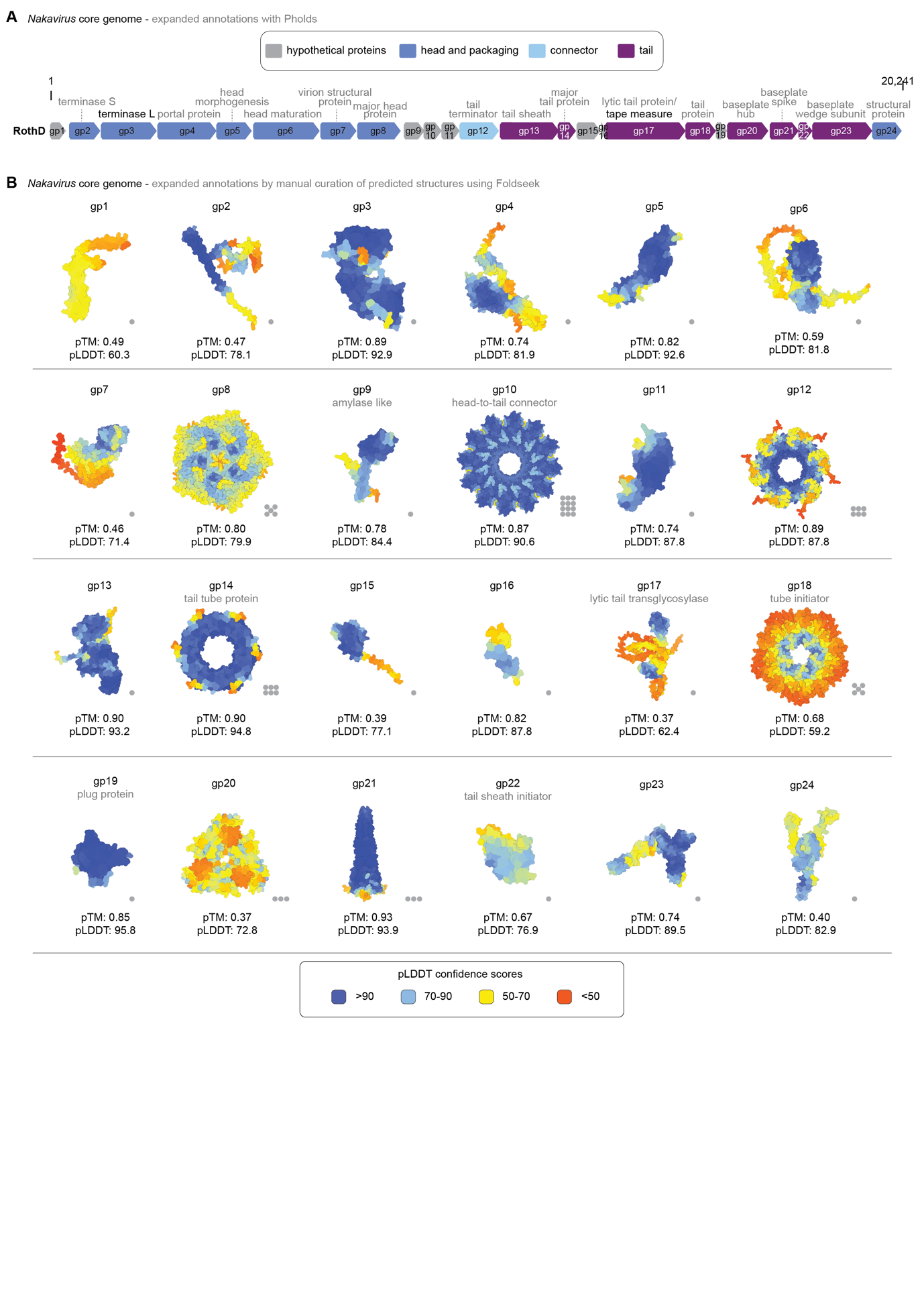
**Supplementary Figure S6.** Expanded core genome annotations of RothD. **(A)** Core genome of RothD gp1 – gp24 (1-20,241 bp) annotated using sequence-based homology via MultiPhate annotation tool (black annotations, ‘Terminase L’ and ‘Tape measure’), and structure-based homology via Phold (grey annotations). **(B)** Structural core protein predictions with AlphaFold2. The number of repeating units shown is represented by grey circles at the bottom right of each structure. Structures are coloured based on pLDDT confidence scores. Comparison of structures to protein structure databases revealed additional putative functions for gp9, gp10, gp14, gp17, gp18, gp19, and gp22 (light grey annotation).

**
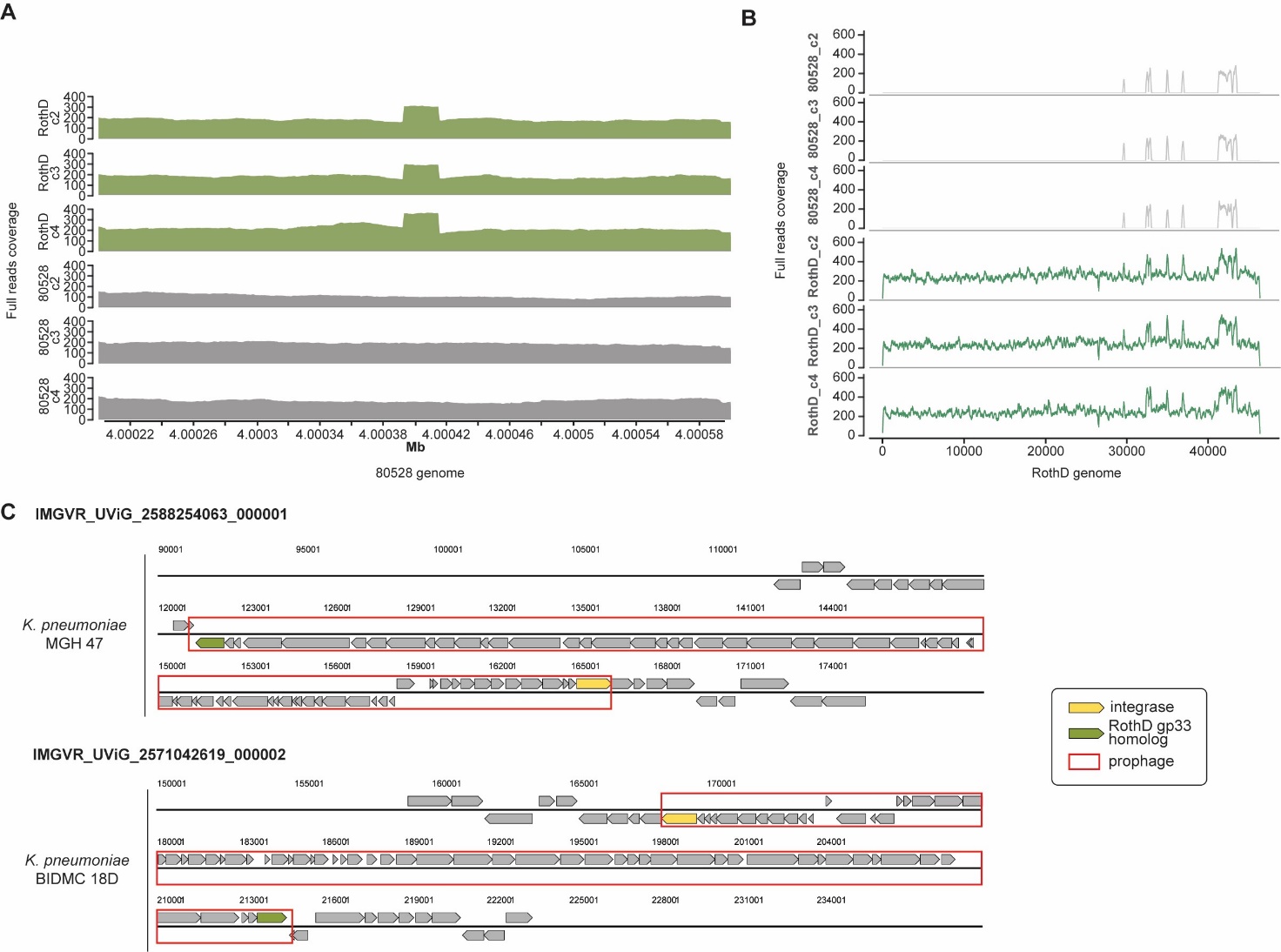
**

**Supplementary Figure S7.** Full read coverage of RothD lysogens. **(A)** All reads mapped to host strain 80528. Showing only the lysogen insertion region for all six colonies. **(B)** All reads mapped to RothD genome for all six colonies. **(C)** The two closest IMG/VR prophage relatives to RothD and their insertion site in the respective *Klebsiella* host strains, illustrating the same insertion genome architecture as integrated RothD with the integrase and a RothD gp33 homolog at both ends of the prophage region.

**Supplementary Methods**

**Transmission electron microscopy**

One mL of each phage lysate was sedimented at 21,000 × *g* for 1 h, the top 900 µL were discarded and the remaining volume was resuspended in 900 µL of sterile diH_2_O water. The centrifugation and washing steps were repeated once more. Concentrated phages (5 µL) were deposited and incubated for 30 sec on glow-discharged carbon-coated type-B 400 mesh grids (Ted Pella, USA). Excess phage was dried with filter paper before staining with 5% Ammonium Molybdate (w/v) and 0.1-1% Trehalose for 5-10 sec. Samples were examined in a transmission electron microscope FEI Tecnai T12 (FEI, USA) at an acceleration voltage of 80 or 120 kV, and phage particles were examined at 16,500–105,000 x magnification. Fiji software v2.9.0/2.14.0 (1) was used to measure the phage lengths and to crop the images to scale.

**One-step growth curves**

Overnight cultures of the isolation strains were diluted 1:100 in LB and incubated at 37 °C at 180 rpm up to an optical density (OD_600_) of 0.4-0.5. Ten mL of culture were then centrifuged at 4-7,000 x g for 5 min and the cell pellet resuspended in 5 mL of LB. Five mL of phage [10^5^ PFU/mL] were then added and let adsorb for 5 min at 37 °C at 180 rpm. The centrifugation step was repeated, and the cell pellet resuspended in 10 mL of LB, transferred into a conical flask, and incubated at 37 °C at 180 rpm for 40-120 min. Samples were taken at time 0 and at 5-min intervals during the first 50 min and 10-min intervals thereafter. Samples were immediately 10-fold serially diluted and plated with the isolation strain for plaque quantification in triplicate.

**Phage DNA-modifying enzymes**

Phages encoding DNA-modifying enzymes were manually curated and annotated. The nucleoside composition of the phages was analysed using high-performance liquid chromatography coupled mass spectrometry (HPLC-MS) on enzymatically hydrolysed DNA. Between 1-2 µg of extracted phage DNA (by phenol-chloroform as described above) was treated with Nucleoside Digestion Mix (New England Biolabs, USA, #M0649) following the manufacturer’s protocol at 37 °C overnight. The nucleoside mixture was filtered through a PTFE 0.2 µm centrifugal filter, and the filtrate was subjected to the reverse phase HPLC-MS for nucleoside separation and detection. LC-MS instrumentation was performed on an Agilent 1290 Infinity II UHPLC-MS system equipped with a G7117 Diode Array Detector and an LC/MSD XT G6135 Single Quadrupole Mass Detector (Agilent, USA). A Waters XSelect HSS T3 C18 column (2.5 µm, 2.1 mm × 100 mm) (Waters, USA) was used for the chromatography and operated at a flow rate of 0.6 mL/min with a binary gradient mobile phase consisting of 10 mM ammonium acetate (pH 4.5) and methanol. The course of chromatography was monitored by UV absorbance of the effluent at 260 nm. Mass spectrometry was operated in both positive (+ESI) and negative (-ESI) electrospray ionization modes. MS was performed with a capillary voltage of 2500 V at both ESI modes, a fragmentor voltage of 70 V, and a mass range of m/z from 100 to 1000. Agilent ChemStation software was used to process primary LC-MS data. Adobe Illustrator was used to compile, render, and annotate the chromatograms exported from ChemStation software.

**Taxonomic classification of RothD**

To identify the closest relatives to RothD, a BLASTn (2) search against the NCBI core-nr database restricted to taxa ID 10239 (virus) was conducted. The top hit with a complete genome (vB_Kpn_Chronis, accession MN013086.1) (3) was compared using Clinker (4) and VIRIDC (5). To confirm the gut-relevance of RothD, we utilized the Gut Phage Database (GPD) (6) retaining only high-quality genomes with a mean ± SD genome completeness of 98.4% ± 2.6 for the creation of a BLASTn (v2.15.0) database (n = 41,427). RothD core genome (1 bp – 20,241 bp) and its full genome were independently searched against the built database with an E-value cutoff of 0.05. Unique hits were retained for taxonomic enquiry (n=355) and used as an input to vContact2 together with RothD and visualised with the igraph package in R v4.4.2. The clustering genomes with RothD (n=132) together with vB_Kpn_Chronis were used as input for VipTree (7) via the webserver selecting for all other dsDNA prokaryotic viruses within their database. The clade containing RothD was selected for further investigation – including 21 genomes from the GPD and a Pantoea phage (PdC23, NC_071008.1). The search against the GPD was supplemented with searches against the IMG/VR database v4.1.1 (8) (high-confidence genomes only) using tBLASTx with an E-value cutoff of 1e-05. Only hits with a genome coverage of $\geq$ 40% to RothD were retained (n=53). Prodigal-gv v2.11.0 (March 2023) (9) was used for open-reading frame annotation of GPD and IMG/VR hits, while gene annotations for vB_Kpn_chronis and Pantoea phage PdC23 were downloaded from GenBank. Genome annotations were done using sequential runs of Pharokka, Phold and Phynteny (10) through a Google Colab notebook (https://colab.research.google.com/github/gbouras13/phold/blob/main/run_pharokka_and_phold_and_phynteny.ipynb). Genome maps and alignments were generated with ggplot2 and gggenes packages (11) in R. All proteins were clustered with MMseqs2 v18.8cc5c (12) with the following parameters: easy-cluster -c 0.7. Clusters containing universal single copy genes (n = 15 clusters) were aligned using Muscle5 v5.2 (13). The produced alignments were concatenated and the final alignment was used to reconstruct the phylogenetic tree with IQTree v2.1 (14) with -B 1000 -mset WAG,LG parameters. The resulting tree was visualised with the ggtree package (15) in R. Complete genome nucleotide comparisons were additionally done using VIRIDIC and ≥45% nucleotide identity to RothD was used for assigning at the subfamily level; ≥70% nucleotide identity was used for assigning at the genus level; and ≥95% was set for same species classification. The taxonomic proposal was submitted to the ICTV on the 27^th^ of June 2024 (16).

**Supplementary Text**

**KlebPhaCol phage characterisation**

The characterisation of KlebPhaCol phages involved genomic, phenotypic, and behavioural analyses, which we organised by genus.

***Gajwadongvirus***

Roth32 is the only *Gajwadongvirus* of the former *Autographiviridae* family (now under an unclassified family) in KlebPhaCol (16). This podophage (Supplementary Text Figure 1A) has a genome size of 46,047 bp (Supplementary Text Figures 2 and 3A) and a burst size of 201±66 particles per lytic cycle (Supplementary Text Figure 3A). Its closest relative was found to be a metagenome-assembled phage with a partial genome, ctVI53 (Genbank: BK053361, Supplementary Text Figure 2A). Roth32 has 62 coding sequences (CDS), of which only 21 are functionally annotated (Supplementary Text Figure 2A). Genome synteny is not well maintained with its relatives, apart from around 29/62 of its CDS, suggesting its uniqueness (Supplementary Text Figure 2A).

Roth32 encodes one of the only four predicted depolymerase enzymes within phages of the collection, Roth32_gp7 (Supplementary Text Figure 4A). This enzyme is homologous to a lyase (PHYRE2: c4y9vA, confidence 80.2, coverage 58%, aa 128-515) and a hydrolase (PHYRE2: c3eqnB, confidence 93.6, coverage 49%, aa 258-576), which is characteristic for depolymerases. Roth32_gp7 shares 57.36%, 57.67% and 57.21% identity on the amino acid level with the depolymerases of the *Klebsiella* phages NTUH-K2044-K1-1 (Genbank: YP_009098385), KpV71 (Genbank: YP_009302756) and GBH001 (Genbank: GBH001_056), respectively, which were all described to be K1-specific (17–19). In agreement with this, Roth32 infects only strain M6, a KL1 strain (Supplementary Table S1, Figure 3A – in LB). Overall, these data suggest that Roth32_gp7 may be a K1-specific depolymerase.

Roth32 had one additional RBP predicted, Roth32_gp59 (Supplementary Text Figure 4E). PHYRE2 (20) analyses showed that it is homologous to the N-terminus of the bacteriophage T7 tail fibre protein gp17 (PHYRE2: c7bozj, confidence 100%, coverage 46%, aa 4-163), which functions as the anchor of the tail fibre to the T7 tail (21), suggesting that Roth32_gp59 may directly interact with the Roth32 tail. Based on high probability alignments to the tailspike protein 4 (TSP4) of phage CBA120 (Genbank: NC_016570.1) via HHPred, we also found that Roth32_gp59 is equipped with a T4 gp10-like branching domain which is a putative docking site for other RBPs (22), where the branching domain sequence is embedded in the 163-306 aa of Roth32_gp59. Therefore, Roth32_gp59 could be an intermediate adaptor protein to which another true RBP can bind (23). These data suggest that the RBP architecture of Roth32 is similar to phages from the previously described KP34 viruses group A (24), where RBP1 (gp59 in Roth32) is truncated and anchors the RBP system as an adaptor protein to the phage tail and provides a branching site for RBP2 (gp7 in Roth32) with depolymerising activity.

***Drulisvirus***

Phages RothG, RothI, and RothJ belong to the *Drulisvirus* genus of the former *Autographiviridae* family, now *Autoscriptoviridae*. These podophages (Supplementary Text Figure 1) have ≈43 kb genomes (Supplementary Text Figure 2B) of high similarity (>99%) and infect the same isolation host, RSUH15, an ST323 KL21 *K. pneumoniae* strain. Phage vB_Ko_K66PH128C1 (Genbank: OY757062) is their closest relative at the time of our search (Supplementary Text Figure 2,3B). RothI and RothJ are more closely related to each other than to RothG. As a result, we selected RothG and RothJ as representatives of this genus for further characterisation. RothG and RothJ have an average burst size of 182±32 and 142±14 particles (Supplementary Text Figure 3B), encoding 62 and 64 CDS of which only 22 and 23, respectively, are annotated (Supplementary Text Figure 2B). Genome synteny is well-kept with their relatives except for a highly variable region with little to no identity between the related phages from gp9-gp15 of RothG, RothJ and RothI (Supplementary Text Figure 2B).

Besides Roth32, RothG, RothI and RothJ are the only phages of the collection encoding RBPs with depolymerase activity (based on high-confidence predictions and manual curations): RothG_gp8, RothI_gp8, and RothJ_gp8 (Supplementary Text Figure 4B-D). These depolymerases share a high aa identity of 98.43%. Their gp8 shares similarity with KL21-targeting proteins of other *Klebsiella* phages (YP_010843553 – 36.83% identity, YP_009198669 – 35.28% identity, YP_003347556 – 35.28% identity to RothG_gp8) (25–27), suggesting that RothG_gp8, RothI_gp8, and RothJ_gp8 may be KL21-specific depolymerases. Indeed, these phages infect KL21 strains of the collection, but they additionally infect KL4 and KL53 strains (Figure 7A). It is possible that gp8 also facilitates KL4 and KL53 targeting, or that the phages encode additional unknown depolymerases that facilitate this expanded host range (Supplementary Table S3) – as is commonly found to be the case for other *Klebsiella* phages (28, 29).

Like Roth32, RothG, RothI and RothJ also encode an additional RBP homologous to the N-terminus of the bacteriophage T7 tail fibre protein gp17 (21), namely RothG_gp62, RothI_gp63, and RothJ_gp64 (Supplementary Text Figure 4F-H). High probability HHPred analyses show the branching domain sequences are embedded in the 191-259 aa of RothG_gp62, RothI_gp63, and RothJ_gp64. As for Roth32_gp59, this suggests that these RBPs are intermediate adaptor proteins to which another RBP can bind, and that the RBP architecture of phages RothG, RothI and RothJ is the same as Roth32 and therefore similar to phages from the previously described KP34 viruses group A (24). The depolymerising activity of the four podophages of the collection is also confirmed by the haloed plaques produced by these phages (Supplementary Text Figure 5A,B).

***Sugarlandvirus***

KlebPhaCol contains four phages of the *Sugarlandvirus* genus (*Demerecviridae* family), Roth37, Roth39, Roth49 and Roth50, with a siphophage morphology (Supplementary Text Figure 1). Their ≈109 kb genomes (Supplementary Text Figure 2C) are highly similar (>99%) with phage DevonBitter (Genbank: OR896848) as their closest relative. Roth39, as the representative phage of this genus, had the highest burst size of all phages in the collection, releasing 524±62 phage particles per cycle (Supplementary Text Figure 3C). The *Sugarlandvirus* also had the highest percentage of hypothetical proteins (~72%) within the phages in the collection, proportional to their genome lengths and encoded genes (~141/196 CDS).

The Roth *Sugarlandvirus* and their relatives maintained a homologous genome architecture, except for phage Spivey (Genbank: MK630230), which has a big portion of its genome inverted, potentially due to assembly issues (Supplementary Text Figure 2C), and phages DevonBitter (Genbank: OR896848) and Torridgeon (Genbank: OR896843), which have a large gene deletion where a tail fibre protein is missing (Supplementary Text Figure 2C). This deletion may represent changes in host range for these phages. Importantly, no DNA-modification enzymes were predicted nor demonstrated for this genus (Supplementary Text Figure 6).

**
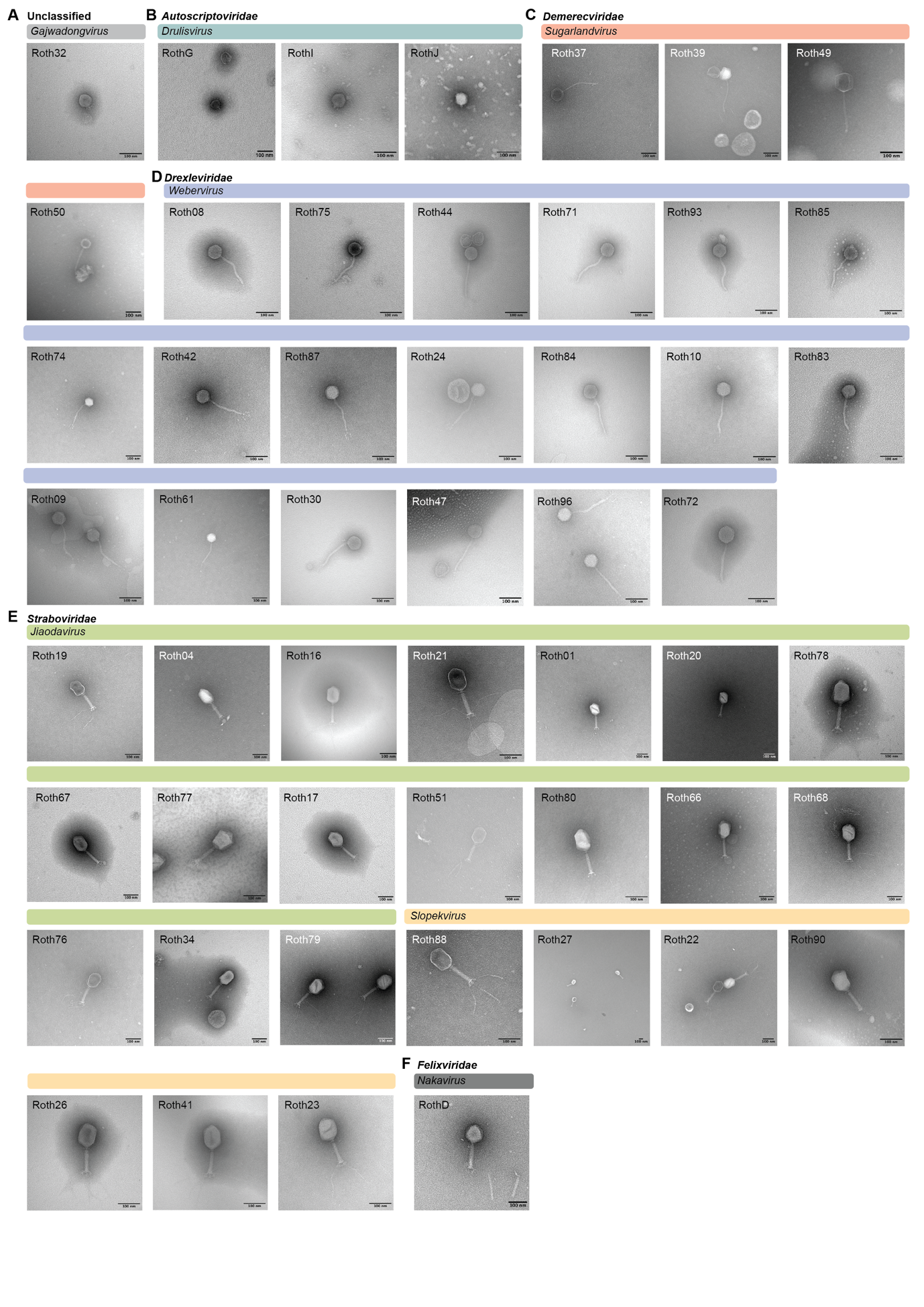
Supplementary Text Figure 1.** Transmission electron microscope images of all KlebPhaCol phages. **(A-F)** Bacteriophages were negatively stained with 5% Ammonium Molybdate (w/v) and 0.1-1% Trehalose. Images were scaled on Fiji, scale bars are for 100 nm.

**
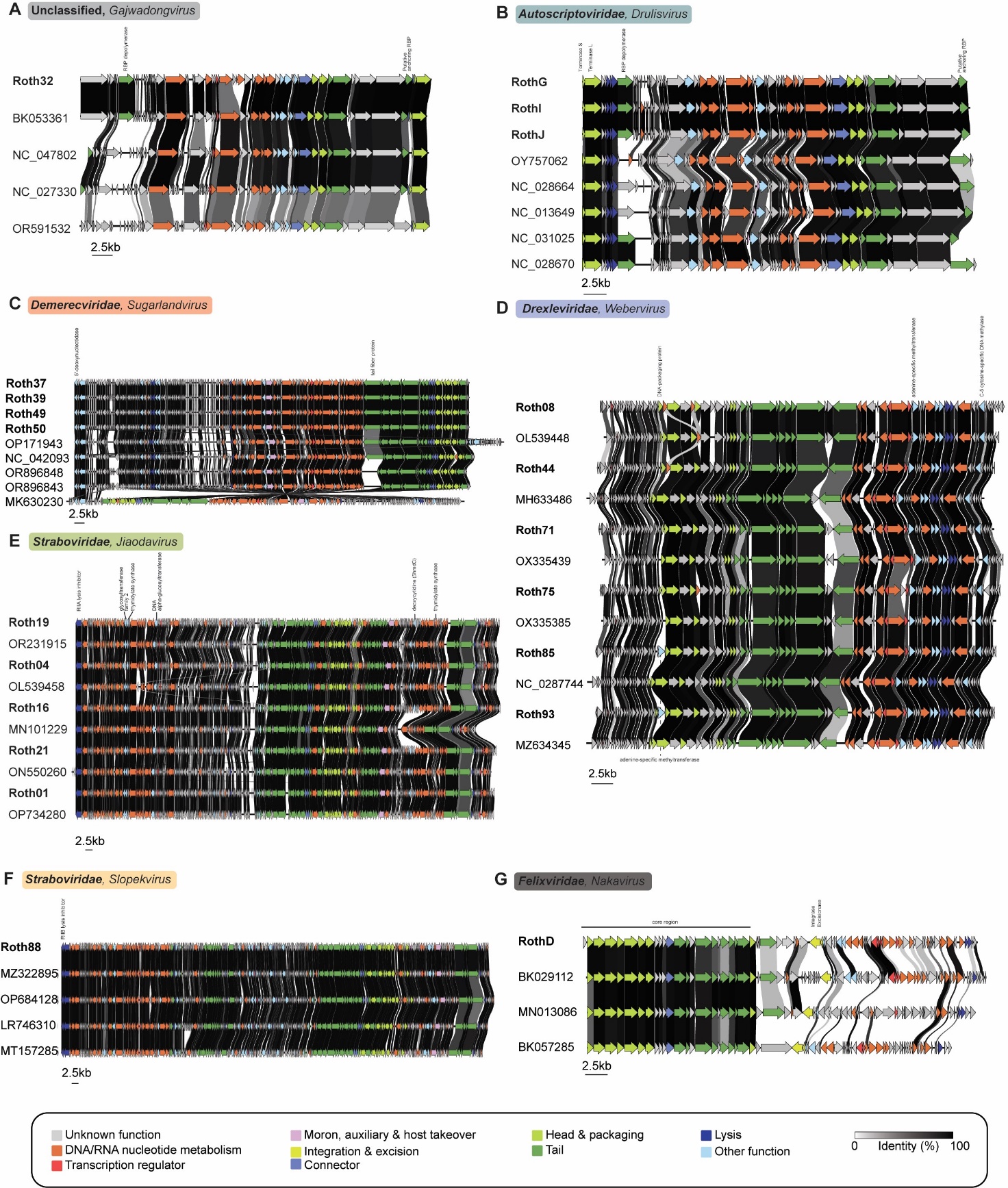
**

**Supplementary Text Figure 2.** Genome synteny plots of representative Roth phages with relatives. **(A-G)** Synteny plots were created by Clinker and genes were coloured by function. If necessary, reference genomes were re-zeroed to match the representative Roth phages genome organization for better comparison.


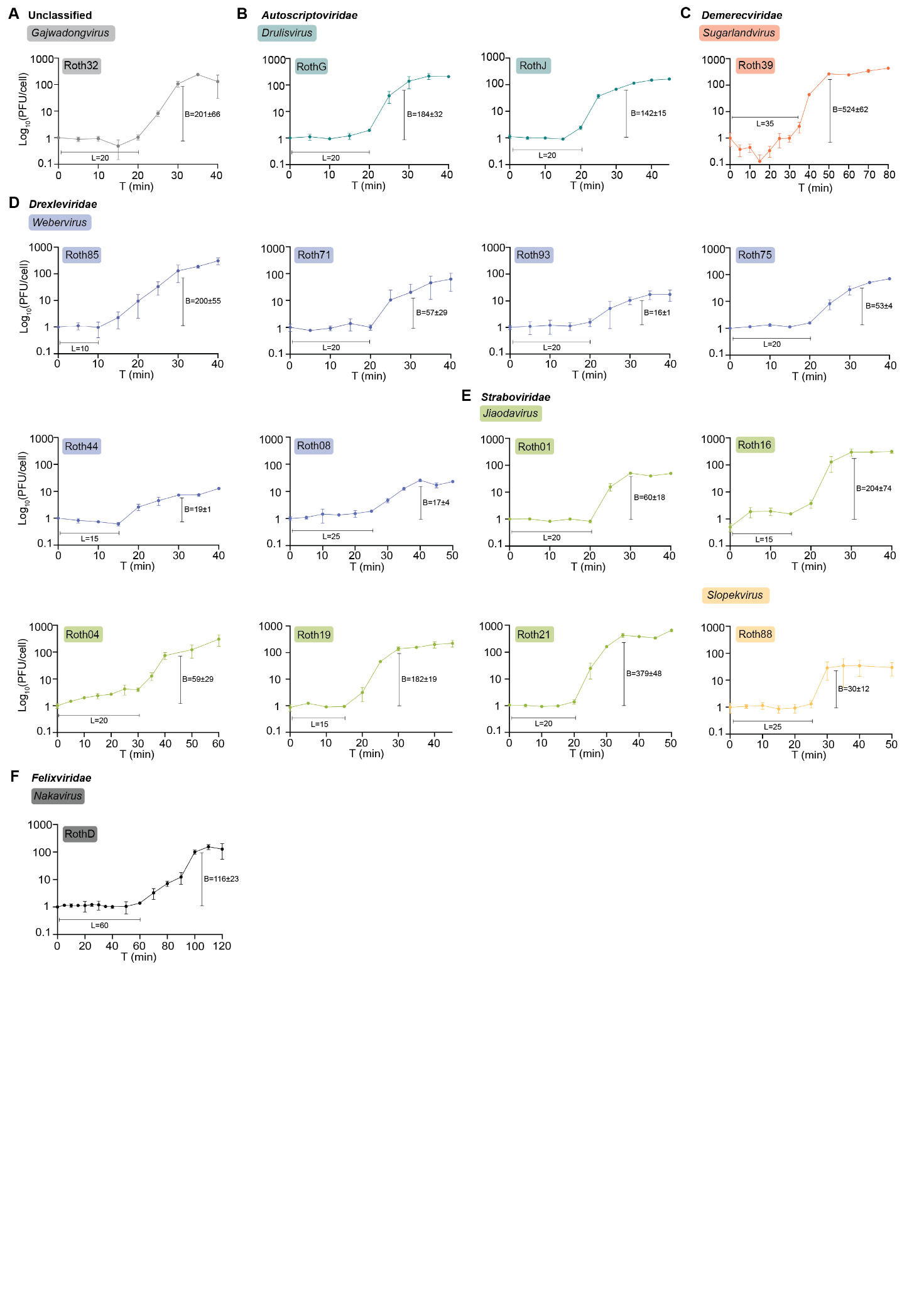
**Supplementary Text Figure 3.** One-step growth curves and burst size of all representative phages. **(A-F)** One-step growth curves were done in triplicate for each phage. Average ± standard deviation of burst sizes (B), and latent period (L) for each curve are also indicated.

**
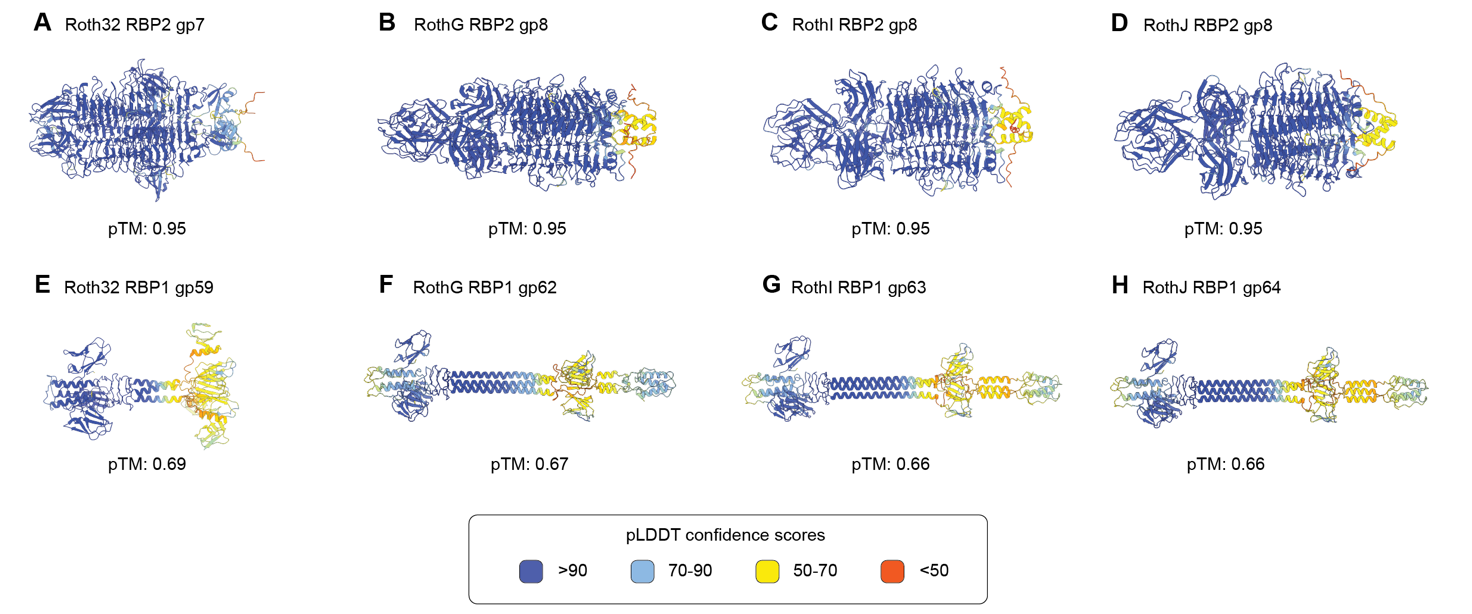
Supplementary Text Figure 4.** Predicted protein structures of computationally curated Receptor Binding Proteins (RBPs) of KlebPhaCol phages **(A-H)** Tetrameric RBP structures predicted with AlphaFold2, coloured by pLDDT confidence scores.

**
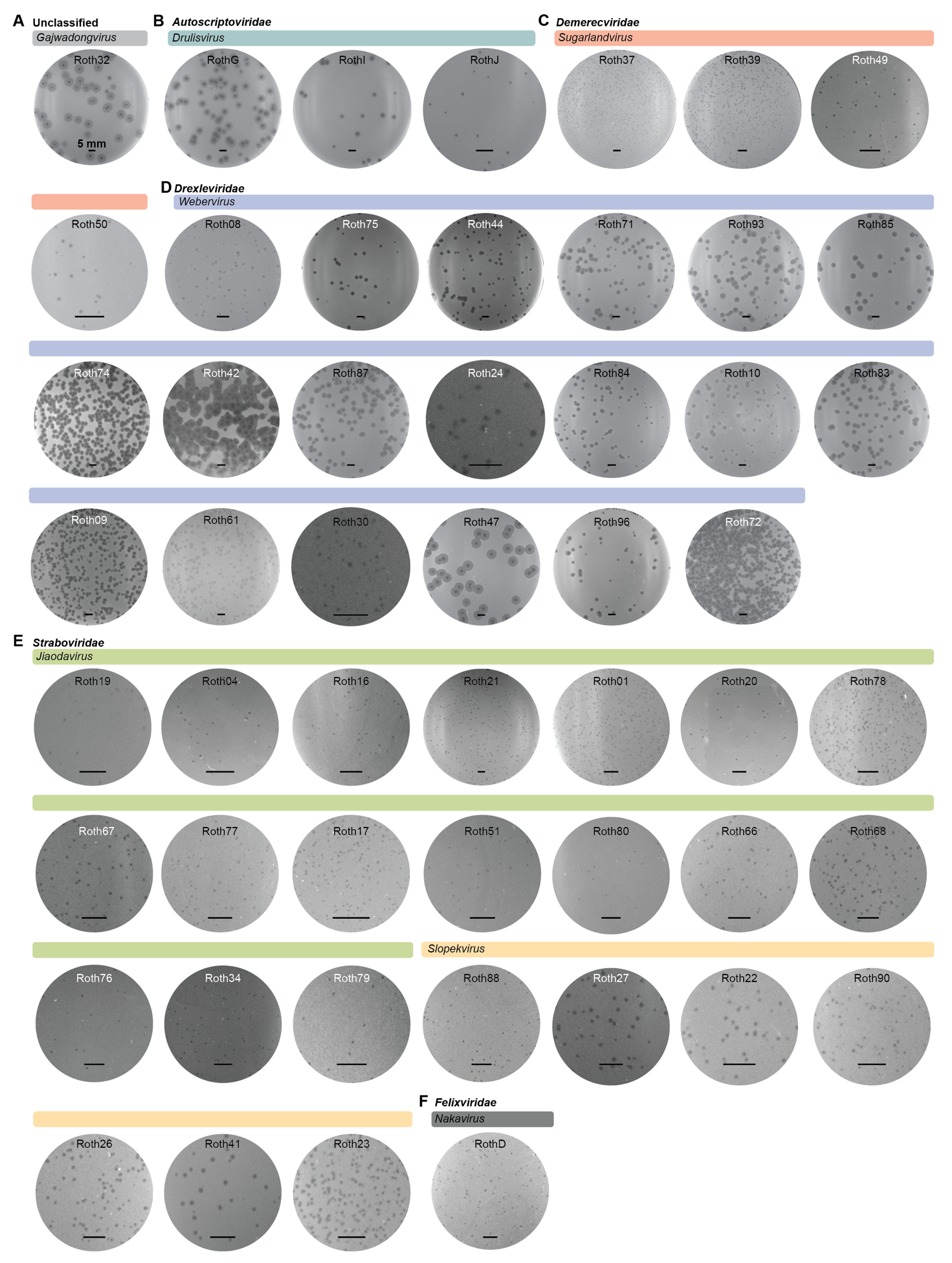
Supplementary Text Figure 5.** Plaque morphologies of all KlebPhaCol phages. **(A-F)** Phages were plated on their isolation host strain – except Roth49 and Roth50, which were plated against NCTC_7427. Images were edited on Fiji, scale is for 5 mm.

***Webervirus***

The largest proportion (n=19/52) of the isolated KlebPhaCol phages belong to the *Webervirus* genus, *Drexleviridae* family. These siphophages (Supplementary Text Figure 1D) have ≈49 kb genomes and are relatively similar to each other ($\geq$86% intergenomic similarity; Supplementary Text Figure 2D), with six representatives selected for further characterisation. These phages show variable burst sizes ranging from 16 virions up to 200 virions released per infection cycle (Supplementary Text Figure 3D). The Roth *Webervirus* encode ≈87 CDS of which ≈29 are annotated. These phages maintained genome synteny with their relatives, with the tail fibre protein having the least shared similarity, and that of Roth93 and PWKp14 (Genbank: MZ634345) being truncated (Supplementary Text Figure 2D). Most phages contained additional CDSs encoding hypothetical proteins between their two tail fibre proteins, some with no shared identity – except for Roth44, which had no additional CDSs in this region (Supplementary Text Figure 2D). We identified five weberviruses that possess a CDS (Roth09_gp33, Roth10_gp33, Roth71_gp34, Roth83_gp33, Roth84_gp33) with significant similarity (E-value $\leq$1e-08) to the putative adhesin Scl1, a bacterial protein known to bind fibronectin type III domains and facilitate colonisation of the ‘superhost’ (30). Recently, other *Klebsiella* weberviruses were found to be associated to the human gut (31), it is therefore possible that these Roth weberviruses can persist in the gut potentially via this protein. However, this requires further investigation. All weberviruses encoded the DNA-modifying enzyme, adenine-specific methyltransferase (AMT), at the 3’ end of their genomes, but phages Roth93 and Roth85 had an additional one (of identical homology) near the 5’ end, which was absent in all other phages included in the genomic comparison (Supplementary Text Figure 2D). Despite this prediction, the respective modification (6mA) was not detected by HPLC analyses (Supplementary Text figure 6A). The presence of adenine methyltransferases in phage genomes has been documented before, notoriously for phages T4 and T2 (32, 33). Their presence in phage genomes is often associated with the regulation of gene expression for several potential functions including i) increasing virulence, ii) evading restriction modification (RM) systems (and potentially other defence systems too (34)), and even iii) switching between lytic-lysogenic switches (35). The roles these may be mediating in Roth phages is currently unknown, and whether they share similarities to those studied from T4-like phages also remains to be investigated (36).

***Jiaodavirus***

The second most abundant phages of KlebPhaCol (n=17/52) belong to the *Jiaodavirus* genus, *Straboviridae* family. These myophages (Supplementary Text Figure 1E) have large genomes of ≈167 kb and have $\geq$91% genome similarity between each other (Supplementary Text Figure 2E). Five representative phages were selected for further characterisation, all of which had variable burst sizes ranging from 60 up to 379 virions released per infection cycle (Supplementary Text Figure 3E). They have a homologous genome synteny to their relatives, but phage KM13 (Genbank: MN101229) has some deletions in the 3’ end before the ribonucleotide reductase gene product, with most of the missing genes encoding for hypothetical proteins but also a restriction endonuclease, RNA ligase, an ACL protein and a deaminase. The *Jiaodavirus* phages encoded the most DNA-modifying enzymes compared to the rest of the phages in the collection, including a glycosyltransferase family 2, a DNA alpha glycosyltransferase and a 5-hydroxymethyl-2’-deoxycytidine, which result in 5hmdC and 5gent-mdC DNA modifications as confirmed by HPLC (Supplementary Text Figures 2E and 6). These are commonly found enzymes in multiple phage genomes including phage T4, where their functions have been extensively studied (37). In T4, they protect the viral DNA from degradation by host-encoded nucleases and restriction enzymes, as well as evasion of host defence systems; they also target the host genome for degradation instead, allowing some DNA components to be reused for DNA synthesis (38). Collectively, these roles contribute to T4’s high virulence and selective advantage. The presence of these multiple DNA-modifying enzymes could partially explain the high efficiency of Roth jiaodaviruses.

**
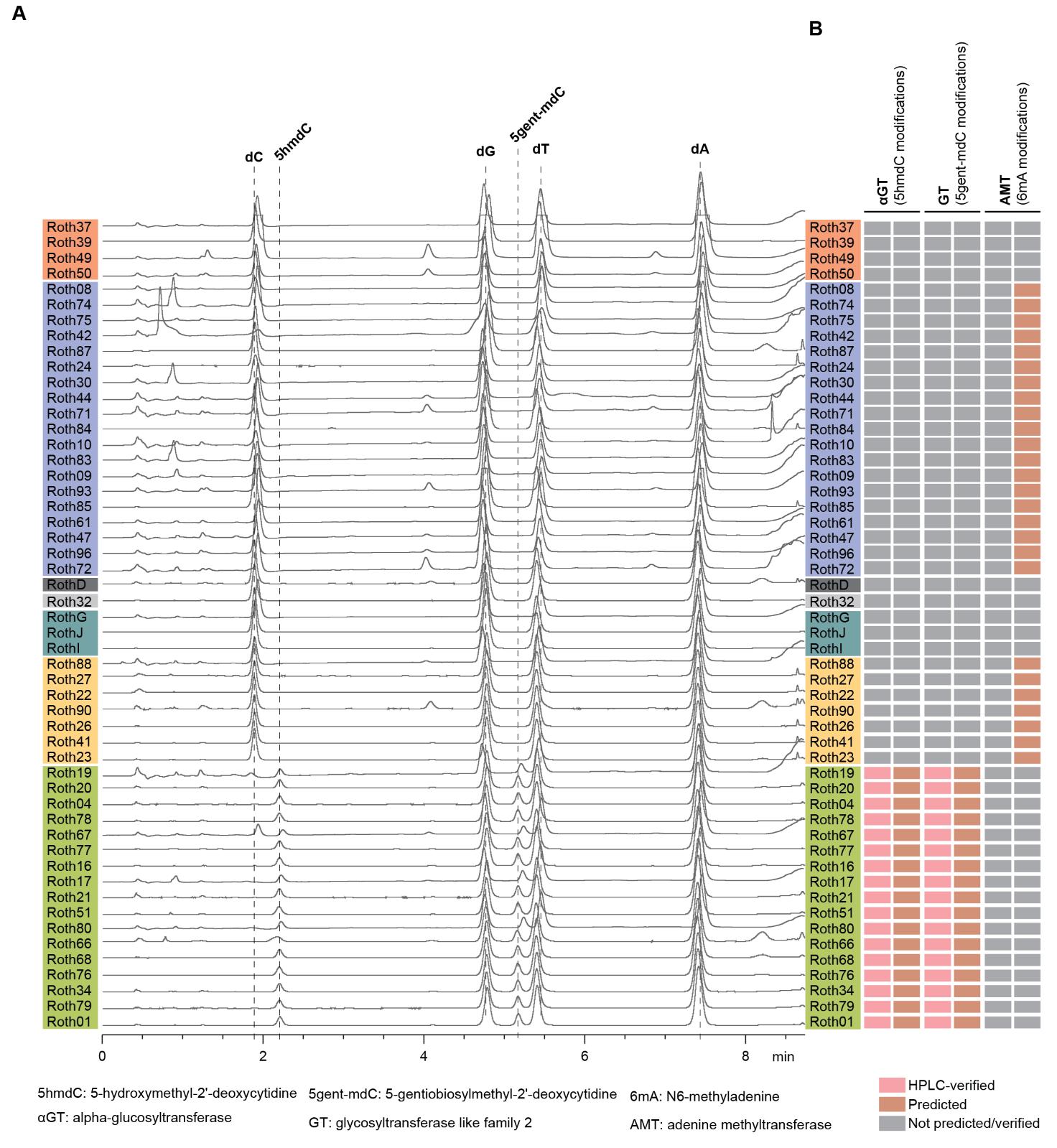
**

**Supplementary Text Figure 6.** DNA-modifying enzymes of Roth phages. **(A)** The DNA-encoded DNA modification enzymes were verified by HPLC for all 52 phages. **(B)** Summarised findings of DNA-modifying enzymes from the HPLC findings (lighter pink box) and from genome annotation predictions (dark pink boxes).

***Slopekvirus***

Seven phages of the KlebPhaCol belong to the *Slopekvirus* genus, *Straboviridae* family. These myophages (Supplementary Text Figure 1E) have the largest genomes in the collection (≈174 kb) and high intergenomic similarity (>99%; Supplementary Text Figure 2E). Their closest relative at the time of our search was phage KpnM_VAC13 (Genbank: MZ322895). Roth88 was selected as a representative *Slopekvirus* and has a burst size of 30±12 virions per infection cycle (Supplementary Text Figure 3E). Roth88 maintained a highly homologous genome synteny with its relatives. The genes with most notable differences were tail proteins, likely with impact in the phage host range. Another notable difference was phage P-PK2 (Genbank: MT157285), with a slightly shorter genome due to a deletion of a mid-genome region of small CDSs annotated as hypothetical proteins. Interestingly, each phage may have two putative depolymerases with an unusual non-beta-helical shape, requiring further experimental validation (Supplementary Table S3). Like *Webervirus,* the *Slopekvirus* also encoded the DNA-modification enzyme AMT (Supplementary Text Figure 6).

**New *Nakavirus* genus**

Following ICTV genome-based guidelines, we conducted a BLASTn search of RothD against the NCBI database; however, none of the top 10 hits corresponded to closely related phages. After restricting the search to the virus taxa ID (10239), the closest matches were an incomplete metagenomic phage assembly: ctlJz2 (Genbank: BK029112) (39), and the previously cultured but unclassified phage vB_Kpn_Chronis (Genbank: MN013086) (3). Due to its complete genome, only phage vB_Kpn_Chronis, was selected for further analysis.

Because RothD targets gut-associated ST323 *K. pneumoniae*, we expanded the search to the Gut Phage Database (GPD) (6) using BLASTn and considering only high-quality genomes that were ≥90% complete. Hits were then clustered to RothD and vB_Kpn_Chronis with vContact2, retaining 132 phages (Supplementary Text Figure 7A). These hits shared a mean SD query coverage of 17.6 +_ 14.4% to RothD (Supplementary Table S7). We identified 19 genomes forming a tight cluster with RothD and covering at least 21% of its genome. We proposed that these phages may constitute a distinct family. To validate this observation, we performed a tBLASTx search against the full set of 132 genomes, which confirmed that the selected genomes share substantially higher similarity to RothD than other genomes from the GPD cluster (Supplementary Text Figure 7B). For further taxonomic classification, we retained only genomes covering at least 40% of the RothD genome and subsequently proposed a new family, *Felixviridae* (Supplementary Text Figure 7C). Based on a demarcation criteria of ≥ 45%, we proposed the new subfamily *Maevirinae* to include RothD and vB_Kpn_Chronis (Supplementary Text Figure 7C). Following a nucleotide identity threshold of ≥70%, we suggest the *Nakavirus* genus for RothD represented by a single species, *Nakavirus sapi*; and *Chronisvirus* for vB_Kpn_Chronis that currently comprises the single species, *Chronisvirus chronis*. To gain further resolution within *Felixviridae*, we expanded our search to the IMG/VR database with RothD as query. This allowed us to extend the number of genomes belonging to *Felixviridae* to 53 phages. To reconstruct phylogeny within the family, we clustered the proteomes of the 53 phages that included members from the GPD, IMG/VR, and isolates vB_Kpn_Chronis, and Pantoea phage PdC23. Our analysis revealed that the phages share 15 core proteins, most of which are structural genes (Supplementary Text Figure 7D). With an average CDS capacity of ~70 genes, this represents ~21% of their genome, sustaining the proposed family. Other hits falling outside the family cutoff do not share the structural portion of the genome and have completely different capsids, yet they often carry non-structural genes or even integrases similar to RothD (Supplementary Text Figure 8A). All members of the proposed family are likely temperate phages and are mostly found as prophages in *Enterobacteriaceae* genomes. Therefore, it is surprising that they do not necessarily share the same integrase (Supplementary Text Figure 8B). This pattern – conserved structural genes combined with frequent variation in other genes – suggests that members of the family likely undergo frequent homologous recombination.

**
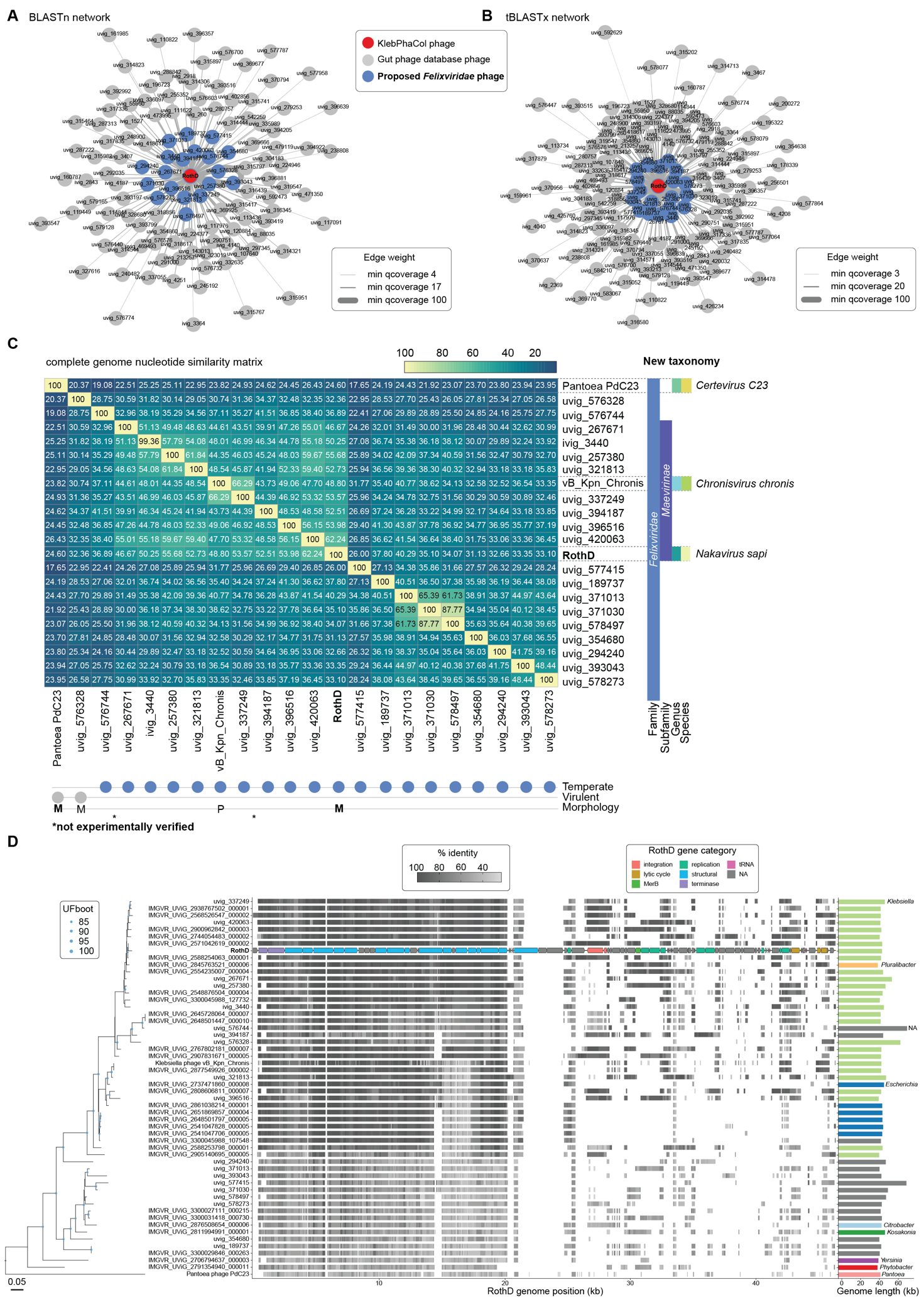
**

**Supplementary Text Figure 7.** Taxonomic classification for RothD – *Felixviridae* family. **(A)** Network of RothD clustering with high confidence to phages from the Gut Phage Database. The edge length is proportional to RothD genome coverage by BLASTn search. Phages that ultimately belonged to the proposed *Felixviridae* family are highlighted in blue. **(B)** Network of the same phages as in (A) but showing proportional edge lengths to RothD’s genome calculated by tBLASTx. **(C)** Whole genome nucleotide similarity from phages in (B) created with VIRIDIC. Phage lifestyles predicted by Bacphlip are shown below. Morphology was taken from the literature for reference phages (PdC23 and vB_Kpn_Chronis), assessed by TEM for the Roth phages, and where data was available, taken from the Gut Phage Database metadata for the remaining phages (only for available for uvig_576328). M – Myophage; P – Podophage. **(D)** Core proteome (n = 15) tree of all phages in (C) with additional hits (tBLASTx, $\geq$ 40% query coverage to RothD, n = 32) from the IMG/VR database. Genome similarity is compared to RothD, predicted hosts to the right.


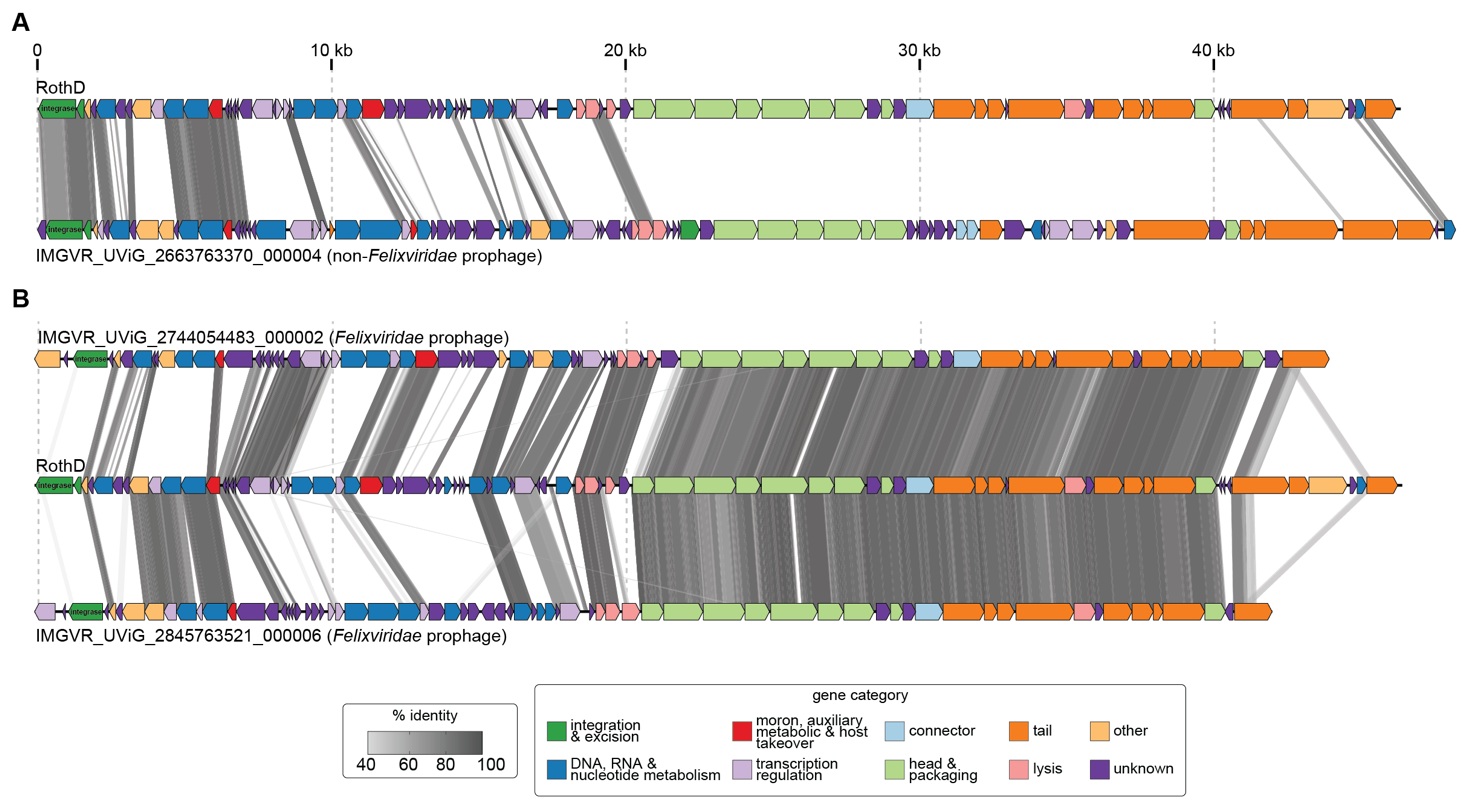


**Supplementary Text Figure 8.** *Felixviridae* and related phages undergo frequent recombination. **(A)** Pairwise comparison of RothD and IMGVR_UViG_2663763370_000004, a prophage outside of the *Felixviridae* family, showing different structural genes but similar cargo proteins. **(B)** Pairwise comparison of RothD and two phages within *Felixviridae* with different integrases.

**Supplementary references**

1. Schindelin,J., Arganda-Carreras,I., Frise,E., Kaynig,V., Longair,M., Pietzsch,T., Preibisch,S., Rueden,C., Saalfeld,S., Schmid,B., *et al.* (2012) Fiji: an open-source platform for biological-image analysis. *Nature Methods 2012 9:7*, **9**, 676–682.

2. Altschul,S.F., Madden,T.L., Schäffer,A.A., Zhang,J., Zhang,Z., Miller,W. and Lipman,D.J. (1997) Gapped BLAST and PSI-BLAST: A new generation of protein database search programs. *Nucleic Acids Res*, **25**, 3389–3402.

3. Thurgood,T.L., Sharma,R., Call,J.J., Chronis,J.D., Dawson,D.D., Finnegan,Z.K., Foster,K.W., Meek,T., Potts,E., Sirrine,M.R., *et al.* (2020) Genome Sequences of 12 Phages That Infect Klebsiella pneumoniae. *Microbiol Resour Announc*, **9**.

4. Gilchrist,C.L.M. and Chooi,Y.H. (2021) clinker & clustermap.js: automatic generation of gene cluster comparison figures. *Bioinformatics*, **37**, 2473–2475.

5. Moraru,C., Varsani,A. and Kropinski,A.M. (2020) VIRIDIC—A Novel Tool to Calculate the Intergenomic Similarities of Prokaryote-Infecting Viruses. *Viruses 2020, Vol. 12, Page 1268*, **12**, 1268.

6. Camarillo-Guerrero,L.F., Almeida,A., Rangel-Pineros,G., Finn,R.D. and Lawley,T.D. (2021) Massive expansion of human gut bacteriophage diversity. *Cell*, **184**, 1098-1109.e9.

7. Nishimura,Y., Yoshida,T., Kuronishi,M., Uehara,H., Ogata,H. and Goto,S. (2017) ViPTree: the viral proteomic tree server. *Bioinformatics*, **33**, 2379–2380.

8. Camargo,A.P., Nayfach,S., Chen,I.M.A., Palaniappan,K., Ratner,A., Chu,K., Ritter,S.J., Reddy,T.B.K., Mukherjee,S., Schulz,F., *et al.* (2023) IMG/VR v4: an expanded database of uncultivated virus genomes within a framework of extensive functional, taxonomic, and ecological metadata. *Nucleic Acids Res*, **51**, D733–D743.

9. Hyatt,D., Chen,G.L., LoCascio,P.F., Land,M.L., Larimer,F.W. and Hauser,L.J. (2010) Prodigal: Prokaryotic gene recognition and translation initiation site identification. *BMC Bioinformatics*, **11**, 1–11.

10. Bouras,G., Grigson,S.R., Mirdita,M., Heinzinger,M., Papudeshi,B., Mallawaarachchi,V., Green,R., Kim,R.S., Mihalia,V., Psaltis,A.J., *et al.* (2025) Protein Structure Informed Bacteriophage Genome Annotation with Phold. *bioRxiv*, 10.1101/2025.08.05.668817.

11. Hadley Wickham ggplot2: Elegant Graphics for Data Analysis. *J R Stat Soc Ser A Stat Soc*, **174**, 245–246.

12. Steinegger,M. and Söding,J. (2017) MMseqs2 enables sensitive protein sequence searching for the analysis of massive data sets. *Nat Biotechnol*, **35**, 1026–1028.

13. Edgar,R.C. (2022) Muscle5: High-accuracy alignment ensembles enable unbiased assessments of sequence homology and phylogeny. *Nature Communications 2022 13:1*, **13**, 1–9.

14. Minh,B.Q., Schmidt,H.A., Chernomor,O., Schrempf,D., Woodhams,M.D., Von Haeseler,A., Lanfear,R. and Teeling,E. (2020) IQ-TREE 2: New Models and Efficient Methods for Phylogenetic Inference in the Genomic Era. *Mol Biol Evol*, **37**, 1530–1534.

15. Yu,G., Smith,D.K., Zhu,H., Guan,Y. and Lam,T.T.Y. (2017) ggtree: an r package for visualization and annotation of phylogenetic trees with their covariates and other associated data. *Methods Ecol Evol*, **8**, 28–36.

16. Turner,D., Adriaenssens,E.M., Amann,R.I., Bardy,P., Bartlau,N., Barylski,J., Błażejak,S., Bouzari,M., Briegel,A., Briers,Y., *et al.* (2025) Summary of taxonomy changes ratified by the International Committee on Taxonomy of Viruses (ICTV) from the Bacterial Viruses Subcommittee, 2025. *J Gen Virol*, **106**.

17. Solovieva,E. V., Myakinina,V.P., Kislichkina,A.A., Krasilnikova,V.M., Verevkin,V. V., Mochalov,V. V., Lev,A.I., Fursova,N.K. and Volozhantsev,N. V. (2018) Comparative genome analysis of novel Podoviruses lytic for hypermucoviscous Klebsiella pneumoniae of K1, K2, and K57 capsular types. *Virus Res*, **243**, 10–18.

18. Blundell-Hunter,G., Enright,M.C., Negus,D., Dorman,M.J., Beecham,G.E., Pickard,D.J., Wintachai,P., Voravuthikunchai,S.P., Thomson,N.R. and Taylor,P.W. (2021) Characterisation of Bacteriophage-Encoded Depolymerases Selective for Key Klebsiella pneumoniae Capsular Exopolysaccharides. *Front Cell Infect Microbiol*, **11**, 686090.

19. Lin,T.L., Hsieh,P.F., Huang,Y.T., Lee,W.C., Tsai,Y.T., Su,P.A., Pan,Y.J., Hsu,C.R., Wu,M.C. and Wang,J.T. (2014) Isolation of a Bacteriophage and Its Depolymerase Specific for K1 Capsule of Klebsiella pneumoniae: Implication in Typing and Treatment. *J Infect Dis*, **210**, 1734–1744.

20. Kelley,L.A., Mezulis,S., Yates,C.M., Wass,M.N. and Sternberg,M.J.E. (2015) The Phyre2 web portal for protein modeling, prediction and analysis. *Nat Protoc*, **10**, 845–858.

21. Steven,A.C., Trus,B.L., Maizel,J. V., Unser,M., Parry,D.A.D., Wall,J.S., Hainfeld,J.F. and Studier,F.W. (1988) Molecular substructure of a viral receptor-recognition protein: The gp17 tail-fiber of bacteriophage T7. *J Mol Biol*, **200**, 351–365.

22. Prokhorov,N.S., Riccio,C., Zdorovenko,E.L., Shneider,M.M., Browning,C., Knirel,Y.A., Leiman,P.G. and Letarov,A. V. (2017) Function of bacteriophage G7C esterase tailspike in host cell adsorption. *Mol Microbiol*, **105**, 385–398.

23. Plattner,M., Shneider,M.M., Arbatsky,N.P., Shashkov,A.S., Chizhov,A.O., Nazarov,S., Prokhorov,N.S., Taylor,N.M.I., Buth,S.A., Gambino,M., *et al.* (2019) Structure and Function of the Branched Receptor-Binding Complex of Bacteriophage CBA120. *J Mol Biol*, **431**, 3718–3739.

24. Latka,A., Leiman,P.G., Drulis-Kawa,Z. and Briers,Y. (2019) Modeling the Architecture of Depolymerase-Containing Receptor Binding Proteins in Klebsiella Phages. *Front Microbiol*, **10**, 2649.

25. Lukianova,A.A., Shneider,M.M., Evseev,P. V., Egorov,M. V., Kasimova,A.A., Shpirt,A.M., Shashkov,A.S., Knirel,Y.A., Kostryukova,E.S. and Miroshnikov,K.A. (2023) Depolymerisation of the Klebsiella pneumoniae Capsular Polysaccharide K21 by Klebsiella Phage K5. *Int J Mol Sci*, **24**, 17288.

26. Majkowska-Skrobek,G., Latka,A., Berisio,R., Squeglia,F., Maciejewska,B., Briers,Y. and Drulis-Kawa,Z. (2018) Phage-Borne Depolymerases Decrease Klebsiella pneumoniae Resistance to Innate Defense Mechanisms. *Front Microbiol*, **9**.

27. Pan,Y.-J., Lin,T.-L., Chen,C.-C., Tsai,Y.-T., Cheng,Y.-H., Chen,Y.-Y., Hsieh,P.-F., Lin,Y.-T. and Wang,J.-T. (2017) Klebsiella Phage ΦK64-1 Encodes Multiple Depolymerases for Multiple Host Capsular Types. *J Virol*, **91**.

28. Hsieh,P.F., Lin,H.H., Lin,T.L., Chen,Y.Y. and Wang,J.T. (2017) Two T7-like Bacteriophages, K5-2 and K5-4, Each Encodes Two Capsule Depolymerases: Isolation and Functional Characterization. *Scientific Reports 2017 7:1*, **7**, 1–13.

29. Majkowska-Skrobek,G., Latka,A., Berisio,R., Squeglia,F., Maciejewska,B., Briers,Y. and Drulis-Kawa,Z. (2018) Phage-borne depolymerases decrease Klebsiella pneumoniae resistance to innate defense mechanisms. *Front Microbiol*, **9**, 419180.

30. McNitt,D.H., Choi,S.J., Allen,J.L., Hames,R.A., Weed,S.A., Van De Water,L., Berisio,R. and Lukomski,S. (2019) Adaptation of the group A Streptococcus adhesin Scl1 to bind fibronectin type III repeats within wound-associated extracellular matrix: implications for cancer therapy. *Mol Microbiol*, **112**, 800–819.

31. Dawson,S.J.T., Shibu,P., Garnett,S., Newberry,F., Brook,T.C., Tijani,T., Kujawska,M., Hall,L.J., McCartney,A.L., Negus,D., *et al.* (2025) Weberviruses are gut-associated phages that infect Klebsiella spp. *FEMS Microbiol Ecol*, **101**, 43.

32. Schlagaman,S.L. and Hattman,S. (1989) The bacteriophage T2 and T4 DNA-[N6-adenine] methyltransferase (Dam) sequence specificities are not identical. *Nucleic Acids Res*, **17**, 9101.

33. Lehman,I.R. and Pratt,E.A. (1960) On the Structure of the Glucosylated Hydroxymethylcytosine Nucleotides of Coliphages T2, T4, and T6. *Journal of Biological Chemistry*, **235**, 3254–3259.

34. Went,S.C., Picton,D.M., Morgan,R.D., Nelson,A., Brady,A., Mariano,G., Dryden,D.T.F., Smith,D.L., Wenner,N., Hinton,J.C.D., *et al.* (2024) Structure and rational engineering of the PglX methyltransferase and specificity factor for BREX phage defence. *Nature Communications 2024 15:1*, **15**, 1–18.

35. Bochow,S., Elliman,J. and Owens,L. (2012) Bacteriophage adenine methyltransferase: A life cycle regulator? Modelled using Vibrio harveyi myovirus like. *J Appl Microbiol*, **113**, 1001–1013.

36. Yang,Z., Horton,J.R., Zhou,L., Zhang,X.J., Dong,A., Zhang,X., Schlagman,S.L., Kossykh,V., Hattman,S. and Cheng,X. (2003) Structure of the bacteriophage T4 DNA adenine methyltransferase. *Nat Struct Biol*, **10**, 849.

37. Pyle,J.D., Lund,S.R., O’Toole,K.H. and Saleh,L. (2024) Virus-encoded glycosyltransferases hypermodify DNA with diverse glycans. *Cell Rep*, **43**, 114631.

38. Sommer,N., Depping,R., Piotrowski,M. and Rüger,W. (2004) Bacteriophage T4 α-glucosyltransferase: a novel interaction with gp45 and aspects of the catalytic mechanism. *Biochem Biophys Res Commun*, **323**, 809–815.

39. Tisza,M.J. and Buck,C.B. (2021) A catalog of tens of thousands of viruses from human metagenomes reveals hidden associations with chronic diseases. *Proc Natl Acad Sci U S A*, **118**, e2023202118.
